
JOURNAL INFORMATION
==============================
NLM Title Abbreviation: J Diabetes Investig
Journal ID: J Diabetes Investig
NLM Journal ID: 101520702
Journal ID: JDI

Journal of Diabetes Investigation

ISSN: 2040-1116
EISSN: 2040-1124
Publisher: Wiley

ARTICLE INFORMATION
==============================
PMCID: PMC4906258
DOI: 10.1111/jdi.12509
Article ID: JDI12509
Article version: 1
Subjects: Abstracts; Abstracts from The 7th Scientific Meeting of the Asian Association for the Study of Diabetes and the Annual Scientific Meeting of the Hong Kong Society of Endocrinology, Metabolism and Reproduction, 21‐22 November 2015, Hong Kong, China; Abstracts

Abstracts

Electronic publication date: 2016 Jun 14
Print publication date: 2016 May
Volume: 7
Issue: Suppl 2
Issue title: Abstracts from The 7th Scientific Meeting of the Asian Association for the Study of Diabetes and the Annual Scientific Meeting of the Hong Kong Society of Endocrinology, Metabolism and Reproduction, 21‐22 November 2015, Hong Kong, China
First page: 11
Last page: 55
Copyright: © 2016 The Authors. Journal of Diabetes Investigation published by Asian Association for the Study of Diabetes (AASD) and John Wiley & Sons Australia, Ltd
License: This is an open access article under the terms of the Creative Commons Attribution License, which permits use, distribution and reproduction in any medium, provided the original work is properly cited.
License URL: https://creativecommons.org/licenses/by/4.0/

Page count: 45
source-schema-version-number: 2.0
component-id: jdi12509
cover-date: May 2016
details-of-publishers-convertor: Converter:WILEY_ML3GV2_TO_NLMPMC version:4.9.1 mode:remove_FC converted:14.06.2016

==============================
JDI Journal of Diabetes Investigation Open access
Official Journal of the Asian Association for the Study of Diabetes

### 0001
### High cystatin C levels predict undesirable outcome for diabetic foot ulcerations

Clinical Diabetes

A. Ligen[1], Z. Xiaoyan[1], Z. Hui[2], Z. Jun[3], Z. Jungong[4], C. Yimin[5], L. Junxi[1], T. Junling[1], B. Yuqian[1], L. Fang[1,*] and J. Weiping[1,*]

*[1]Master degree candidate of Shanghai Jiao Tong University Medical School, Shanghai Key Laboratory of Diabetes Mellitus, Shanghai Clinical Medical Center of Diabetes, Shanghai Key Clinical Center of Met, Shanghai, China, [2]Shanghai Clinical Medical Center of Diabetes, Shanghai Key Clinical Center of Metabolic Disease, Shanghai Institute for Diabetes, Department of Endocrinology & Metabolism, Shanghai Jiao Tong University, Shanghai, China, [3]The Multi-disciplinary Collaborative Diabetic Foot Group of Shanghai Clinical Center of Diabetes, [2]Department of Vascular Surgery, Shanghai Jiao Tong University Affiliated Sixth People's Hospital, Shanghai, China, [4]The Multi-disciplinary Collaborative Diabetic Foot Group of Shanghai Clinical Center of Diabetes, [3]Department of Interventional Radiology, Shanghai Jiao Tong University Affiliated Sixth People's Hospital, Shanghai, China, [5]The Multi-disciplinary Collaborative Diabetic Foot Group of Shanghai Clinical Center of Diabetes, [4]Department of Orthopedics, Shanghai Clinical Center of Trauma and Orthopedics, Shanghai Jiao Tong University, Shanghai, China*

Objectives: Cystatin C is growing to be an ideal indicator for renal function and cardiovascular events. The aim of this study was to investigate the relationship between serum Cystatin C levels and the prognosis of diabetic foot ulcerations (DFU).

Method: Totally 1012 patients with Type 2 diabetes were recruited and divided into two groups: non-diabetic-foot group (NDF, $n$ = 865, 85.5%) and diabetic foot ulcer group (DFU, $n$ = 147, 14.5%). After 1 year follow-up, the patients with DFU were grouped into healing group ($n$ = 110, 74.8%) and non-healing group ($n$ = 37, 25.2%) according to the clinical prognosis. Clinical characteristics and the impact factors of ulcer healing were compared and analyzed.

Results: Compared to the cured group, patients in non-healing group were older, had long diabetic duration and significantly higher serum Cystatin C concentrations in DFU ($P < 0.01$). Multiple logistical regression analysis revealed that Cystatin C was an independent impact factor for non-healing rate of DFU (b =—1.277, $P < 0.01$). After adjusting for age, duration, renal function, Cystatin C was still linked with increased risk of undesirable DFU outcome (OR = 7.279, 95% CI: 1.299–40.784, $P < 0.05$). By dividing patients into quartiles according to Cystatin C, a downward trend in healing rate was found with increased quartile of serum Cystatin C, there are significant lower healing rate was found in fourth group which was only 57.9% compared with other three groups ($P < 0.01$). The oddis ratio (OR) analysis showed that the risk of undesirable outcome of DFU in Quartile 4 increased significantly (OR = 4.554, 95% CI: 3.14–5.12, $P < 0.05$) compared with that of Quartile 1. ROC analysis further indicated that the optimal cutoff of Cystatin C to predict the poor prognosis of DFU was 1.35 mg/L ($P < 0.01$) with 79.3% of sensitivity and nearly 62% of specificity.

Conclusions: There is a strong and independent association between serum Cystatin C and diabetic foot ulceration prognosis, and Cystatin C > 1.35 mg/L predicts more than 6-fold increased risk of incurable foot ulceration.

### 0002
### Associations of serum adipocyte fatty acid binding protein with carotid intima-media thickness in Chinese women with normal glucose tolerance

Macrovascular complications

Y. Hao, X. Ma, Y. Luo, Y. Shen, X. Pan, Y. Bao and W. Jia

*Department of Endocrinology and Metabolism, Shanghai Jiao Tong University Affiliated Sixth People's Hospital; Shanghai Clinical Center for Diabetes; Shanghai Key Clinical Center for Metabolic Disease; Shanghai, China*

Objectives: Recent studies highlight a critical interaction between adipocyte fatty acid binding protein (A-FABP) and cardiovascular disorders. However, relationships between serum A-FABP with subclinical atherosclerosis in normal glucose tolerance subjects remain unknown. The study aimed to evaluate the association of serum A-FABP with carotid intima-media thickness (C-IMT) in a Chinese population with normal glucose tolerance.

Method: 2253 cardiovascular disease-free participants with normal glucose tolerance aged from 20 to 78 years old (835 men, 1418 women) from the Shanghai Obesity Study were enrolled. Serum A-FABP levels were measured by a sandwich enzyme-linked immunosorbent assay. B-mode ultrasound was used to assess C-IMT.

Results: Serum A-FABP level was significantly higher in women than men ($P < 0.001$). Partial correlation analysis showed that serum A-FABP was associated with C-IMT in men, premenopausal and postmenopausal women after adjusting for age and body mass index (BMI) ($P$ = 0.024, 0.006, and 0.016, respectively). Moreover, C-IMT increased along with quartile A-FABP values (all $P$ for trend <0.001). Regression analyses demonstrated that A-FABP independently correlated with C-IMT only in women ($P$ = 0.044 and 0.001 for pre- and postmenopausal, respectively), but not in men. Furthermore, A-FABP was identified as a risk factor for C-IMT in pre- and postmenopausal women with BMI <25 kg/m$^2$ ($P < 0.001$ and $P$ = 0.012, respectively).

Conclusions: Serum A-FABP level independently correlated with C-IMT in pre- and postmenopausal Chinese women with normal glucose tolerance.

### 0003
### Declined task performance in a patient with newly diagnosed type 2 diabetes mellitus

Clinical Diabetes

S. Koga, H. Saito and K. Takazawa

*Section for Disorders in Endocrine and Kidney System, Department of Medicine, Tokyo Metropolitan Police Hospital, Tokyo, Japan*

Background: Recently, type 2 diabetes patients have reported to be reduced 1) activation of the dorsolateral prefrontal cortex (PFC) during task encoding and 2) deactivation of the default mode network (DMN) during task recognition, despite normal cognition;During recognition, activation in the dorsolateral PFC and the DMN regions is positively correlated with HbA1c and insulin resistance, suggesting that these important markers of glucose metabolism impact the brain's response to a cognitive challenge 1).

Case record : A 62-year-old BMI 25.4 female with nonalcoholic steatohepatitis and obstructive sleep apnea syndrome was brought to emergency department with chief complaint of palpitation, dizziness, and fasting anxiety. She had no habitual smoking nor drinking. Lab data show moderately elevated transaminases, gGTP, and dip stick urine protein. Diagnosis of type 2 diabetes was made of plasma glucose 14.1 mmol/L, HbA1c 6.2%, HOMA-b 22.8%, HOMA-R 7.59, and urine C-peptide 144 mcg/day. After metformin was updosed to 500 mg bid, dipeptidyl dipeptidase IV (DPP-IV) inhibitor sitagliptin 50 mg sid was added and paroxetin 20 mg sid was started to control anxiety. HbA1c was constantly decreased to 5.2% in two months but she similarly complained of anxiety described above; finally diagnosed as anxiety disorder due to another medical condition (293.84, Diagnostic and Statistical Manual of Mental Disorders-V). Patient did not fully respond to exercise direction, thus could be discharged with cognitive behavioral therapy by sharing biological and psychological problems to reduce low goal ownership, which is reported to predict drop-out from a weight intervention study in overweight patients with type 2 diabetes 2).

Discussion: Obesity has known to be spread through social tie 3). Resting-state functional connectivity in brain regions involved in cognitive control, motivation, and reward is reported to be enhanced in obese females 4). Brain function in this patient should be abnormal because plasma glucose ≥11 mmol/L has been reported to be associated with impaired activation of the DMN 1). Regional cerebral hemodynamic response to incremental exercise is blunted in poorly controlled patients with uncomplicated diabetes 5), as implied in clinical course of this patient. The evidence of usefulness in cognitive behavioral therapy is still weak in anxiety disorder 6).

Conclusions: Mental disorders due to obesity and/or diabetes should be treated earlier to lower HbA1c and improve insulin resistance.

References

1) Marder TJ, *Diabetes* 2014;63:3112–9
2) Huisman S, *Int J Behav Med* 2010;17:176–81
3) Christakis NA, *N Engl J Med* 2007;357:370–9
4) Lips MA, *Am J Clin Nutrition* 2014;100:524–32

© 2016 The Authors. Journal of Diabetes Investigation published by Asian Association for the Study of Diabetes (AASD) and John Wiley & Sons Australia, Ltd
This is an open access article under the terms of the Creative Commons Attribution License, which permits use, distribution and reproduction in any medium, provided the original work is properly cited.

5) Benett S, *BMJ* 2015;100:308–16
6) Tagougui S, *Diabetes Care* 2015;38:858–67.

### 0004

### Urinary liver-type fatty acid binding protein (L-FABP) in diabetic kidney disease in type 2 diabetes patients: a cross-sectional survey at diabetes specialist clinics in Japan

Microvascular complications

Y. Watanabe[1], M. Nogawa[2], H. Fujii[1] and T. Miyakawa[1,2]
[1]*Tama-centre Mirai Clinic, Tama, Tokyo, Japan,* [2]*Kunitachi Mirai Clinic, Kunitachi, Tokyo, Japan*

OBJECTIVES: Urinary liver-type fatty acid binding protein (L-FABP) is a new biomarker for diabetic kidney disease (DKD) by detecting oxidative stress at proximal convoluted tubules. We conducted a cross-sectional survey to reveal relationship between urinary L-FABP and albuminuria and an estimated glomerular filtration rate (eGFR).

METHODS: A total of 763 consecutive adult type 2 diabetic patients seen at two diabetes clinics in Tokyo were enrolled. Eligible patients had spot urine albumin to creatinine ratio (ACR) measured at least two times per year by immunoturbidimetric assay. An eGFR was calculated by the 3-variable Japanese equation. Urinary L-FABP was measured once by ELISA, and the reference value was <8.5 μg/gCr. We excluded patients with eGFR <15.

RESULTS: Patients had a mean age (SD), 62.1 (12.7); male, 64%; mean HbA1c, 7.17% (1.17); ACR 105.7 mg/g creatinine (395.1); eGFR 58.8 (15.9). Albuminuria was categorized in three strata (% of the patients): normoalbuminuria, A1 (70.4%); microalbuminuria, A2 (22.7%) and macroalbuminuria, A3 (6.9%). Estimated GFR was divided into five groups according to KDIGO's CKD stage definition and % of the patients were as follows: G1, 2.8%; G2, 39.8%; G3a, 37.6%; G3b, 18.6%; and G4, 1.2%. Mean urinary L-FABP in A1, A2 and A3 was 5.3, 9.9 and 23.5, respectively ($P < 0.001$). Whereas mean urinary L-FABP in G1, G2, and G3a was 6.5, 6.6, and 6.8, respectively and those were less than the reference value; mean urinary L-FABP in G3b and G4 was 11.4 and 8.9, respectively ($P < 0.0001$). We then divided ACR categories in two at eGFR of 45, lower eGFR showed significantly high urinary L-FABP in A2 and A3 ($P < 0.001$). Statin use showed no difference in ACR categories. However, ARB use was high in A3 ($P < 0.001$).

CONCLUSIONS: Urinary L-FABP highly correlated with ACR and eGFR in detecting DKD. In A2 and A3 patients, urinary L-FABP correlated more to eGFR <45. Prospective study is needed to evaluate its precision for detecting early DKD.

### 0005

### Associations of serum adipocyte fatty acid binding protein with body composition and fat distribution in nondiabetic Chinese women

Obesity

Y. Bao[1], X. Ma[1], Y. Hao[1], Y. Luo[1], X. Hu[1], X. Pan[1], Y. Xiao[2] and W. Jia[1]
[1]*Department of Endocrinology and Metabolism, Shanghai Jiao Tong University Affiliated Sixth People's Hospital; Shanghai Clinical Center for Diabetes; Shanghai Key Clinical Center for Metabolic Disease, Shanghai, China,* [2]*Department of Radiology, Shanghai Jiao Tong University Affiliated Sixth People's Hospital, Shanghai, China*

OBJECTIVE: Previous studies have demonstrated evidence of a positive relationship between serum adipocyte fatty acid binding protein (A-FABP) and obesity. However, associations of A-FABP with body composition and ectopic fat accumulation remain unclear. The study aimed to elucidate the impact of body composition, visceral fat area (VFA), and subcutaneous fat area (SFA) on serum A-FABP levels in a cohort of Chinese women without diabetes mellitus.

METHODS: A total of 2108 women without diabetes (760 premenopausal and 1348 postmenopausal women; age, 20–78 years) selected from the Shanghai Obesity Study were enrolled. VFA and SFA were measured by magnetic resonance imaging, and body composition was determined by bioelectrical impedance analysis. A high VFA was defined as ≥80 $cm^2$. A high SFA was defined as that above the 75th percentile cutoff point of the menopause-specific population, respectively.

RESULTS: Serum A-FABP levels were higher in postmenopausal than premenopausal women ($P < 0.001$). Both premenopausal and postmenopausal women with an isolated high VFA had higher A-FABP levels than did those with an isolated high SFA ($P = 0.017$ and 0.002, respectively). In both body mass index (BMI) groups (<25 and ≥25 $kg/m^2$), women with a high VFA had higher serum A-FABP levels regardless of their menopausal status. Multiple stepwise regression analysis showed that A-FABP was independently associated with fat mass (Standardized $\beta = 0.417$ and 0.252 for premenopausal and postmenopausal status, respectively, both $P < 0.001$). Moreover, VFA was identified as an independent risk factor for A-FABP in postmenopausal women (Standardized $\beta = 0.114$, $P = 0.001$). Application of the same regression analyses model to the two BMI groups produced similar results in both BMI categories.

CONCLUSIONS: Serum A-FABP levels were associated with fat mass, and were also influenced by VFA after menopause in Chinese women without diabetes mellitus.

### 0006

### Association between serum osteocalcin level and visceral obesity in Chinese postmenopausal women

Obesity

Y. Luo[1], X. Ma[1], Y. Hao[1], Y. Xu[1], Q. Xiong[1], J. Tang[1], X. Pan[1], Y. Xiao[2], Y. Bao[1] and W. Jia[1]
[1]*Department of Endocrinology and Metabolism, Shanghai Jiao Tong University Affiliated Sixth People's Hospital; Shanghai Clinical Center for Diabetes; Shanghai Key Clinical Center for Metabolic Disease, Shanghai, China,* [2]*Department of Radiology, Shanghai Jiao Tong University Affiliated Sixth People's Hospital, Shanghai, China*

OBJECTIVES: A low serum osteocalcin level, visceral obesity, and a menopausal status are recognized risk factors for cardiovascular disease. We investigated the relationship between the serum osteocalcin level and visceral fat content in a population of Chinese postmenopausal women.

METHOD: In total, 1481 postmenopausal women (mean age ± standard deviation, 57.1 ± 4.8 years) were selected from the Shanghai Obesity Study. Abdominal fat accumulation was quantified using magnetic resonance imaging. Subjects with a visceral fat area (VFA) of ≥80 $cm^2$ were classified as abdominally obese. The total serum osteocalcin level was measured by electrochemiluminescence immunoassay.

RESULTS: The median serum osteocalcin level was 20.66 ng/mL (interquartile range, 16.88–25.42 ng/mL). The overall prevalence of abdominal obesity was 49.1% ($n = 727$). Abdominally obese subjects had lower serum osteocalcin levels than did non-abdominally obese subjects (19.14 [16.02–23.82] versus 21.97 [18.14–26.77] ng/mL, respectively; $P < 0.001$). Partial correlation analysis showed that the serum osteocalcin level was still negatively correlated with the VFA after adjusting for age, years since menopause and body mass index ($P < 0.01$). Moreover, VFA was independently associated with the serum osteocalcin level after adjustment for confounding factors ($P < 0.05$). A low serum osteocalcin level was an independent risk factor for abdominal obesity (odds ratio, 0.972; 95% confidence interval, 0.953–0.991; $P = 0.004$).

CONCLUSIONS: The serum osteocalcin level was inversely correlated with the visceral fat content in these Chinese postmenopausal women.

### 0008

### Prevalence of hyperuricemia in patients with metabolic syndrome

Prediabetes and Metabolic Syndrome

C. Clado-Reyes, A. Lim-Teodoro, E. Salimo and G. Pura
*World Citi Medical Center, Quezon City, The Philippines*

BACKGROUND: Studies have showed that the prevalence of coronary heart disease, myocardial infarction and stroke are higher in subjects with the metabolic syndrome and hyperuricemia than it was in those without them

© 2016 The Authors. Journal of Diabetes Investigation published by Asian Association for the Study of Diabetes (AASD) and John Wiley & Sons Australia, Ltd

OBJECTIVE: The aim of the study is to determine the prevalence and relationship of hyperuricemia with metabolic syndrome and its components.

METHODS: This is a cross sectional study of patients aged 18 years old and above, admitted in a tertiary medical center from November 2013 to October 2014, with metabolic syndrome. Metabolic syndrome was defined using National Cholesterol Education Program Expert Panel on Detection, Evaluation, and Treatment of High Blood Cholesterol in Adults (Adult Treatment Panel III). Hyperuricemia is defined as >7 mg/dL (416 μmol/L) in men and >6 mg/dL (357 μmol/L) in women. Anthropometric, clinical and biochemical data were gathered.

RESULTS: One hundred twelve patients with metabolic syndrome were included in the study. The prevalence of hyperuricemia in patients with metabolic syndrome was 47.3% (53/112), with slightly female preponderance than in males (54% vs. 45%). Significant association was noted between diabetes mellitus and hyperuricemia, but of negative association ($P = 0.01$). Abdominal obesity has the highest prevalence of hyperuricemia reaching 92%. and significantly correlated with hyperuricemia ($P$ value 0.03). There was no significant association noted in hypertension, Triglyceride and HDL with hyperuricemia.

CONCLUSION: The prevalence of hyperuricemia in individuals with metabolic syndrome was 47.3%. Among patients with metabolic syndrome, diabetes mellitus was negatively correlated with hyperuricemia, albeit a weak correlation. Abdominal obesity has the highest prevalence and significantly correlated with hyperuricemia. There was no correlation noted on the other components of metabolic syndrome. The growing incidence of hyperuricemia and metabolic syndrome is correlated to an increase risk for developing heart attack and stroke. Therefore, control of hyperuricemia and metabolic syndrome must be addressed to prevent the potential risk for developing coronary heart diseases and stroke.

### 0009

### A variant of PSMD6 is associated with the therapeutic efficacy of oral antidiabetic drugs in Chinese type 2 diabetes patients

Genetics and Epigenetics

M. Chen, C. Hu, R. Zhang and W. Jia

*Shanghai Diabetes Institute, Department of Endocrinology and Metabolism, Shanghai Clinical Center for Diabetes, Shanghai Key Clinical Center for Metabolic Disease, Shanghai Key Laboratory of Diabetes, Shanghai, China*

OBEJECTIVES: The *PSMD6* variant rs831571 has been identified as a susceptibility locus for type 2 diabetes mellitus (T2DM). This study aimed to investigate the association of this variant with therapeutic effects of oral antidiabetic drugs in Chinese T2DM patients.

METHOD: 209 newly diagnosed T2DM patients were randomly assigned to treatment with repaglinide or rosiglitazone for 48 weeks, and the therapeutic effects were compared.

RESULTS: In the rosiglitazone cohort, rs831571 showed significant associations with fasting plasma glucose (FPG), 2-h glucose and decrement of glycated haemoglobin (HbA1c) levels after 24 weeks of treatment ($P = 0.0368$, 0.0468 and 0.0247, respectively). The C allele was significantly associated with reduced FPG at 24 and 32 weeks ($P = 0.0172$ and 0.0257, respectively). Survival analyses showed CC homozygotes were more likely to attain a standard FPG level ($P = 0.0654$). In the repaglinide cohort, rs831571 was significantly associated with decreased HbA1c levels after 24 weeks of treatment, the homeostatic model assessment of insulin resistance and fasting insulin level after 48 weeks of treatment with repaglinide ($P = 0.0096$, 0235 and 0.0212, respectively).

CONCLUSIONS: In conclusion, we observed that the *PSMD6* variant rs831571 might be associated with the therapeutic effects of rosiglitazone and repaglinide in Chinese T2DM patients. However, these findings need to be confirmed in the future.

### 0013

### Association between serum uric acid related genetic loci and diabetic kidney disease in the Chinese type 2 diabetes patients

Microvascular Complications

D. Yan, J. Wang, C. Hu and W. Jia

*Shanghai Diabetes Institute, Shanghai Key Laboratory of Diabetes Mellitus, Shanghai Clinical Center for Diabetes, Shanghai Jiao Tong University Affiliated Sixth People's Hospital, Shanghai, China*

OBJECTIVES: Uric acid has been identified to be associated with diabetic kidney disease (DKD) in previous studies, but results of these studies are inconsistent, and whether uric acid related genes impact on DKD remains unclear, we aimed to investigate the association between uric acid related genetic loci and DKD susceptibility in type 2 diabetes patients.

METHODS: Seventeen single nucleotide polymorphisms (SNPs) from thirteen loci related to serum uric acid were genotyped in 2,892 type 2 diabetes patients. The basic characteristics of the patients were recorded, and patients with DKD were included in the case group. Patients with diabetes for more than 5 years and normal kidney function were categorized as controls. The associations between SNPs and uric acid level, SNPs and quantitative traits related to DKD or DKD susceptibility were evaluated.

RESULTS: In this study, uric acid showed a strong association with oDKD (OR 1.006(1.005–1.007), $P < 0.0001$). *GCKR* rs780094, *SLC2A9* rs11722228, *SLC2A9* rs3775948, *ABCG2* rs2231142, *SLC22A12* rs505802 and *NRXN2* rs506338 were positively associated with serum uric acid ($P = 0.0005$, 0.0008, 5.57E-07, 2.51E-06, 0.0018, and 0.0015; empirical $P = 0.0071$, 0.0113, 1.00E-04, 1.00E-04, 0.0264, and 0.0222; respectively). *SLC2A9* rs11722228 and *SF1* rs606458 were significantly associated with DKD susceptibility (OR 0.864(0.749–0.996), $P = 0.0440$; OR 1.223(1.067–1.404), $P = 0.0038$), *SLC2A9* rs3775948 and *ABCG2* rs2231142 were associated with DKD marginally (OR 0.878(0.770–1.000), $P = 0.0506$; OR 0.879(0.764–1.010), $P = 0.0698$). *SLC2A9* rs11722228, *SLC2A9* rs3775948, *ABCG2* rs2231142 and *SF1* rs606458 were significantly associated with the estimated glomerular filtration rate (eGFR) ($P = 0.0005$, 0.0006, 0.0003, 0.0424, respectively).

CONCLUSIONS: Our study indicated that the uric acid related alleles of *SLC2A9* rs11722228, *SLC2A9* rs3775948, *ABCG2* rs2231142 were associated with DKD susceptibility in the Chinese type 2 diabetes patients.

### 0016

### Rosuvastatin decreasing testosterone levels but not sexual function in patients with type 2 diabetes

Lipid Disorders

C. J. Hsieh

*Division of Endocrinology and Metabolism, Department of Internal Medicine, Kaohsiung Chang Gung Memorial Hospital and Chang Gung University College of Medicine., Kaohsiung, Taiwan*

Autonomic neuropathy and hypogonadism are the cause of impotence in men with diabetes. Hypercholesterolemia is also one of the dominant comorbidity of diabetes. Many men with diabetes are being treated with statins to low down the serum levels of cholesterol. However, cholesterol is the major substrate for testosterone biosynthesis, statin may decrease serum testosterone levels via decreasing cholesterol level. In previous studies, few longitudinal studies with few enrolled subject were done before and with conflict results. Free testosterone is also not routinely used, so compromised the accuracy. We analyze the effect of statins on free testosterone levels in a longitudinal study of men with type 2 diabetes.

METHODS: We enrolled 51 men with type 2 diabetes and hypercholesteroemia. Clinical and biochemical assessments include body height, body weight, blood pressure, fasting and post-prandial blood glucoselevels glycated hemoglobin (HbA1c), serum total cholesterol (TC), low-density lipoprotein (LDL), high-density lipoprotein (HDL), triglyceride (TG), prolactin, Thyroid-stimulating hormone (TSH) luteinizing hormone (LH), follicle stimulating hormone (FSH) and free testosterone, which were measured before statin treatment, after 3 months of statin treatment and then after quitted statin treatment for 3 months, and also gave questionnaire about sex function (SHIM).

© 2016 The Authors. Journal of Diabetes Investigation published by
Asian Association for the Study of Diabetes (AASD) and John Wiley & Sons Australia, Ltd

RESULTS: Free testosterone was also lower after statin therapy, 22.4 ± 3.1 ng/mL before statin therapy, 20.9 ± 2.8 ng/mL after 3 months of statin therapy and 22.6 ± 2.6 ng/mL after quitted statin therapy for 3 months (*P* value = 0.006). However, the SHIM score was not apparent difference between 3 stages (16.3 ± 4.8 vs. 16.0 ± 4.9 vs. 16.3 ± 5.0 *P* = 0.944). The serum free testosterone levels were significantly SHIM score (r = 0.38, *P* = <0.001), moderately correlated with LDL (r = 0.25, *P* = 0.04), but not total cholesterol (r = 0.13, *P* = 0.10).

CONCLUSION: Using statin may decrease the serum free testosterone levels of male patients with type 2 diabetes. The mechanism is due to cholesterol is the precursor of testosterone production and statin decreases it. However, statin could decrease atherosclerosis in patients with type 2 diabetes. This effect also could increase circulation of sex organ and prevent diabetic neuropathy induced erection dysfunction and impotence. This effect compliments the side effect of statin related hypoandrogenism.

### 0020

### Association of genetic variants in *LYPLAL1* with visceral fat and subcutaneous fat distribution in Chinese population

Obesity

T. Wang, R. Zhang, F. Jiang, C. Hu and W. Jia

*Shanghai Diabetes Institute, Shanghai Key Laboratory of Diabetes Mellitus, Shanghai Clinical Center for Diabetes, Shanghai Jiao Tong University Affiliated Sixth People's Hospital, Shanghai, China*

OBJECTIVES: Numerous genetic variants associated with simple but less precise anthropometric measurements of obesity including body mass index (BMI), waist circumference, waist to hip ratio have been uncovered using genome-wide association studies (GWAS). However, association studies of the genetic architecture of visceral and subcutaneous fat distribution still remain unknown. We test if the precise visceral and subcutaneous fat distribution indices could provide important information beyond BMI, waist circumference, and waist-to-hip ratio with respect to identify causal variants.

METHOD: A total of 2958 subjects from a community-based population with Chinese Han ancestry were genotyped for several single-nucleotide polymorphisms (SNPs), who underwent Magnetic Resonance Imaging (MRI) for measurement of visceral fat area (VFA) and subcutaneous fat area (SFA).

RESULTS: The association of *SEC16B*, *BDNF*, *MC4R* and *LYPLAL1* with BMI, waist circumference and waist to hip ratio were validated in Chinese populations ($P = 9.7 \times 10^{-7}$~0.0471). In search for fat distribution variants, rs4846567 at *LYPLAL1* showed a nominal association with SFA and the VFA-SFA ratio with or without the adjustment of BMI (*P* = 0.0091~0.0325). Subgroup analysis stratified by sex indicated the significant associations of rs4846567 at *LYPLAL1* with SFA and the VFA-SFA ratio in women only (*P* = 0.0144 and 0.0018, respectively), which even exhibited marginally significant after multiple testing (empirical *P* = 0.0778).

CONCLUSIONS: Our results suggest that the link between *LYPLAL1* and subcutaneous fat distribution may be independent of overall adiposity and exert influence in a sex-dependent manner.

KEY WORDS: Single-nucleotide polymorphisms, Fat distribution, Chinese, *LYPLAL1*.

### 0021

### Once daily insulin degludec injection has achieved non-inferior glycemic control with less dosege among intensive insulin therapy of type 1 diabetes patients who are splitting basal insulin twice in Japan

Therapeutics of Diabetes

H. Fujii[1], S. Ito[2], K. Kanno[3], M. Kato[4], N. Kato[4], H. Kondo[5], N. Yoshida[5], M. Morita[5], R. Sato[6], H. Takamura[7], A. Ueki[7], M. Takesue[8], E. Kodani[9], H. Daikoku[10], M. Nogawa[11], Y. Watanabe[1], Y. Kawagoe[1], T. Miyakawa[11] and T. Kitaoka[12]

[1]*Tama-center Mirai Clinic, Tama-city, Japan,* [2]*Ito Clinic, Fuchu-city, Japan,* [3]*Kanno Clinic, Mitaka-city, Japan,* [4]*Kato Clinic, Katsushika-ku, Japan,* [5]*Kondo Clinic, Kodaira-city, Japan,* [6]*Kunitachi-naika Clinic, Kunitachi-city, Japan,* [7]*Takamura Clinic, Fussa-city, Japan,* [8]*Takesue Pediatric Clinic, Mitaka-city, Japan,* [9]*Department of Internal Medicine and Cardiology Nippon Medical School > Tama-Nagayama Hospital, Tama-city, Japan,* [10]*Showa General Hospital, Kodaira-city, Japan,* [11]*Kunitachi Mirai Clinic, Kunitachi-city, Japan,* [12]*NPO West-Tokyo Diabetes Association, Kokubunji-city, Japan*

Japanese type 1 diabetes was considered to require less basal insulin because of insulin sensitivity and carbohydrate consumption. For better control and prevention of hypoglycemia, splitting basal injection to twice a day has been common.

TRAIN 2 is a multicenter observational study which aimed to switch basal insulin to new basal insulin analog, Degludec in intensive insulin therapy, approved by IRB of the West Tokyo Diabetes Association. The participants were followed for 6 months. Among 131, we analyzed 80 type 1 patients (excluding 3: used NPH, and 1: newly diagnosed).

By previous basal and splitting, we divided them into 4; Glargine once (*n* = 16), twice (29), Detemir once (8), and twice (27). Age, sex, duration of illness /insulin injection, HbA1c, BMI, and C peptide were not different. HbA1c did not improve nor worsen after 6 months. Among groups, it was not different throughout the study. Total dose decreased from 40.4 to 37.6 U/d. It was different from 31.8 (once Detemir) to 42.9U/d (twice Detemir) at start but not after 6 months.

Bolus dose did not change in total, however in Detemir twice, it increased before dinner (+1.2U), and in Glargine twice, it decreased in the morning by 0.8U.

Basal dose decreased from 17.8 to 14.9 U/d. At start; once Detemir basal rate (30.9%) was the lowest, twice Glargine (43.6%) and twice Detemir (47.1%) was higher. This tendency was same after 6 months; once Detemir (27.5%), twice Glargine (39.2%), and twice Detemir (43.2%).

The frequent hypoglycemia more than once a week decreased in both daytime (55–36%) and nighttime (16–11%).

Replacing basal insulin to once daily Degludec has achieved noninferior glycemic control with decreased basal dose. Degludec acts longer and peak less to satisfy insulin requirement, as well as eliminate the waste of insulin piling up by splitting injection. Shortacting insulin dose adjustment was required because of its flatness.

### 0022

### Dietary habits of type 1 diabetes patients have become apparent by ambulatory continues glucose monitoring (professional CGM)

Behaviour and Nutrition

M. Kunisada[1], H. Fujii[1], Y. Watanabe[1], Y. Kawagoe[1], M. Nogawa[2] and T. Miyakawa[2]

[1]*Tama-center Mirai Clinic, Tama-city, Japan,* [2]*Kunitachi Mirai Clinic, Kunitachi-city, Japan*

We have been using ambulatory CGM (professional use) for 5 years in Japan. It is useful to adjust insulin to make "the glucose curve" more stable and flatter. We also realized that even non-obese type 1 patients needed dietary advice to build stable and healthy diet habits. The reports of "National Nutrition Survey" by the Ministry of Health, Labor and Welfare pointed out that;

1) Skipping breakfast rate has increased (male: 12.8%, female: 9.0% in 2013 report). Especially in 20s; male: 30.0%, female: 26.2%.

2) Dinner starts later; more than 7% of male in their 30s and 40s ate dinner later than 11 PM (2006).

3) The consumption of vegetables and fruits was lowest in the 20s (only 60% of the recommended daily amount), and highest in the 60s and the 70s.

© 2016 The Authors. Journal of Diabetes Investigation published by Asian Association for the Study of Diabetes (AASD) and John Wiley & Sons Australia, Ltd

1 week-CGM with diet reports was performed by total 77 sets for 55 type1 patients in a private diabetes clinic in Tokyo. One dietitian who reviewed the data, gave them dietary advice.

35 of them were male, age, 45.5 ± 15.2 y/o, HbA1c, 7.4 ± 1.1%. Multiple injections, 47, CSII, 8.

Advices by the dietitian were; about carbo-counting, balancing nutrition, unfavorable dietary habits, and so on.

All of the patients age >65, prepared more than 3 kinds of vegetables for each meal. Patients≦65 tended to take mainly carbohydrates for breakfast, and this was considered to be a disadvantage to lower postprandial glucose in the morning. Skipping breakfast was observed in 3. Male workers ate late dinner. Female in their 20s and 30s tended to have unbalanced food such as eating snacks as a meal, and put emphasis only on dinner with family. One male teenager ate only once a day.

We used to focus on how to technically adjust insulin to their lifestyle and their glycemic curve for especially lean type 1 diabetes patients without any metabolic risks other than glycemic control. However there would be future problems such as metabolic syndrome, osteoporosis, and low birth weight babies. We explained their CGM curve according to their dietary patterns. CGM has become a new tool of diet therapy even for type 1 diabetes.

### 0023

### A survey using a self-answered questionnaire about patients' satisfaction taking a new-class medicine, SGLT2 inhibitors

Education and Care Delivery

Y. Asada[1], C. Takayama[1], T. Mori[2], A. Nonaka[3], H. Fujii[4], Y. Watanabe[4], Y. Kawagoe[4], M. Nogawa[5] and T. Miyakawa[5]

[1]*Uran Pharmacy, Tama-city, Japan,* [2]*Kunitachi Uran Pharmacy, Kunitachi-city, Japan,* [3]*Yamato Prescription Center, Higashiyamato-city, Japan,* [4]*Tama-center Mirai Clinic, Tama-city, Japan,* [5]*Kunitachi Mirai Clinic, Kunitachi-city, Japan*

From April 2014, a new class medicine, Sodium-glucose cotransporter 2 inhibitors; such as ipragliflozin, dapagliflozin, luseoglifrozin, tofoglifrozin, canaglifrozin, and empaglifrozin have been approved for use in Japan. It is expected to reduce body weight by inhibiting glucose reabsorption and improve glycemic control at the same time. However it should be carefully used by observing dehydration and urogenital infections. Also relative, many skin reactions have been reported in the short period.

We administered a self-answered questionnaire three times (at the start, 2-weeks later, and 3 months later) to the 31 diabetes patients who started to be prescribed SGLT2 Inhibitors from April till September 2014.

The average age of the patients was 52.2 y/o (SD: 7.9), duration of illness; 10.7 (7.8) yrs, male 22, female 9. HbA1c at the start; 8.1%, it reduced to 7.6% after 3 months ($P < 0.05$; *t*-test).

Question 1. 'Satisfaction over all' was improved significantly after 3 months.

Question 2. 'Satisfaction about your body weight' also significantly improved after 3 months.

Question 3. In 2 weeks, 12 Patients lost more than 2 kg, although they did not lose weight anymore, however their body weight was still significantly lower after 3 months comparing to the start.

Question 4 and 5. Change of appetite, especially for sweets did not increase significantly.

Question 6,7. 27 out of 31 increased the amount of water they drank, 500~2000 mL per day.

Question 8. Hypoglycemia was experienced by 4 patients; 2 of them were using insulin.

Question 9~11. 87.5% answered that the amount of urine and the frequency of urination.

Question 12 However only 19.4% answered it was durable.

Question 13 2 females experienced urogenital infection.

Question 14. Evaluation overall was 74.4 (at start), 80.8 (2 weeks), 76.1 (3 months) out of 100.

Narratively, patients sometimes bargained urogenital infection or frequency of urination to losing weight or improvement of glycemic control.

In conclusion, this drug may have a narrow window to select the best patients by considering its risks and benefits especially for Japanese. For patient's Quality of Life, pharmacists have some keys by listening to the patients carefully and making a better atmosphere to find out the facts.

### 0024

### Association of obesity genetic variants in *NPC1* with Type 2 diabetes in a Chinese population

Genetics and Epigenetics

Z. He, R. Zhang, F. Jiang, C. Hu and W. Jia

*Shanghai Diabetes Institute, Shanghai Key Laboratory of Diabetes Mellitus, Shanghai Clinical Center for Diabetes, Shanghai Jiao Tong University Affiliated Sixth People's Hospital, shanghai, China*

OBJECTIVES: The overweight and obesity are major risk factors of type 2 diabetes mellitus (T2DM). However, for most of the recent uncovered obesity-associated loci including *NPC1* variants, their role in impacting on type 2 diabetes remains elusive.

METHODS: A total of 6822 case–control participants of Han Chinese ancestry (3410 T2DM patients vs 3412 normal glucose regulation subjects) underwent a detailed clinical investigation and were genotyped for several single-nucleotide polymorphisms (SNPs) associated with obesity. Logistic and linear regression analyses were performed to determine the association with type 2 diabetes risk or related glycaemic traits, respectively.

RESULTS: The SNPs in *SEC16B*, *CDKAL1*, *FTO* and *MC4R* were associated with type 2 diabetes risk without adjustment for BMI ($P = 5.03 \times 10^{-10}$~0.0022), and the association was slightly attenuated after adjustment for BMI ($P = 4.58 \times 10^{-11}$~0.0079), whereas rs1805081 in *NPC1* was found no association with T2DM. In non-diabetic controls, rs1805081 was associated with fasting insulin levels as well as insulin secretion and sensitivity indices ($P = 0.0042$~0.0267), the association was decreased after adjustment for age, sex, BMI and waist circumference ($P = 0.0051$~0.0352). Further analysis showed that the insulin-decreasing allele also showed borderline association to higher LDL cholesterol ($P = 0.0575$).

CONCLUSION: Our results suggested that predisposition of genetic variants in *NPC1* to obesity were linked to glucose metabolism traits, independent of BMI and partly through impaired beta cell function.

### 0025

### Effect of one administration of glinide (mitiglinide 10 mg) with dipeptidyl peptidase-4 inhibitor in Type 2 diabetes mellitus

Clinical Diabetes

K. Takeda, T. Tatsumi, T. Yagi, M. Ishida, K. Nisimura, Y. Miyazaki and Y. Takanashi

*Kyoto Industrial Health Association, Kyoto, Kyoto, Japan*

PURPOSE: Recently, dipeptidyl peptidase-4 inhibitor (DPP-4i) is first choice drug for the treatment of DM. However, many times, only use of DPP-4i does not control HbA1c. What is Second choice is problem. We used glinide (mitiglinide 10 mg) only one time before supper. DPP-4i and glinide dose not occur hypoglycemic attack.

MATERIAL AND METHODS: 15 type 2 diabetes mellitus (T2DM) patients were administrated with DPP-4i (Vildagliptin 6 and Linagliptin 9). In these patients, mean age was 65,4 ± 3,3 (mean ± SE) years old. Then, Mitiglinide was administrated only once10 mg only just before Supper. 2 month later, HbA1c was checked.

RESULTS: Before administration of mitiglinide, HbA1c was 6.97% ± 0.09, Blood glucose (BG)157.7 ± 11.5 mg/dL, Systolic blood pressure(SBP) 146.4 ± 3.7 mmHg, diastolic blood pressure(DBP) 92.4 ± 2.0 mmHg, eGFR 58.9 ± 5.2 mL/min, LDL 70.7 ± 5.9 mg/dL, HDL 52.4 ± 0.0 mg/dL, TG 141.4 ± 16.2 mg/dL. After administration of mitiglinide 10 mg, HbA1C was 6.70% ± 0.09 ($P < 0.01$), BG 150.7 ± 11.5 (N.S.), SBP 152.2 ± 4.2 (N.S.), DBP 92.4 ± 1.8 (N.S.), eGFR 59.4 ± 5.8 (N.S.) LDL 70.7 ± 5.9 (N.S.) HDL 52.4 ± 3.8 (N.S), TG 141.4 ± 16.2 (N.S.).

CONCLUSION: These findings show one dose of mitiglinide with DPP-4i was effective to lower HbA1c in T2DM.

© 2016 The Authors. Journal of Diabetes Investigation published by Asian Association for the Study of Diabetes (AASD) and John Wiley & Sons Australia, Ltd

### 0026

### Inverse association of serum vitamin D in relation to carotid intima-media thickness in Chinese postmenopausal women

Macrovascular Complications

X. Ma[1], Y. Hao[1], Y. Luo[1], Y. Xu[1], Q. Xiong[1], J. Zhu[2], Y. Bao[1] and W. Jia[1]
[1]*Department of Endocrinology and Metabolism, Shanghai Jiao Tong University Affiliated Sixth People's Hospital; Shanghai Clinical Center for Diabetes; Shanghai Key Clinical Center for Metabolic Disease, Shanghai, China,* [2]*Department of Ultrasound, Shanghai Jiao Tong University Affiliated Sixth People's Hospital; Shanghai Institute of Ultrasound in Medicine, Shanghai, China*

Objective: Perturbed vitamin D status may contribute to obesity, metabolic syndrome and cardiovascular disease. The incidence and risks of inadequate vitamin D in postmenopausal women have recently come to the forefront of clinicians' attention worldwide. This study aimed to investigate the relationship between serum vitamin D level and carotid intima-media thickness (C-IMT, an indicator of sub clinical atherosclerosis) in Chinese postmenopausal women.

Methods: Nine hundred and twenty six Chinese postmenopausal women without carotid artery plaque or history of cardiovascular disease were selected for analysis. Measurements of serum 25 hydroxyvitamin D3 (25(OH)D3) concentration and C-IMT were made by electrochemiluminescence immunoassay and B-mode ultrasound, respectively.

Results: The median serum 25(OH)D3 level was 11.03 ng/mL, with an interquartile range of 8.22–14.70. A decreasing trend of serum 25(OH)D3 level was accompanied by increased C-IMT tertiles ($P$ for trend = 0.001). Correlation analysis found an inverse relationship between serum 25(OH)D3 level and C-IMT ($r = -0.113$, $P = 0.001$). After adjustment for confounding factors, multiple stepwise regression analysis showed that serum 25(OH)D3 level independently and negatively associated with C-IMT (Standard $\beta = -0.112$, $P < 0.001$). Moreover, the inverse correlation of serum 25(OH)D3 with C-IMT was also found in a subgroup of women with normal glucose tolerance, blood pressure and body mass index, and without undergoing lipid-lowering therapy (standard $\beta = -0.140$, $P = 0.018$).

Conclusions: Serum 25(OH)D3 level was inversely correlated with C-IMT in Chinese postmenopausal women.

### 0027

### Serum betatrophin concentrations are significantly increased in overweight but not in obese or Type 2 diabetic individuals

Obesity

K. Guo, J. Lu, H. Yu, F. Zhao, P. pan, L. Zhang, Y. Bao, H. Chen and W. Jia
*Shanghai Jiaotong University Affiliated Sixth People's Hospital, Shanghai, China*

Objective: In this study we quantitated circulating serum betatrophin levels and examined their relationship with insulin resistance (IR) and other metabolic parameters in Chinese subjects with varying degrees of obesity and glucose tolerance.

Design and Methods: Serum betatrophin levels were determined using ELISA in 60 subjects with normal glucose tolerance (NGT; 17 lean, 23 overweight and 20 obese subjects) and 56 subjects with type 2 diabetes mellitus (T2DM; 14 lean, 23 overweight and 19 obese subjects). The association of serum betatrophin levels with adiposity, glucose, lipid profile and hepatic enzyme parameters was studied.

Results: Serum betatrophin concentrations were significantly higher in overweight subjects in both NGT and T2DM groups, however, no significant difference between lean and obese participants was observed. No significant difference was found between males and females and between NGT and T2DM subjects. Serum betatrophin concentrations correlated positively with fasting insulin, homeostasis model assessment-estimated insulin resistance (HOMA-IR), γ-glutamyl transpeptidase(γ-GT) and alanine aminotransferase (ALT) in all subjects. Serum betatrophin concentrations showed an independent association withγ-GT and HOMA-IR.

Conclusions: Serum betatrophin is significantly increased in overweight subjects in both NGT and T2DM groups, however, no significant difference between lean and obese participants was observed. Serum betatrophin concentrations were significantly associated with IR, but not with lipid profiles, glucose homeostasis or diabetes.

### 0028

### Effects glucose and fructose feeding on cardio-metabolic risk factors: role of non-selective inhibition of sodium-glucose co-transporter

Prediabetes and Metabolic Syndrome

H. Poudyal, H. Norio and N. Inagaki
*Kyoto University, Kyoto, Japan*

Objective: To compare the effects of glucose and fructose feeding on body composition and changes in cardiac, hepatic and pancreatic structure/ function. Additionally, we have evaluated the effects of non-selective inhibition of sodium-glucose co-transporter (SGLT) in both glucose and fructose fed rats.

Method: Male Wistar rats were fed isocaloric diets with 60% carbohydrates exclusively as glucose or fructose for 4 weeks. Changes in body composition (CT Scan), cardiovascular (isolated heart preparation, echocardiography, histochemistry, blood pressure), hepatic (histochemistry, plasma markers) and pancreatic (histochemistry) structure/and function were measured. Additionally, we measured changes in glucose, insulin, GLP-1 and GIP during an oral glucose tolerance test.

Results: Glucose or fructose feeding did not affect body weight. However, glucose but not fructose feeding increased total subcutaneous and visceral fat. Phlorizin supplementation decreased body weight in both groups by specifically decreasing visceral fat.

Glucose and fructose feeding for 4 weeks had no effects on oral glucose tolerance. Phlorizin supplementation resulted in lower peak 60 min blood glucose concentration in both groups compared to chow fed rats and therefore reduced area under the curve. No major changes in GIP concentrations were observed in glucose and fructose fed rats. However, phlorizin supplementation resulted in lower peak 60 min plasma GIP concentration in both groups which also showed elevated fasting plasma GIP concentrations. No significant differences in insulin and GLP-1 concentration or AUC was observed amongst groups.

Blood pressure was significantly higher in fructose but not glucose-fed rats and was normalised by phlorizin. Glucose and fructose supplementation induced increase in left ventricle internal dimension in diastole but not in systole without changing posterior wall dimension suggesting eccentric hypertrophy. Additionally, fructose but not glucose increased myocardial stiffness most likely by increased deposition of interstitial collagen. Phlorizin supplementation normalised LV dilatation in both glucose and fructose fed groups but did not significantly alter volumetric indices. Ex vivo experiments using isolated heart preparation showed selective improvement in LV contractility in glucose fed rats supplemented with phlorizin with no statistically significant changes in other groups.

Conclusion: These results indicate that glucose has similar effects on most cardio-metabolic risk factors as compared to fructose with the exception of adiposity where glucose was more detrimental and blood pressure/ LV structure where fructose induced greater damage. The fact that SGLT inhibition reversed most of the glucose or fructose induced damage may have clinical implications.

© 2016 The Authors. Journal of Diabetes Investigation published by Asian Association for the Study of Diabetes (AASD) and John Wiley & Sons Australia, Ltd

### 0032

### Glargine insulin/gliclazide MR combination therapy is more effective than premixed insulin monotherapy in Chinese patients with type 2 diabetes inadequately controlled on oral antidiabetic drugs

Therapeutics of diabetes

J. Zhou[1], F. Zheng[2], X. Guo[3], H. Yang[4], M. Zhang[5], H. Tian[6], L. Guo[7], Q. Li[8], Y. Mo[1] and W. Jia[1]

[1]*Department of Endocrinology and Metabolism, Shanghai Jiao Tong University Affiliated Sixth People's Hospital, Shanghai Clinical Center for Diabetes, Shanghai Diabetes Institute, Shanghai Key Laborator, Shanghai, China,* [2]*Department of Endocrinology and Metabolism, Sir Run Run Shaw Hospital, School of Medicine, Zhejiang University, Hangzhou, China,* [3]*Department of Endocrinology and Metabolism, Peking University First Hospital, Beijing, China,* [4]*Department of Endocrinology and Metabolism, Guangdong General Hospital, Guangzhou, China,* [5]*Department of Endocrinology and Metabolism, Tongji Hospital, Huazhong University of Science and Technology, Wuhan, China,* [6]*Department of Endocrinology and Metabolism, West China Hospital of Sichuan University, Chengdu, China,* [7]*Department of Endocrinology and Metabolism, Beijing Hospital of Ministry of Public Health, Beijing, China,* [8]*Department of Endocrinology and Metabolism, the Second Affiliated Hospital of Harbin Medical University, Harbin, China*

Background: To compare the efficacy and safety of once-daily insulin glargine plus gliclazide modified release (MR) combination therapy versus twice-daily premixed insulin monotherapy in Chinese type 2 diabetic patients insufficiently controlled by oral antidiabetic agents (OADs).

Methods: In a 12-week, multicenter, randomized, parallel group clinical trial, patients with poor glycemic control (fasting plasma glucose ≥7.0 mmol/L and 7.5% <hemoglobin A1c (A1C) ≤10%) on OADs were randomized to the treatment groups for combination therapy ($n$ = 52) or monotherapy ($n$ = 53). Continuous glucose monitoring were carried out over two 72-h periods, at the beginning and the end of the study, and the data was used to calculate the 24 h mean blood glucose (24 h MBG), mean amplitude of glycemic excursions (MAGE), standard deviation of blood glucose (SDBG), and the mean of daily differences (MODD).

Results: The mean A1C decrease from baseline to study end was significant for both treatment groups (combination therapy: −1.23 ± 0.92%; insulin monotherapy: −1.02 ± 1.04%); moreover, the combination therapy group showed a significantly more robust A1C decrease ($P$ = 0.0308). Both therapies significantly reduced the 24 h MBG (both, $P$ < 0.001), but neither produced a significant effect on glycemic variability, calculated as MAGE, SDBG, and MODD. In addition, the effects on rates of hypoglycemic episodes were similar between the two therapies.

Conclusions: Chinese patients with type 2 diabetes inadequately controlled with OADs attained greater benefit from once-daily insulin glargine plus gliclazide MR regimen than from a twice-daily premixed insulin regimen.

### 0035

### Psychometric properties of the diabetes treatment satisfaction questionnaire in a large Japanese outpatient sample

Education and Care Delivery

Y. Kabeya[1,2], J. Uchida[2], M. Tomita[2], T. Katsuki[2], Y. Oikawa[2] and A. Shimada[2]

[1]*Tokai University Hachioji Hospital, Tokyo, Japan,* [2]*Tokyo Saiseikai Central Hospital, Tokyo, Japan*

Objectives: The Diabetes Treatment Satisfaction Questionnaire (DTSQ) is an instrument to assess treatment satisfaction and perceived frequencies of hyper- or hypoglycemia in patients with diabetes. The instrument has been translated into many languages. The Japanese version was published in 2000 and has been accepted widely so far. However, few studies have evaluated the psychometric properties. The aim of the present study was to evaluate the psychometric properties of the DTSQ Japanese version in a large outpatient sample.

Methods: A total of 1,065 patients with diabetes who attended the diabetes clinic of Saiseikai Central Hospital completed the DTSQ. Data on clinical characteristics of the patients were also collected from their medical records. Of the 8 items in the DTSQ, 6 items related to treatment satisfaction (current treatment, treatment convenience, treatment flexibility, treatment understanding, treatment recommendation, and treatment continuation) were analyzed. The reliability of the DTSQ scale of treatment satisfaction was evaluated by Cronbach's alpha coefficient. The construct validity was examined by confirmatory factor analysis. Finally, the overall satisfaction score (sum of the scores in the 6 items) was compared across different HbA1c levels (HbA1c <6.0%, 6.0–7.9%, 8.0–9.9% or 10.0-%) and treatment options (use of insulin therapy or oral medication).

Results: The mean age of the patients was 64.2 ± 12.8 years and men accounted for 79.3%. The mean HbA1c level was 7.6 ± 1.2%. A total of 393 patients (36.9%) received insulin therapy. The analysis revealed the reliability of the DTSQ was satisfactory with Cronbach's alpha of 0.91. In the confirmatory factor analysis, a one-factor model which took into account the correlation of the error terms for a pair of items (the item concerning treatment flexibility and the item concerning treatment convenience) demonstrated an excellent fit (chi-square / degrees of freedom < 3, the root mean squared error of approximation = 0.042 and the confirmatory fit index = 0.996). The analysis indicated all 6 items of treatment satisfaction in the DTSQ had substantial loading, with the standardized loadings ranging from 0.65 to 0.90. The overall satisfaction score decreased as HbA1c elevated. Lower satisfaction scores were observed in patients with insulin therapy, whereas use of oral medication did not affect the treatment satisfaction.

Conclusions: An analysis of psychometric properties in a large outpatient sample demonstrated that the Japanese version of the DTSQ was found to be a reliable instrument for evaluating treatment satisfaction, which might be useful for finding key elements of patients' satisfaction.

### 0038

### Metabolic syndrome and risk of severe white matter hyperintensities in Japanese adults

Prediabetes and Metabolic Syndrome

J. Uchida, Y. Kabeya, K. Kato, M. Tomita, T. Katsuki, Y. Oikawa and A. Shimada

*Saiseikai Central Hospital, Mita 1-4-17, Minato-ku/Tokyo, Japan*

Objectives: White matter hyperintensities (WMHs) are frequently detected on MRI in healthy people and known to be related to dementia. Several studies have reported that metabolic syndrome (MetS) is associated with WMHs. One study in Korea reported that hypertension had a great influence on having WMHs among the metabolic risk factors. In this study, we examined the effect size of the association between each metabolic factor and WMHs in Japanese adults. Moreover, the dose-response relationship between the number of metabolic risk factors and the presence of WMHs was investigated.

Method: We performed a cross-sectional study at one general hospital in Japan. A total of 1,341 adults aged 62 ± 10 years who underwent a medical checkup including brain MRI from 2007 to 2011 were invited to the study. Severity of WMHs was rated on a scale of 0–3 according to the Fazekas scale. Severe WMHs were defined as the presence of lesions with Fazekas grade 3. MetS was diagnosed using the criteria of the Japan Society for the Study of Obesity. According to the criteria, a person with MetS must have central obesity (waist circumference ≥85 cm for men, ≥90 cm for women) plus any two of the following three metabolic risk factors: 1) hypertension (systolic blood pressure ≥130, diastolic blood pressure ≥85 mmHg), 2) hyperglycemia (fasting plasma glucose ≥110 mg/dL), 3) dyslipidemia (HDL <40 mg/dL, TG ≥150 mg/dL). The associations of these metabolic risk factors with the presence of severe WMHs were evaluated using logistic regression analysis, which was adjusted for age, sex, smoking status and alcohol intake.

Results: Of the 1,341 adults, 264 had MetS. Severe WMHs were diagnosed in 249 adults. A significant association was observed between MetS and severe WMHs [OR = 1.69, 95% CI = 1.18–2.43]. The ORs of central obesity, hypertension, hyperglycemia, and dyslipidemia for the presence of severe WMHs were 1.15, 1.55, 1.24 and 1.11, respectively. The odds of having severe WMHs increased with the number of metabolic risk factors. Compared with adults having no metabolic risk factor, those with 1–2 and 3–4 were more likely to have severe WMHs with ORs of 1.23 (95% CI = 0.78–1.91) and 1.98 (95% CI = 1.20–3.27).

Conclusions: The present study suggested that the presence of multiple metabolic risk factors could be a risk for future dementia through the development of

© 2016 The Authors. Journal of Diabetes Investigation published by Asian Association for the Study of Diabetes (AASD) and John Wiley & Sons Australia, Ltd severe WMHs. Among the metabolic risk factors, hypertension might be the most potent risk for WMHs.

### 0039

### Comparison of intact proinsulin/ insulin ratio between the non-elderly and elderly diabetic patients

Insulin Secretion

H. Yoshino[1], T. Hirose[1], G. Yoshino[2] and K. Kawakami[2]

[1]*Division of Diabetes, Metabolism, and Endocrinology Department of Internal Medicine, Tokyo, Japan,* [2]*Shin-suma Hospital, Kobe, Japan*

AIM: Insulin is known to be synthesized in the β-cells from preproinsulin. After leaving the signal peptide preproinsulin becomes proinsulin. Disproportionate hyperproinsulinemia is known as an indicator of β-cell dysfunction in type 2 diabetes. Proinsulin is finally separated into C-peptide and insulin by two enzymes, prohormone convertase 1/3 (PC1/3) and 2 (PC2). It is known that measuring plasma proinsulin/insulin (P/I) ratio can be one of methods to estimate insulin secretory mechanism from pancreatic β-cells. The present study was conducted to examine whether insulin secretory mechanism of elderly diabetic patients differs from that of non-elderly diabetic patients.

METHOD: The study subjects consist of 172 patients. They were separated into 4 groups according to their age (below 64 and above 65 years) and an association of type 2 diabetes. Forty six elderly diabetic (mean age 74.5 ± 6.2, HbA1c (NGSP) 7.5 ± 1.4%), 27 elderly non-diabetic (mean age 76.9 ± 8.1), 48 non-elderly diabetic (mean age 50.8 ± 10.4, HbA1c 7.8 ± 1.5%), 51 non-elderly non-diabetic (mean age 46.6 ± 13.0) subjects were enrolled after informed consent was given. Diabetic subjects with insulin treatment or with DPP-4 inhibitors were excluded. Age, intact proinsulin/insulin (PI/I) ratio and HbA1c were investigated. Plasma insulin and intact proinsulin were measured by immunoradiometric and by chemiluminescence assay (MLT Research ltd., USA), respectively. To avoid breakdown of proinsulin during assay, incubation for reaction was done overnight at 4°C. ANOVA were employed for comparison of intact proinsulin and PI/I ratio between the two groups. Spearman's correlation coefficient by rank test were also employed. Simple regression analysis for PI/I ratio was performed with respective factors as independent variables (sex, age, BMI). Thereafter, multivariate regression analysis were performed. Data are expressed as mean±SD.

RESULT: The PI/I ratio of the diabetic groups were higher than that of non-diabetic groups in elderly group (0.19 ± 0.13 vs 0.11 ± 0.06, $P < 0.01$). PI/I ratio of the diabetic groups were higher than that of non-diabetic groups in non-elderly group (0.17 ± 0.15 vs 0.09 ± 0.09, $P < 0.01$). PI/I ratio correlated with age in non-diabetic. (r = 0.5, $P < 0.01$). Sex and age were the independent factors determining the age of PI/I ratio in non-diabetic group.

CONCLUSION: Insulin processing system is deteriorated not only in the non-elderly diabetic but also in elderly diabetic patients. Slight but significant relationship between PI/I ratio and age in non-diabetic group may indicate the accuracy of this proinsulin assay system and age-related deterioration of insulin processing system in non-diabetic subjects.

### 0040

### The efficacy of combined treatment with standard doses of tofogliflozin and mirabegron for old diabetes men with nocturia

Genetics and Epigenetics

Y. Suzuki

*HDC Atlas Clinic, Tokyo, Japan*

The sodium-glucose co-transporter 2 (SGLT2) inhibitors represent novel therapeutic approaches in the management of type 2 diabetes mellitus. Several compounds are already available in many countries (dapagliflozin, canagliflozin, empagliflozin, ipragliflozin, luseogliflozin and tofogliflozin). Among them, plasma half-life of tofogliflozin is shorter. The half-life (t1/2) of tofogliflozin is 5.4 h, while that of other SGLT2 inhibitors are over 10 h. The short half-life of tofogliflozin may provide clinical benefits compared to other SGLT2 inhibitors from the viewpoint of risk reduction of nocturnal hypoglycemia, nocturnal increase of urine volume, and frequency of nocturnal urination (1,2)

The drugs (solifenacin, isoproterenol and mirabegron) have urinary bladder contraction effect. Mirabegron is used for the treatment of symptoms associated with overactive bladder syndrome. It selectively stimulates the β3 -adrenoreceptor, which relaxes the detrusor muscle. This improves urine storage by distension of the bladder body.

One of side effects of SGLT2 inhibitors is dry mouth. In addition, common side-effects of solifenacin were dry mouth and constipation. Due to this reason, mirabegron is better treatment for diabetic patients under SGLT2 inhibitors treatment than solifenacin.

Therefore, to suppress frequency of nocturnal urination caused by SGLT2 inhibitors treatment, we examined the efficacy of combined treatment with standard doses of tofogliflozin and mirabegron. In result, two cases provided satisfactory therapeutic effect and improved nocturia.

Case 1: 72 y/o man. Height was 175 cm, and weight was 70 kg. Duration of diabetes was 11 years. After taking tofogliflozin 20 mg, and after taking mirabegron 50 mg, his nocturia relieved promptly. Case 2: 73 y/o man. Height was 183 cm, and weight was 69 kg. Duration was 9 years. After taking tofogliflozin 20 mg, and after taking mirabegron 50 mg, his nocturia relieved promptly.

These two cases suggested a solution that diabetic patients under SGLT2 inhibitor treatment can safely receive the merit without worrying about the increase of frequency of nocturnal urination. To our knowledge, the efficacy of combined treatment with standard doses of tofogliflozin and mirabegron is a new finding in the treatment of SGLT2 inhibitor treatment for old men who are suffering from overactive bladder and/or nocturia.

REFERENCES

1) Life Style Medicine vol.9 no.1 2015(in Japanese)

2) Sanofi, Aleway 20 mg Tablet (Tofogliflozin Hydrate), CTD, March, 2014: Phase Istudy, CSG010JP study, in Japanese Online: Available from URL:http://www.pmda.go.jp/drugs/2014/P201400036/index.html

### 0041

### Quality of life (QOL) change after the long-term treatment of sodium glucose transporter 2 inhibitor (SGLT2i) in Japanese

Therapeutics of Diabetes

Y. Suzuki

*HDC Atlas Clinic, Tokyo, Japan*

OBJECTIVES: To know the QOL change and adverse effect after the SGLT2i treatment, we examined the questionnaire in Japanese.

METHOD: 188 patients who had taken SGLT2i treatment and 369 patients who had not taken the treatment were subjected. They were examined by questionnaire regarding QOL and adverse effect. This study was conducted during November to December of 2014 in Tokyo.

RESULT: Between the two groups, there were not significant differences of frequency in the improved QOL items such as, intimacy in the family (3.2% vs.2.4%), performance of work (6.4% vs. 3.5%), social activity(1.1% vs. 1.4%), physical wellness(11.2% vs. 6.8%), everyday activities(6.9% vs. 4.9%), and exchanges with friends(3.2% vs. 2.2%). However, in SGLT2i-treated groups, frequencies of self-esteem(3.2% vs. 0.6%) and physical wellness(4.3% vs. 1.1%) were significantly higher than SGLT2i non-treated group ($P < 0.05$).

As for eating habit, compensatory overeating (COE) was significantly higher in SGLT2i-treated group (19.7%) than SGLT2i non-treated group (10%, $P < 0.05$). Negligence state was significantly higher in SGLT2i group (30.3%) than in SGLT2i non-treated group(16.5%).

As for adverse effect, dry mouth (4.8% vs. 0.8%) and frequent urination(8% vs. 3.8%)were significantly higher in SGLT2i group than SGLT2i non-treated group. Although not significant, the frequency of muscle power loss is likely to be lower in SGLT2i group than SGLT2i non-treated group.

CONCLUSION: SGLT2i treatment has an enough advantage for improving the QOL of diabetes patients, especially in self-esteem and physical wellness. Also, frequency of COE together with Negligence state is highly found in the group under SGLT2i therapy, which possibly leads to weight stability. The patients of diabetes are likely to be satisfied with this SGLT2i treatment, because they can eat more than before as they like, even sometime they become negligent. This

© 2016 The Authors. Journal of Diabetes Investigation published by Asian Association for the Study of Diabetes (AASD) and John Wiley & Sons Australia, Ltd change of life style leads to the improvement of self esteem and physical wellness.

Despite that this therapy of SGLT2i has some adverse effect like dry mouth and frequent urination, the improvement of QOL is more valuable for the patients' long-term way of life.

If physicians are careful to educate the patients to prevent dehydration, it will be better to start the SGLT2i therapy during hot season because dry mouth and frequent urination must occur unlikely due to much sweating in summer.

### 0043

### Retrospective postmarket result of sodium glucose transporter 2 inhibitor (SGLT2i) therapy in Japanese, targeting HbA1c improvement and weight reduction

Therapeutics of Diabetes

Y. Suzuki
*HDC Atlas Clinic, Tokyo, Japan*

Objectives: Premarket clinical trial of dapagliflozin suggested that dapagliflozin has attained the improvement of HbA1c and/or weight with high percentage in Japanese. We aimed to confirm the effect of SGLT2i therapy at the postmarket diabetes department.

Method: Among the diabetes patients at our clinic, we examined retrospectively the result of HbA1c and weight loss towards the patients who had continued the treatment of SGLT2i therapy for over six months.

Result: In result, 136 patients of diabetes were subjected. After three months of SGLT2i therapy (by ipragliflozin, luseogliflozin, dapagliflozin or tohogliflozin), HbA1c improved by 0.7% and weight reduced by 2.4 kg on average. Percentage of the group who had both reduced HbA1c and reduced weight was 70.6%. Percentage of the group who had reduced HbA1c but not reduced weight was 7.4%. Percentage of the group who had reduced weight but not reduced HbA1c was 20%. Percentage of no reduction of weight and HbA1c was 2.2%. Hence, 97.8% improved the improvement of HbA1c and/or weight.

After six months of SGLT2i therapy, HbA1c improved by 0.8% and weight reduced by 3.0 kg on average. Percentage of the group who had both reduced HbA1c and reduced weight was 53.7%. Percentage of the group who had reduced HbA1c but not reduced weight was 10.3%. Percentage of the group who had reduced weight but not reduced HbA1c was 31.6%. Percentage of no reduction of weight and HbA1c was 4.4%. Hence, 95.6% improved the improvement of HbA1c and/or weight.

Conclusion: This study confirmed the premarket data of dapagliflozin trial are right in terms of that SGLT2i therapy promises the improvement of HbA1c or weight reduction with the hope over 95%. Also the difference of three months data and six months data suggests that weight reduction is more sustainable than the improvement of HbA1c. The both improvement of HbA1c and weight reduction is likely to go weak with time. Therefore, SGLT2i treatment has more promising future effect on weight reduction rather than HbA1c reduction. As for the mechanism, we previously reported that SGLT2i treatment is likely to induce compensatory overeating (COE), which hampers the reduction of extreme weight loss as well as the improvement of HbA1c. We speculate that COE might trigger the worsening of HbA1c, thereby reducing the percentage of patients who maintained the improvement of HbA1c.

### 0044

### A case of type 2 diabetes who improved severe leg edema and skin complication by treatment of SGLT2 inhibitors

Clinical Diabetes

Y. Suzuki
*HDC Atlas Clinic, Tokyo, Japan*

In the treatment of diabetes, by oral administration of pioglitazone, lower leg edema is observed frequently. Women are likely to complicate the leg edema. We experienced a patient who had a lesion that showed festering skin and the stagnant vein depression phenomenon, complicated by dermatitis.

78-year-old woman. Duration of diabetes was 24 years. She has been under insulin therapy with pioglitazone. With pioglitazone, her HbA1c had dramatically improved. The best level of recent HbA1c was 5.8%.

However, leg edema occurred not only due to pioglitazone, but also due to sleep apnea syndrome. The leg edema was also resistant to diuretics. Lower leg edema did not improve by furosemide 40 mg and by mix of hydrochlorothiazide 12.5 mg and losartan potassium 50 mg

Surprisingly, after administration of ipragliflozin 50 mg, one of SGLT2 (sodium glucose co-transporter 2) inhibitors, leg edema disappeared in 2 weeks. Dermatitis improved, and purulent lesions also improved. Several other SGLT2 inhibitors such as depagliflozin have same effect on the improvement of dermatitis.

In conclusion, although a single case report is insufficient to determine pathogenesis, the present case does suggest the beneficial effect of SGLT2 inhibitor, on skin complication caused by leg edema due to pioglitazone and/or sleep apnea syndrome.

References

1. Mudaliar S, et al.: Thiazolidinediones, peripheral edema, and type 2 diabetes: incidence, pathophysiology, and clinical implications. Endocr Pract. 9:406–16, 2003
2. Abbade LP et al.: Venous ulcer: clinical characteristics and risk factors. Int J Dermatol. 50:405–11, 2011
3. Ely JW et al.: Approach to leg edema of unclear etiology. J Am Board Fam Med. 19:148–60, 2006.

### 0045

### Association between plasma adipocytokine concentrations and microvascular complications in patients with type 2 diabetes mellitus: a systematic review and meta-analysis of controlled cross-sectional studies

Microvascular Complications

A. J. Rodriguez[1,3], T. Neeman[1], C. A. Mastronardi[1], G. Paz-Filho[1] and V. S. Nunes[2]
[1]*Australian National University, Canberra, ACT, Australia,* [2]*State University/UNESP, Sao Paulo, Brazil,* [3]*Monash University, Melbourne, Victoria, Australia*

Objectives: Patients with diabetes have increased risk of developing microvascular complications. Adipocytokines have been variously associated with these complications in observational clinical studies. However, there are no comprehensive data examining the associations between adipocytokines concentrations and the presence of these complications.

Methods: We performed a systematic review of cross-sectional studies comparing circulating adipocytokines in patients with type 2 diabetes (T2D) who were affected by at least one microvascular complication, with T2D patients without these complications. Relevant studies were retrieved from MEDLINE, EMBASE, Scopus and Cochrane databases. Study quality was evaluated using a modified Newcastle-Ottawa Quality Assessment Scale. Meta-analysis was performed using an inverse-variance model. Standardised mean differences (SMD) and 95% confidence intervals (CI) were calculated for leptin and adiponectin. Heterogeneity was determined by the Q and I2 statistics, from which fixed or random effects models were applied.

Results: 554 abstracts were identified; 28 studies satisfied our inclusion/exclusion criteria. Study quality ranged from 4–10 (out of 11). Higher leptin levels were associated with the presence of microalbuminuria (SMD = 0.41; 95%CI = 0.14, 0.67; $n$ = 901; $P$ = 0.0003) and macroalbuminuria (SMD = 0.68; 95%CI = 0.30, 1.06; $n$ = 406; $P$ = 0.0004). Similarly, higher leptin levels were associated with the presence of neuropathy (SMD = 0.26; 95%CI = 0.07, 0.44; $n$ = 609; $P$ = 0.008). Higher adiponectin levels were associated with the presence of microalbuminuria (SMD = 0.55; 95%CI = 0.29, 0.81, $n$ = 274; $P < 0.001$) and macroalbuminuria (SMD = 1.37; 95%CI = 0.78, 1.97, $n$ = 246; $P < 0.00001$). In addition, higher adiponectin levels were associated with neuropathy (SMD = 0.25; 95%CI = 0.14, 0.36; $n$ = 1516; $P < 0.00001$) and retinopathy (SMD = 0.38; 95%CI = 0.25, 0.51; $n$ = 1306; $P < 0.00001$).

Conclusions: This systematic review and meta-analysis suggests that blood leptin and adiponectin levels are higher in patients with diabetes and microvascular complications, making these adipokines potentially relevant as therapeutic targets or biomarkers of diabetic microvascular complications. Studies were limited by cross-sectional design, thus large prospective analyses are required to confirm these findings independent of other risk factors, and to the determine their causality.

© 2016 The Authors. Journal of Diabetes Investigation published by
Asian Association for the Study of Diabetes (AASD) and John Wiley & Sons Australia, Ltd

### 0046

### The efficacy and safety of LY2963016 insulin glargine (LY IGlar) in Japanese patients with type 1 diabetes mellitus previously treated with Lantus® insulin glargine (IGlar): a subgroup analysis of the ELEMENT-1 study

Therapeutics of Diabetes

A. Takahashi[1], D. Chida[2] and H. Nishiyama[2]

[1]*Nippon Boehringer Ingelheim Co., Ltd., 2-1-1, Osaki, Shinagawa-ku, Tokyo, Japan,* [2]*Eli Lilly Japan K.K., 7-1-5, Isogamidori, Chuo-ku, Kobe, Japan*

Background: LY2963016 Insulin Glargine (LY IGlar) is a biosimilar to Lantus® insulin glargine (IGlar). The 52-week ELEMENT-1 study (a multinational, phase 3, open-label, randomized controlled study; 24-week main treatment, 28-week extension; total of 535 patients treated) demonstrated that both LY IGlar and IGlar, when used in combination with pre-meal insulin lispro, have similar efficacy and safety profiles in patients with type 1 diabetes mellitus (T1DM). Moreover no clinically meaningful efficacy and safety differences were noted between LY IGlar and IGlar in the subgroup of patients treated with IGlar prior to the study entry. The purpose of this analysis is to evaluate further similarity of LY IGlar vs. IGlar in another subgroup of 88 Japanese T1DM patients participating in ELEMENT-1 who were treated with IGlar prior to the study entry.

Method: Patients on IGlar prior to the study entry used unit-to-unit dose conversions to LY IGlar or IGlar at randomization and then followed treat-to-target titration. Primary endpoint (change in HbA1c from baseline to the 24-week endpoint), other efficacy and safety outcomes of LY IGlar and IGlar at/over 24 and 52 weeks were compared in the subgroup of Japanese patients treated with IGlar prior to the study entry.

Results: No clinically meaningful differences were observed in the primary endpoint (Least square mean HbA1c change of –0.235% and –0.259% in LY IGlar and IGlar, respectively, the difference between treatments, 0.024%; 95% confidence interval, –0.256 to 0.303) and in other efficacy and safety outcomes: proportion of patients achieving endpoint HbA1c target (less than 7.0%), changes of insulin dose (basal, prandial and total), and body weight change from baseline at the 24 week and the 52-week endpoint; total and nocturnal hypoglycaemia incidence and rate, incidence of treatment-emergent adverse event, incidence of allergic event related to study insulin, and incidence of detectable antibodies over 52 weeks.

Conclusions: The results from Japanese patients with T1DM treated with IGlar prior to the study entry were consistent with findings of similar efficacy and safety profiles of LY IGlar and IGlar observed in the total population treated with IGlar prior to the study entry of the ELEMENT-1 study. The numerical results might not be extrapolated to general Japanese patients due to limited sample size.

### 0048

### *ADAMTS9* genetic variant is associated with diabetic retinopathy in Chinese patients with type 2 diabetes

Microvascular Complications

D. Peng, R. Zhang, C. Hu and W. Jia

*Shanghai Diabetes Institution, Shanghai Jiao Tong University Affiliated Sixth People's Hospital, Shanghai, China*

Objective: Diabetic nephropathy and retinopathy are two major microvascular diabetes complications with a high concordance rate in patients with diabetes. *ADAMTS9* rs4607103 was identified as a susceptible locus for type 2 diabetes in a genome-wide association study. And it was also reported to be associated with insulin resistance. This study aimed to test the effect of this locus on diabetic nephropathy and retinopathy in Chinese patients with type 2 diabetes.

Methods : In stage 1, we recruited 1,251 patients with type 2 diabetes, including 313 patients with diabetic retinopathy but no nephropathy, 419 patients with diabetic nephropathy but no retinopathy, 281 patients with both diabetic retinopathy and nephropathy, and 238 control subjects with diabetes for 10 years and no diabetic retinopathy or nephropathy. In stage 2, 594 patients with type 2 diabetes were recruited, including 201 patients with retinopathy and 393 patients with diabetes for 5 years but no retinopathy. *ADAMTS9* rs4607103 was genotyped using the primer extension of multiplex products with detecting by matrix-assisted laser desorption ionization-time of flight mass spectroscopy using a MassARRAY Compact Analyzer (Sequenom, San Diego, CA, USA). Fundus photography was performed to diagnose and classify diabetic retinopathy. The 24 h albumin excretion rate (AER) and eGFR calculated using a formula developed by the Modification of Diet in Renal Disease study group with adjustment for Chinese ethnicity were used to assess nephropathy.

Results : In stage 1 analysis, we found rs4607103 was significantly associated with diabetic retinopathy with adjustment for confounding factors, including $HbA_{1C}$ levels, duration of diabetes, systolic and diastolic blood pressure and BMI (OR = 0.746, 95% CI 0.608–0.915, $P = 0.0048$). However, we did not find any association of this SNP with diabetic nephropathy (OR = 0.995, $P = 0.961$). In stage 2 analysis, rs4607103 showed similar effect on diabetic retinopathy (OR = 0.797, 0.606–1.050, $P = 0.106$), and meta-analysis showed a significant association between rs4607103 and diabetic retinopathy (OR = 0.764, 95% CI 0.648–0.900, $P = 0.0013$). It was also associated with levels of diabetic retinopathy ($P = 0.0096$ for trend analysis).

Conclusion: In this study, we found *ADAMTS9* rs4607103 was associated with diabetic retinopathy in the Chinese patients with type 2 diabetes.

### 0052

### Trends of diabetes incidence in the last decade from National Health Insurance claimed data

Epidemiology

S. O. Song[1], H. M. Kim[2], D. W. Kim[1], J. Y. Nam[1], K. H. Park[1] and Y. D. Song[1]

[1]*National Health Insurance Service Ilsan Hospital, Goyang, Republic of Korea,* [2]*Chung-Ang University Hospital, Seoul, Republic of Korea*

Background: Epidemiological data is a useful to estimate the necessary manpower and resources in frequent chronic disease for disease control and prevention. However, there were only cross-sectional claimed data about total number of diabetics only in 2003. We wanted to evaluate diabetes incidence and identify its trends from the claimed data.

Methods: We extracted claimed data about diabetes as principal and 1st additional diagnosis in National Health Insurance from Jan. 2003 to Dec. 2012. We investigated the number of newly claimed diabetic subjects, the number of claims and the demographic characteristics of their population.

Results: Total numbers of claimed cases and populations with diabetes increased. However, the annual number of newly claimed diabetic subjects showed a declining decreased in the last decade. The data showed increasing proportion of younger age and sharply decreasing proportion of older age in newly claimed diabetic subjects.

Conclusion: There were increasing numbers of newly claimed diabetic subjects in young age for last 10 years; we may need to manage the growing numbers of diabetic patients for longer period of their life because Korea is rapidly becoming an aging society

### 0053

### Nutritional regulation of lipid metabolism and hyperglycemia in obesity and non-alcoholic fatty liver disease

Obesity

K. O[1,2], V. Sid[1,2], N. Wu[1,2], L. Sarna[1,2] and Yaw. L. Siow[1,2]

[1]*St. Boniface Hospital Research Centre, Winnipeg, MB, Canada,* [2]*University of Manitoba, Winnipeg, MB, Canada*

Chronic consumption of high-fat diets is associated with obesity and non-alcoholic fatty liver disease (NAFLD). NAFLD is a condition in which too much fat accumulates in the liver without a history of alcohol abuse. Patients with NAFLD are at a high risk for cardiovascular disease and diabetes since NAFLD is closely associated with hyperlipidemia and hyperglycemia. Liver plays an important role in lipid and carbohydrate metabolism. Regulation of HMG-CoA reductase activity in the liver is crucial in maintaining cholesterol homeostasis. When cholesterol biosynthesis or metabolism is perturbed, cholesterol accumulation in the liver and its elevation in the blood can occur. The aim of our study was to investigate the mechanisms by which chronic high-fat diet consumption affected hepatic cholesterol

© 2016 The Authors. Journal of Diabetes Investigation published by Asian Association for the Study of Diabetes (AASD) and John Wiley & Sons Australia, Ltd biosynthesis and the impact of folic acid supplementation on fatty liver and hyperglycemia. Male C57BL/6 mice were fed a control diet (10% kcal fat), a high-fat diet (60% kcal fat) or a high-fat diet supplemented with folic acid for 5 weeks. Mice fed a high-fat diet had an increased body weight and displayed NAFLD features including hepatic lipid accumulation, oxidative stress, inflammatory chemokine expression and hyperlipidemia. High-fat diet feeding resulted in an increase in serum lipid levels (total cholesterol, triglyceride) and hyperglycemia. Hepatic cholesterol biosynthesis was increased due to activation of HMG-CoA reductase. Further analysis revealed that the DNA binding activity of sterol regulatory element binding protein (SREBP)-2 was increased in the liver of mice fed a high-fat diet. Folic acid supplementation attenuated high fat diet induced hyperglycemia and hepatic cholesterol biosynthesis. Such a beneficial effect was mediated, in part, through AMPK signaling pathway, which was independent of body weight change. In conclusion, our results suggest that during high-fat feeding, SREBP-2 activation up-regulates HMG-CoA reductase gene expression which, in turn, contributes to hepatic lipid accumulation and hypercholesterolemia. Aberrant cholesterol biosynthesis exacerbates liver injury in NAFLD. The ability of folic acid to restore AMPK function may contribute to the improvement of cholesterol metabolism and glucose that are impaired by chronic high fat diet consumption. Regulation of *de novo* cholesterol synthesis and carbohydrate metabolism may have a therapeutic implication in patients with metabolic disorders such as NAFLD and metabolic syndrome.

### 0054
### Effects of linagliptin in elderly patients with type 2 diabetes mellitus

Clinical Diabetes

M. Abe, T. Omoto, M. Shinozaki, S. Nisio, S. Antoku, M. Mifune, M. Togane and H. Ito
*Edogawa Hospital, Tokyo, Japan*

Objectives: The aim of this study was to examine the effects of linagliptin in elderly patients with type 2 diabetes.

Methods: Two hundred and sixteen patients with type 2 diabetes treated with 5 mg once daily linagliptin were studied as the treated set (TS) in order to analyze the safety of linagliptin. The subjects were divided into two groups: the elderly group (≥70 years of age, $n = 131$) and the non-elderly group (<70 years of age, $n = 85$). One hundred and forty-five (90 elderly and 55 non-elderly) subjects whose medications remained unchanged during the observation period were retrospectively investigated as the full analysis set (FAS) to assess the effectiveness of linagliptin for 6 months. The parameters were analyzed separately in the subjects receiving monotherapy and additional therapy (i.e. linagliptin added to the other antidiabetic agents).

Results: In the TS, adverse events were recorded in 44 subjects (34%) in the elderly group and 18 subjects (21%) in the non-elderly group. In the FAS, the HbA1c levels were significantly improved in subjects receiving monotherapy ($n = 48$) or additional therapy ($n = 42$) in the elderly group (7.5 ± 1.0% to 6.6 ± 0.8% and 7.9 ± 1.2% to 7.3 ± 1.1%, respectively), similar to the non-elderly group (7.4 ± 0.6% to 6.4 ± 0.6% and 7.7 ± 1.2% to 7.2 ± 1.4%, respectively). The change in the HbA1c levels (ΔHbA1c) at 6 months after the initiation of linagliptin therapy was not significantly different between the elderly and non-elderly groups (−0.8 ± 0.9 and −1.0 ± 0.8, respectively in the subjects receiving monotherapy, and −0.6 ± 0.7 and −0.5 ± 1.1, respectively in those receiving additional therapy). The ΔHbA1c value at 6 months was significantly associated with the body mass index in the elderly (regression coefficient: 0.078), and obesity (regression coefficient: −0.186) and the HbA1c values at baseline (regression coefficient: −0.399) in the non-elderly group.

Conclusions: Linagliptin is considered to be equally effective for elderly patients with type 2 diabetes and non-elderly patients, especially those with higher HbA1c values at baseline or non-obese patients.

### 0056
### The resolution of cardiac tamponade with levothyroxine replacement in a hypothyroid Patient: A Case Report

Thyroid and Osteoporosis

R. Jugueta, A. Lim-Teodoro and L. Bunoy
*World Citi Medical Center, Quezon City, The Philippines*

Objective: To present a case of cardiac tamponade as a consequence of untreated hypothyroidism that eventually resolved by utilizing a non-invasive approach.

Methods: We presented the clinical and laboratory data in our patient and reviewed published related literatures regarding the pathophysiology of cardiac tamponade in hypothyroid patients and its possible treatments.

Introduction: Cardiac tamponade is the abnormal accumulation of pericardial fluid that is sufficient to limit inflow of blood during diastole. A rare complication of hypothyroidism is cardiac tamponade. Pericardial effusion is a manifestation of advanced stage of hypothyroidism called myxedema, but a rare finding of mild hypothyroidism. The incidence of pericardial effusion in hypothyroidism is now infrequent with a rate of 3–6% due to early recognition in which the latter is detected. This is a case of a hypothyroid patient with cardiac tamponade that was eventually resolved by medical management of her hypothyroidism.

Case presentation: A 59 year old female, hypothyroidism patient, presents with cough, shortness of breath and crackles on lung examination. The patient was initially treated as a case of pneumonia. Due to persistence of difficulty of breathing, a cardiac pathology was suspected. She was referred to a cardiologist and two dimensional echocardiography revealed pericardial effusion with tamponade. The classic finding of Beck's triad, consisting of jugular venous distention, hypotension and muffled heart sounds were not found in our patient, hence, pericardiocentesis was not contemplated. Patient was then seen by an endocrinologist and she was started on Levothyroxine 25 mcg/tab OD, eventually resulting in regression of pericardial effusion and cardiac tamponade. She was discharged improved.

Conclusion: Pericardial effusion with cardiac tamponade is treated with drainage of the pericardial fluid known as pericardiocentesis. This case is unique because even with the presence of cardiac tamponade, the patient was hemodynamically stable, and the patient was treated thru optimization of medical therapy. This case highlights the importance of thyroid replacement therapy as a conservative treatment of pericardial effusion and cardiac tamponade.

### 0057
### A consideration of skin complication caused by treatment of SGLT2 inhibitor in Japanese

Therapeutics of Diabetes

Y. Suzuki
*HDC Atlas Clinic, Tokyo, Japan*

In Japan, skin disorders of SGLT2 inhibitors (SGLT2i) have been reported so many in the spring of 2014. We previously reported skin disorders in 17 cases (12%) among 142 cases [1]. It became uproar, because the disorders such as eruptions occur in a short period of time after oral administration.

Several coincidences give us hints to solve the mechanism. First; SGLT2i was released first from ipragliflozin which is prone to cause skin disorders than other SGLT2i products. Recent study revealed that ipragliflozin remains in the skin with high concentration than other SGLT2i such as dapagliflozin and canagliflozin [2]. Second; Drug eruption that occurs from the first day of oral one tablet suggests that allergic predisposition due to other certain environmental causes might have been potentially existed. SGLT2i might only became a trigger which makes the potential type I skin allergy apparent. Third; According to the reports from pharmaceutical companies, skin disorder incidence after SGLT2i prescription has decreased over the autumn and winter from the summer, suggesting that the SGLT2i associated skin disorder has seasonal variation in Japan. Interestingly, cypress dermatitis occurs during the spring season and PM2.5 dermatitis occurs from spring season to summer.

Hence, we speculate that the coincidences of the below elements induced the uproar. The first release of ipragliflozin possibly makes skin disorder apparent

© 2016 The Authors. Journal of Diabetes Investigation published by
Asian Association for the Study of Diabetes (AASD) and John Wiley & Sons Australia, Ltd socially. Cypress dermatitis by and PM2.5 dermatitis overlapped during the released season of SGLT2i products, thereby making this accidence severe socially.

In 2015, skin disorder from spring to summer has not become uproar unlike 2014. However, at our clinic, skin disorder matched the timing of cypress dermatitis and PM2.5 dermatitis period. While most of them were induced by empagliflozin, the severity was milder than that of ipragliflozin. These facts confirm us that, when we discuss about the skin disorder of SGLT2i, we should consider the difference of SGLT2i products and the timing of cypress dermatitis and PM2.5 dermatitis.

In conclusion, as Asian duct problem, PM2.5 has been recently reported about the correlation between the type I skin allergy, which become a social problem. It is hence easy and logically assumed that the PM2.5 causes health damage from 2013. This will help us to understand why the skin disorder by SGLT2i is so specifically seen in Japan, compared with other countries.

References

1. Diab.Res.Clic. Prac. 106, sup1:272, 2014
2. I-P-43 at The 58th Annual Meeting of the Japanese Diabetes Society. 2015.(in Japanese).

### 0058

### The effect of exercise during the treatment of ipragliflozin

Other Endocrine Topics

N. Kato and M. Kato

*Kato clinic of Internal Medicine, Takasago, Katsushika, Tokyo, Japan*

Aims: To evaluate the efficacy of ipragliflozin on Japanese type 2 diabetes mellitus patients with inadequate glycemic control despite conventional anti hyperglycemic dual therapy for over 6 weeks.

Materials and Methods: Eighteen patients with type 2 diabetes mellitus were administered 50 mg ipragliflozin once daily for 12 weeks. Primary endpoint was the change in glycated hemoglobin from baseline to week 12. And the effect of their physical activity levels were analyzed.

Subject: 83.3% were men, the mean ± standard deviation age, mean duration of diabetes, baseline HbA1c, fasting plasma glucose, body weight, body mass index were 54.1/9.5 years, 11.5/7.2 years, 8.4/0.9%, 168.8/48.1 mg/dL, 81.5/21.1 kg, 29.3/5.2 kg/m$^2$, with 88.9% of patients classified as obese(BMI≧25), according to the definition of obesity and visceral fat obesity in Japan. 77.8% were taking antidiabetic oral agents: biguanide 55.6%, thiazolidinediones 16.7%, dipeptidase-4 inhibitors 16.7% and sulfonylureas 5.6%.

Results: Glycated hemoglobin decreased significantly by 0.69% at week 12(adjusted mean difference from baseline; $P < 0.01$). At week 12, changes for body compositions from baseline with ipragliflozin were as follows: total body weight, –1.86 kg ($P < 0.01$); body mass index, –0.79 kg/m$^2$ ($P < 0.01$); fat mass, –1.31 kg ($P < 0.01$); skeletal muscle mass, –0.57 kg ($P < 0.01$); muscle mass, –0.92 kg ($P < 0.01$). The magnitude of reduction in HbA1c for vigorous exercise group was larger, but not significantly, than that of mild exercise group(less twice a week). By contrast, mild exercise group showed a significant decrease in skeletal muscle mass (–0.84 kg, $P < 0.05$) and muscle mass (–1.36 kg, $P < 0.05$) at week 12, while vigorous exercise group did not show any significant changes in these parameters. Although no major hypoglycemic episodes occurred.

Conclusions: Treatment with ipragliflozin was associated with a marked improvement in glycemic control in daily routine practice as well as with a reduction of total body weight and body mass index, without major side effects.

Statistical analysis; Efficacy variables were presented descriptively for changes over time as mean ± standard deviation. The changes from baseline were analyzed at each interval using Tukey's multiple comparison test to compare among strata, and Dunnett's multiple comparison test to compare time points with that of measured at baseline within a stratum. Statistical and data analyses were performed using SAS version 9.4(SAS Institute Japan, Ltd., Tokyo, Japan). Statistical comparisons were made using two-side tests at $\alpha = 0.05$ significant level.

This study was registered with UMIN CTR(no. UMIN000014388)

### 0059

### Potentiation of glucose-stimulated insulin secretion by GPR40-PLC-TRPC pathway in pancreatic beta cells

Islet Biology

H. Yamada[1], M. Yoshida[1], K. Ito[1], D. Katsuya[2], M. Kawakami[1], S.-e. Ishikawa[1], T. Yada[2] and M. Kakei[1]

*[1]First Department of Comprehensive Medicine, Jichi Medical University Saitama Medical Center, Saitama, Japan, [2]Department of Physiology, Jichi Medical University, Shimotsuke, Japan*

Background and aims: G-protein coupled receptors (GPCRs) are expressed in pancreatic beta cells. G protein-coupled receptor 40 (GPR40) contributes to medium- or long-chain fatty acids-induced amplification of glucose-stimulated insulin secretion (GSIS). Thus, its agonists are promising therapeutic target in type 2 diabetes. Recently we demonstrated that GLP-1, a ligand of pancreatic GPCR, activates a class of nonselective cation channel (NSCC) and enhances GSIS. The aim of the current study is to determine whether GPR40 signal interacts to NSCCs and to identify what type of channel is related to the NSCC.

Materials and methods: Islets of Langerhans were isolated by collagenase digestion from male Wister rats. Measurement of insulin secretion was performed with static incubations. Perforated whole-cell clamped currents were recorded at the holding potential of –70 mV in the presence of 100 μM tolbutamide or at –80 mV without tolbutamide. The latter potential was used to exclude the influence of KATP-channel current on NSCC-current changes induced by GPR40 agonist. $[Ca^{2+}]i$ was measured by dual-wavelength fura-2 microfluorometry.

Results: GPR40 agonist (fasiglifam 10 μM) potentiated GSIS at 16.7 mM glucose(control vs. fasiglifam 10 μM; 19 ± 0.3 vs. 26.2 ± 0.8 ng/mL/10 islets/hour), but not at 2.8 mM glucose (5.0 ± 0.1 vs. 6.1 ± 0.2). NSCC current was activated by fasiglifam at 5.6 mM glucose with 100 μM tolbutamide (control vs. fasiglifam 10 μM; –1.8 ± 0.3 pA/pF vs. –3.8 ± 1.2 pA/pF, $P = 0.016$, $n = 6$) and the consistent results were obtained at 5.6 mM glucose without tolbutamide at –80 mV. The current increase was prevented by the presence of 2-APB (a nonspecific NSCCs blocker) or BTP2 (transient receptor potential canonical; a TRPC channel blocker). BTP2 inhibited fasiglifam-induced $[Ca^{2+}]i$ in beta cells. Then we examined the relationship between GPR40 stimulation and phospholipase C (PLC) downstream of the GPR40 signal. In the presence of a PLC inhibitor U73122 both fasiglifam-induced GSIS increase and NSCC-current increase were inhibited (insulin:fasiglifam vs. fasiglifam + U73122; 29.7 ± 1.7 vs. 22.9 ± 0.7 ng/mL/10 islets/h, $P < 0.05$;NACC-current:fasiglifam vs. fasiglifam + U73122; –1.4 ± 0.3 pA/pF vs. –1.4 ± 0.9 pA/pF, $P = 0.99$). Fasiglifam did not increase cytosolic cAMP concentration of islets. Protein kinase A inhibitor H89 did not affect the potentiation of GSIS by fasiglifam at 16.7 mM glucose.

Conclusion: The present study demonstrates a novel mechanism underlying potentiation of GSIS by GPR40 in pancreatic beta-cells. Stimulation of GPR40-PLC-TRPC channel pathway potentiates GSIS by depolarization of the beta-cell plasma membrane.

### 0060

### Nutritional care of Fournier's gangrene patients

Behaviour and Nutrition

C.-W. Lin, M.-Y. Tu and M.-Y. Liu

*Chi Mei Medical Center, Tainan City/Yongkang Dist, Taiwan*

Objectives: Fournier's gangrene is a mixture of aerobic and anaerobic type of disease, it is a necrotizing fasciitis that occurs in the skin and subcutaneous soft tissue, and spread to the genitalia and perineum. Most of the patients were elderly with diabetes, hypertension, trauma, and local or systemic infection. The study aims to analyze the results and improvements obtained in nutritional treatment with Fournier's Gangrene.

Methods: 55-year-old male patient of a medical center in southern Taiwan check in to the clinic because the anus pain lasted for six days and with bloodshot. He was admitted by the physician's advice. Evaluation included Past medical history,

© 2016 The Authors. Journal of Diabetes Investigation published by Asian Association for the Study of Diabetes (AASD) and John Wiley & Sons Australia, Ltd

Habits, height, weight, BMI, blood biochemistry values change, drug usage, SGA and the examination, treatment and using 24 h dietary recall method. Therefore, evidence-based assessments and behavioral changes in patients with stage adjustment timely adjustment of diet and health education and give special nutritional content of meals. Results were tracked.

RESULTS: After visits for health education and tracking, appetite changed from eating 1200 calories 39 g protein progressed to 2100 calories high protein 103 g we set. Behavior change from the contemplation stage to the action stage. Nutrition diagnosis: food and nutrition related knowledge deficit, undesirable food choices. Moreover, because the regulation of the immune system including the use of special nutrients through the activity and more improvement on wound healing.

CONCLUSIONS: From the literature, we know that patient nutritional recommendations with intake of high-calorie Energy (30–35 kcal/kgw) and high-protein (1.5–2 g/kgw); and otherwise, nutrition therapy with Glutamine 0.285–0.48 g/kg/day and Arginine 14–30 g/day. This report is by practical experience obtained after sharing the care of patients.

### 0061

### Compensative hyper-insulinemia caused by chronic treatment of SGLT2 inhibitor: hypothesis

Therapeutics of Diabetes

Y. Suzuki
*HDC Atlas Clinic, Tokyo, Japan*

We previously reported that Compensative overeating (COE) could be a trouble to counteract the weight-loss caused by sodium glucose co-transporter 2 inhibitor (SGLT2i) (1). Thereafter, we added that predisposition of food shifted mainly to carbohydrate.

However, not all the patients do not have COE. Therefore, we should consider other different mechanism to solve the question why the weight of patients gets stable after chronic treatment of SGLT2i.

In literature, 4-weeks trial of empagliflozin indicated that after meal ingestion, glucose and insulin AUC decreased, whereas the glucagon response increased. Insulin sensitivity was improved. In the article, the authors (Ele Ferrannini, et al.) defined that the 4 weeks observation is “chronic” observation (2).

However, in Japanese, looking back to the result of clinical trial with luseogliflozin, there were no differences in AUC of plasma insulin concentration between baseline and 24 weeks (3). Glucose AUC decreased, but insulin AUC did not decreased. Insulin AUC was almost the same as baseline. This is in contrast to that of 4-weeks observation.

Therefore, these facts suggest that, in Japanese, COE and/or compensatory hyperinsulinemia mainly to carbohydrate must have occurred during 6 months chronic trial period.

Theoretically, compensatory overeating can induce insulin secretion from pancreas, leading to hyperinsulinemia. The hyperinsulinemia suppresses the hyperglycemia, leading to glycemic control. This is in contrast to the hyperinsulinemia based on the insulin resistance of obese type 2 diabetes.

We hence hypothesize that seemingly similar two hyperinsulinemias are different in the meaning of the phenomena based on insulin resistance or the phenomena based on COE and/or carbohydrate predispositional food change after SGLT2i treatment. And compensatory hyperinsulinemia on chronic phase of SGLT2 treatment might be an essential effect to keep body weight balance stable, despite the chronic constant loss of energy in urine.

REFERENCES:
1. Suzuki Y. Compensatory Overeating (COE) could be a Problem of Diabetes Diet Education after Sodium-Glucose Co-Transporter 2 Inhibitor Treatment. The 6th International Conference on Fixed Combination in the Treatment of Hypertension, Dyslipidemia and Diabetes Mellitus, Berlin, Germany: P 18 、2015.
2. Ele Ferrannini, et al. Metabolic response to sodium-glucose cotransporter 2 inhibition in type 2 diabetic patients. The Journal of clinical investigation 124: 499- 508, 2–14
3. Seino Y, et al. Efficacy and safety of luseogliflozin as monotherapy in Japanese patients with type 2 diabetes mellitus: a randomized, double-blind, placebo-controlled, phase 3 study. Curr Med Res Opin. 30:1245–55, 2014

### 0062

### 2013 ACC/AHA versus 2004 NECP ATP III Guidelines for the assignment of statin treatment in subjects with subclinical coronary atherosclerosis in Korea

Macrovascular Complications

W. J. Lee[1], C. H. Jung[1], C.-Y. Park[2] and J.-Y. Park[1]
*[1]University of Ulsan College of Medicine, Seoul, Republic of Korea, [2]Sungkyunkwan University School of Medicine, Seoul, Republic of Korea*

OBJECTIVES: The usefulness of the 2013 ACC/AHA guidelines for the management of blood cholesterol in the Asian population remains controversial. In this study, we investigated whether eligibility for statin therapy determined by the 2013 ACC/AHA guidelines is better aligned with the presence of subclinical coronary atherosclerosis detected by CCTA (coronary computed tomography angiography) compared to the previously recommended 2004 NCEP ATP III guidelines.

METHODS AND RESULTS: We collected the data from 5,837 asymptomatic subjects who underwent CCTA using MDCT during routine health examinations. Based on risk factor assessment and lipid data, we determined guideline-based eligibility for statin therapy according to the 2013 ACC/AHA and2004 NCEP ATP III guidelines. We defined the presence and severity of subclinical coronary atherosclerosis detected in CCTA according to the presence of significant coronary artery stenosis (defined as >50% stenosis), plaques, and the degree of coronary calcification. As compared to the 2004 ATP III guidelines, a significantly higher proportion of subject with significant coronary stenosis (61.8% vs. 33.8%), plaques (52.3% vs. 24.7%), and higher CACS (CACS >100, 63.6% vs. 26.5%) was assigned to statin therapy using the 2013 ACC/AHA guidelines ($P < 0.001$ for all variables). The area under the curves of the pooled cohort equation of the new guidelines in detecting significant stenosis, plaques, and higher CACS were significantly higher than those of the Framingham risk calculator.

CONCLUSIONS: Compared to the previous ATP III guidelines, the 2013 ACC/AHA guidelines were more sensitive in identifying subjects with subclinical coronary atherosclerosis detected by CCTA in an Asian population.

### 0063

### 1,25-Dihydroxyvitamin D decreases adipocyte triglyceride storage and affects NAD-sirtulin1 pathway in 3T3-L1 adipocytes

Obesity

E. Chang and Y. Kim
*Ewha Womans University, Seoul, Republic of Korea*

Increasing prevalence of obesity is a major public health concern due to its close association with metabolic disorders. Obesity is characterized by adipose expansion due to hypertrophy of preexisting individual adipocytes and/or hyperplasia due to formation of new adipocytes. Thus, to understand molecular mechanisms of adipocyte formation and alteration has been regarded as a good strategy for treating obesity and its associated metabolic syndrome. Accumulating evidence shows vitamin D deficiency is highly prevalent in obese people, indicating obesity might be an independent risk factor for vitamin D deficiency. Body fat content is negatively correlated with 25-hydroxyvitamin D (25(OH)D) concentrations, a blood marker for vitamin D status. However, the influence of vitamin D on adipocyte differentiation and lipid metabolism is largely unknown. The purpose of this study is to investigate the mechanisms by which the most hormonally active form of vitamin D calcitriol (1,25-dihydroxyvitamin D, 1,25(OH)2D) affects adipocyte hypertrophy and lipid metabolism and alters expression and activity of sirtulin1 (SIRT1), a NAD-dependent deacetylase regulating glucose and fat metabolism and oxidative machinery. Differentiated 3T3-L1 adipocytes were treated with various concentrations of 1,25(OH)2D at indicated time points. MTT cell proliferation assay was employed to demonstrate effect of 1,25(OH)2D on cell viability. The intracellular lipid content was evaluated by Oil Red O staining. Basal and isoproterenol-stimulated lipolysis were measured in culture medium to investigate the regulation of 1,25(OH)2D in adipocyte lipid metabolism. RT-PCR was performed to determine adipogenesis-related markers such as aP2, C/EBP$\alpha$, PPAR$\gamma$, and SCD1 and fatty acid oxidation-associated factors including CPT1$\alpha$, PGC1$\alpha$, and PPAR$\alpha$, Measurement of SIRT1 activity, NAD and NADH were performed using SIRT1 activity assay kit and NAD/NADH assay kit. 24 h incu-

© 2016 The Authors. Journal of Diabetes Investigation published by
Asian Association for the Study of Diabetes (AASD) and John Wiley & Sons Australia, Ltd bation of 1,25(OH)2D (1, 10, 100 nM) did not alter cell viability in differentiated 3T3-L1 cells. 1,25(OH)2D treatment (100 nM, 24 h) induced a significant reduction in intracellular lipid content and increase in basal and isoproterenol-induced lipolysis. Furthermore, mRNA expression of adipogenic genes such as aP2, C/EBPα, PPARγ, and SCD1 was significantly down-regulated by 1,25(OH)2D in 3T3-L1 adipocytes. In contrast, mRNA levels of fatty acid oxidation-associated markers including CPT1α, PGC1α, and PPARα were significantly increased in 1,25(OH)2D-treated adipocytes. Moreover, SIRT1 expression and SIRT1 activity level were significantly increased by 1,25(OH)2D treatment together with a significant reduction of NADH levels and increase of NAD/NADH ratio. These findings suggest that 1,25(OH)2D leads to decreased adipocyte fat storage and increased lipolysis which may relate to SIRT1 activity and NAD/NADH level.

### 0064

### A randomized crossover study of efficacy and safety of switching from insulin glargine to insulin degludec in children with Type 1 diabetes

Pediatric Endocrinology

T. Urakami, R. Kuwabara, Y. Mine, M. Aoki, M. Okuno and J. Suzuki

*Department of Pediatrics, Nihon University School of Medicine, Tokyo, Japan*

Objective: The aim of the study was to evaluate efficacy and safety of insulin degludec (IDeg) compared with insulin glargine (IGlar) in children with type 1 diabetes (T1DM).

Subjects and Methods: The study subjects consisted of 18 Japanese children, 11 males and 7 females, age 11.7 ± 2.1 (7.0–14.0) years, with T1DM. All subjects were non-obese with BMI of 20.9 ± 4. All the subjects previously used quick-acting insulin before each meal as bolus insulin, and IGlar once per day at bedtime as basal insulin. By use of a randomized crossover design, we compared fasting plasma glucose (FPG) and HbA1c levels, and frequency of overall and nocturnal episodes of confirmed hypoglycemia per patient-month and basal insulin dose per kg•body weight-day at the 12th week after using IGlar and IDeg. IDeg was initially injected at the same dose as IDeg given once per day at bedtime, but were then titrated to attain self-monitored FPG levels between 90 and 140 mg/dL during the 12-week study period. Confirmed hypoglycemia was defined as self-monitored PG levels <70 mg/dL, and severe episodes as having impaired consciousness or seizure requiring assistance from other persons Hypoglycemic episodes occurring between 11 pm and 7 am were classified as nocturnal.

Results: FPG and HbA1c (NGSP) levels at the 12th week after using IGlar and IDeg were 134.9 ± 24.7 and 133.8 ± 17.0 mg/dL for FPG, 7.8 ± 0.7 and 7.8 ± 0.8% for HbA1c, respectively. There were no significant differences of FPG and HbA1c levels between with IGlar and IDeg. Frequency of overall hypoglycemia was similar (4.2 ± 3.1 vs. 3.4 ± 2.5 episodes/mo) in the two basal insulin analogues, but nocturnal hypoglycemia from 22:00 to 6:59 was significantly lower with IDeg than with IGlar (1.8 ± 1.9 *vs.* 0.8 ± 1.0 episodes/mo, $P < 0.05$). No severe hypoglycemia occurred during the study period. The daily basal insulin dose was not significantly different between with IGlar and IDeg (0.48 ± 0.15 vs. 0.44 ± 0.16 kg/day).

Conclusion: These results suggest that IDeg given once at bedtime provides similar glycemic control to IGlar while lowering a risk of nocturnal hypoglycemia than IGlar in children with T1DM.

### 0065

### Non-invasive measurement of skin AGEs and its association with diabetic microvascular complications

Microvascular Complications

S. Murakami[1], T. Matsumura[1], M. Yamanaka[2,3], R.-I. Ohno[2], H. Kinoshita[1], T. Senokuchi[1], N. Ishii[1], K. Fukuda[1], R. Nagai[2] and E. Araki[1]

[1]*Department of Metabolic Medicine, Faculty of Life Sciences, Kumamoto University, Kumamoto, Kumamoto, Japan,* [2]*Laboratory of Food and Regulation Biology, Graduate School of Agriculture, Tokai University, Aso, Kumamoto, Japan,* [3]*Engineering Department 2, Product Development Center, New Business Development Division, SHARP Corporation, Tenri, Nara, Japan*

Background: Advanced glycation end products (AGEs) accumulate in tissues with a low turnover during a lifetime. It has been reported that the contents of the AGEs in skin predicts long-term diabetic complications in patients with diabetes, and skin fluorescence correlates with the AGEs content in skin. Although many studies investigated the skin fluorescence by detecting from forearms, this system had obvious limitation, such as interruption by content of melanin. We now developed the novel system by measuring skin fluorescence from fingertip without the interruption of melanin. In this study, we investigate whether our system contribute the assessment of skin AGEs content, and whether the intensity of skin fluorescence can predict the presence of diabetic microvascular complications in patients with type 2 diabetes.

Methods: Fluorescence data was recorded with a prototype consisting of a LED light source. Each fluorescence spectrum data was verified by a fluorescence spectrophotometer. Diabetic mice were prepared by injection with streptozotocin in C57BL/6 mice, and investigate for 6 weeks. Hemodialysis subjects ($n = 21$) and type 2 diabetic subjects ($n = 60$) were enrolled in the study, and skin fluorescence of all subjects was measured from the top of left middle finger.

Results: First, to determine the ideal wavelength for measuring AGEs, the skin fluorescence intensity was analyzed in hemodialysis subjects, who were well known to have high level of AGEs in skin tissues. Wavelength scanning showed that the highest intensity of fingertip fluorescence was obtained by an excitation maximum at 340 nm and emission maximum at 440 nm. Next, we investigated the auricle fluorescence level of control and diabetic mice. Compare to control mice, the intensity of fluorescence level in diabetic mice was dramatically increased. Therefore, we used above condition of wavelength to measure the skin fluorescence level for following study. We next investigated the fingertip fluorescence level of diabetic subjects. The fluorescence intensity in the subjects who have at least one microvascular complication ($n = 34$), including retinopathy, neuropathy or nephropathy, was significantly higher than that in the subjects without any microvascular complication ($n = 26$). Moreover, the fluorescence intensity was significantly increased in accordance with the increasing number of microvascular complications.

Conclusion: This study provides the first evidence that the intensity of fingertip fluorescence increased by the number of microvascular complications in patients with type 2 diabetes. This system may be valuable for predicting the presence of diabetic complications in subjects with diabetes.

### 0066

### Deteriorated glucose metabolism with a high-protein, low-carbohydrate diet in *db* mice, an animal model of type 2 diabetes, might be caused by insufficient insulin secretion

Behaviour and Nutrition

E. Arimura[1,2], W. Pilong[2], A. Marchianti[2], M. Nakakuma[2], M. Abe[2] and M. Horiuchi[2]

[1]*Major in Food and Nutrition, Department of Life and Environmental Science, Kagoshima Prefectual College, Kagoshima, Japan,* [2]*Department of Hygiene and Health Promotion Medicine, Graduate School of Medical and Dental Sciences, Kagoshima University, Kagoshima, Japan*

Objectives: We previously showed the deleterious effects of increased dietary protein on renal manifestations and glucose metabolism in leptin receptor-deficient (*db*) mice(Arimura E, et, al. Eur J Nutr 52:813–824,2013). Here, we further examined its effects on glucose metabolism, including urinary C-peptide. In addition, we orally administered the different mixtures, corresponding to low- or high-protein diets, to diabetic mice.

Methods: In diets experiments, under pair-feeding (equivalent energy and fat) using metabolic cage, mice were fed diets with different protein content [L-diet or H-diet: 12% or 24% protein, 71% or 59% carbohydrate, and 17% fat, respectively] for 15 days. In oral administration experiments, the respective mixtures [L-mixture or H-mixture: 12% or 24% proline, 71% or 59% carbohydrate (maltose or starch), and 17% linoleic acid, respectively] were supplied to mice. Biochemical parameters related to glucose metabolism were measured.

Results: The *db*-H-diet mice showed significantly higher water intake, urinary volume, and glucose levels than *db*-L-diet mice but exhibited similar levels of excreted urinary C-peptide. In contrast, control-H-diet mice showed significantly higher C-peptide excretion than control-L-diet mice. Both mice fed H-diet excreted high levels of urinary albumin. When maltose mixtures were administered, *db*-L-mixture mice showed significantly higher blood glucose after 30 min than *db*-H-mixture mice. However, *db*-H-mixture (composed of starch) mice showed significantly

© 2016 The Authors. Journal of Diabetes Investigation published by Asian Association for the Study of Diabetes (AASD) and John Wiley & Sons Australia, Ltd higher blood glucose during 120–300 min post-administration compared to *db*-L-mixture mice, although both groups exhibited similar insulin levels.

CONCLUSIONS: High-protein, low-carbohydrate diets deteriorated diabetic conditions and were associated with insufficient insulin secretion in *db* mice. Our findings may have implications for dietary management of diabetic symptoms in human patients.

### 0067
### The patients of type 2 diabetes mellitus on their pair monitoring care efficacy of the prio-post meal blood sugar

Behaviour and Nutrition

M.-Y. Tu and M.-Y. Liu
*Chi-Mei Medical Center, Yungkang City, Tainan, Taiwan*

OBJECTIVES: The study is which combine the health education activities with the "monitor pair of the pre-postprandial blood sugar." During hospitalization, dietitians counsel health education with subjects, teach them glycemic control techniques, and provides a low glycemic index diet concept with the design of the health dish, make some kind of nutritional intake and calorie estimate, detailed records on health logs, and the use of diabetes patients. Dietitians will exercise a healthy plate, teach patients how to squeeze the correct amount of the estimate.

METHODS: The qualifications in general internal medicine and endocrinology for diabetes are up to the physician. The execution time of the project is from the Republic of China on June 1st until November 30th, 2013. During hospitalization, subjects qualified are enclosed after being screening under the intervention conducted by a clinical dietitian nutrition counseling diabetes glycemic control techniques to teach health education and hospital outpatient clinic after at least three months.

RESULTS: The subjects in the program will be divided into two groups as the self-prepared one and the consulted + ordered group. After intervention, the study in the medical team showed that glycated hemoglobin value increased 40%, glycated hemoglobin values dropped 3.52%, while the other measurement of the blood glucose after each meal were statistically significant ($P < 0.0001$), up to the desired goal.

CONCLUSIONS: This result shows that the teachings of the subjects by pre-postprandial glucose pairing of monitoring go with the health of the log records can improve the glycemic control and upgrade the quality of subjects' life.

### 0068
### Graves' disease in type 1 diabetes

Thyroid and Osteoporosis

M. Hasebe, A. Sata, T. Yamada, K. Kusuki, K. Kiriyama, M. Kondo, Y. Tsurutani, M. Miyao and Y. Mizuno
*Kanto Central Hospital, Tokyo, Japan*

BACKGROUND: The prevalence of autoimmune diseases in patients with Type 1 diabetes mellitus (T1DM) is high. Among others, the autoimmune thyroid disease, most commonly Graves' disease (GD) is especially well known.

We observed that it is more difficult to achieve remission in patients with GD with T1DM compared to those without T1DM. Therefore, we examined the prevalence and clinical course of GD in T1DM at our hospital. We also examined whether there existed any relationship between anti-glutamic acid decarboxylase antibody (GADAb) and the clinical course of GD.

OBJECTIVES AND METHODS: Seventy-five T1DM patients (37 male, 38 female, aged 18–94 years) who attended our hospital from December 2008 to April 2015 were reviewed. We classified the patients into three groups; Acute onset type 1 diabetes mellitus (AT1DM, $n = 48$), slow progressive insulin dependent diabetes mellitus (SPIDDM, $n = 23$), and fulminant type 1 diabetes mellitus (FT1DM, $n = 4$).

We examined their TSH receptor antibody (TRAb), GADAb, and the presence of medical history related to GD retrospectively from their medical record.

RESULTS: TRAb was positive in 9.5% of all the T1DM patients. For the classified groups; 9.8% in AT1DM, 11.1% in SPIDDM, 0.0% in FT1DM were noted. Ten patients (13%) had GD (AIDDM $n = 5$, SPIDDM $n = 5$). Out of them, three patients (30%) had remission of GD, and one underwent radio isotope therapy. The remaining six patients are still under treatment with anti-thyroid drugs. The three patients who had remission were positive for GADAb. The two who had high titer of GADAb (1850, 870 U/mL) had no remission. No clear relationship was found between GADAb and clinical course of GD in our study.

ASSESSMENTS AND CONCLUSIONS: Only 30% of GD achieved remission in T1DM, while in general, the remission rate of GD is reported to be approximately 50%.

Our study shows that GD remission in T1DM patients may be more difficult to achieve compared to non-T1DM patients.

### 0069
### Vascular GLP-1 receptor expression decreases in subjects with obesity

Macrovascular Complications

T. Kimura[1], M. Shimoda[1], S. Okauchi[1], A. Obata[1], H. Hirukawa[1], K. Kohara[1], A. Hiraoka[2], K. Tamura[2], G. Chikazawa[2], H. Yoshitaka[2], I. Shimizu[2], K. Kaku[1] and H. Kaneto[1]
*[1]Kawasaki Medical School, Kurashiki, Okayama, Japan, [2]Sakakibara Heart Institute, Okayama, Okayama, Japan*

OBJECTIVES: It is known that incretin signaling improves vascular relaxation response in endothelial cells. In addition, it has been shown that incretin has anti-atherosclerotic effects in vascular smooth muscle cells. Although it has been reported that GLP-1 receptor expression in pancreatic β-cells is reduced under diabetic conditions, it remains unknown whether vascular GLP-1 receptor expression is altered under some conditions and which factor could influence vascular GLP-1 receptor expression. The aim of this study was to reveal the alteration of vascular GLP-1 receptor expression and the factors affecting its expression in human artery.

METHOD: Excised diseased artery was mechanically separated into the intima and tunica. Gene expression of various factors in the intima and tunica was analyzed by real time RT-PCR. Each gene expression was semi-quantified by the comparative Ct method with each result in β-actin as a control. Subjects were divided into obesity (BMI $\geq 25$ kg/m$^2$) and non-obesity group (BMI $<25$ kg/m$^2$) and comparison of the clinical parameters and various gene expression levels in the intima and tunica was performed.

RESULTS: Subjects were 40 patients (male / female = 27 / 13, abdominal or thoracic aortic aneurysm / aortic dissection / arteriosclerosis obliterans / other = 12 / 20 / 6 / 2, diabetic / non-diabetic = 11 / 26, age 68.0 ± 13.4 years old, BMI 23.8 ± 4.7 kg/m$^2$, HbA1c 6.2 ± 1.5%). BMI in obese and non-obese group was 28.6 ± 2.5 kg/m$^2$ and 20.7 ± 2.8 kg/m$^2$, respectively ($P < 0.05$). Between the obesity and non-obesity group, there was no significant difference in FPG (121.2 ± 32.2 mg/dL vs 116.6 ± 25.0 mg/dL). And there was no significant difference in HbA1c (5.9 ± 0.6% vs 6.4 ± 1.9%), triglyceride, HDL-cholesterol, LDL-cholesterol between the 2 groups. GLP-1 receptor gene expression in the intima and tunica was significantly lower in obesity group compared to that in non-obesity group ($P < 0.05$). In spearman's rank correlation coefficient test, there was significantly negative correlation between GLP-1 receptor gene expression and BMI, LDL-cholesterol in the intima and tunica. In addition, multiple regression analysis showed that only BMI had a significant negative correlation with GLP-1 receptor gene expression in intima ($P = 0.0063$).

CONCLUSIONS: GLP-1 receptor gene expression in human artery was down-regulated in subjects with obesity. The present results suggest that obesity and hypercholesterolemia down-regulate GLP-1 receptor gene expression in human artery.

### 0070
### Maternal and neonatal outcomes in Korean women with type 1 and type 2 diabetes

Epidemiology

S.-H. Kim[1], S. H. Yu[2], H. Y. Chung[3] and H.-S. Kim[4]
*[1]Dankook University, Seoul, Republic of Korea, [2]Hallym University, Chunchun, Republic of Korea, [3]Kyung Hee University, Seoul, Republic of Korea, [4]Dongnam Health College, Suwon, Republic of Korea*

The purpose of this study was to evaluate maternal and neonatal outcomes in Korean women with type 1 diabetes and type 2 diabetes. We performed a retrospective survey of 163 pregnancies in women with type 1 diabetes ($n = 13$) and

© 2016 The Authors. Journal of Diabetes Investigation published by
Asian Association for the Study of Diabetes (AASD) and John Wiley & Sons Australia, Ltd type 2 diabetes ($n$ = 150) treated from 2003 to 2010 at Cheil General Hospital, Korea. We compared maternal characteristics as well as maternal and neonatal outcomes between groups. Differences in HbA1c between type 1 and type 2 diabetes were not significant. Birth weight (3,501 ± 689.6 g vs. 3,366 ± 531.4 g) and rate of major congenital malformations (7.7% vs. 5.6%) were not significantly different. However, women with type 1 diabetes had higher rates of preeclampsia (38.5% vs. 8.2%, $P$ = 0.006), LGA (46.2% vs. 20.4%, $P$ = 0.004), macrosomia (38.5% vs. 13.4%, $P$ = 0.032), and admission for neonatal care (41.7% *vs.* 14.8%, $P$ = 0.03) than women with type 2 diabetes. Maternal and neonatal outcomes for women with type 1 diabetes were poorer than for women with type 2 diabetes, especially preeclampsia, LGA, macrosomia and admission to the neonatal intensive care unit.

### 0071

### Spexin as a satiety factor in fish model: insulin as a functional link between feeding and peripheral and central spexin expression through activation of PI3k/Akt and P38 MAPK Cascades

Insulin signaling/action

A. Ma, M. He, W. K. W. Ko and A. O. L. Wong

*School of Biological Sciences, The University of Hong Kong, Hong Kong*

Objectives: Spexin (SPX), a novel neuropeptide discovered by bioinformatics approach, has been recently identified as a satiety factor in fish model via differential regulation of orexigneic and anorexigenic signals within the central nervous system. However, it is still unclear regarding (i) the link between feeding and SPX expression, (ii) the role of SPX signals expressed in the periphery in feeding control, and (iii) the signal transduction mechanisms for SPX expression in target tissues. To address these questions, we used goldfish as a model to examine the functional role and post-receptor signaling of insulin as a postprandial signal to induce SPX gene expression both in the liver and brain of a fish model.

Methods: Whole animal experiments was used to examine the effects of feeding on plasma SPX, insulin and glucose levels and SPX mRNA expression in the liver and selected brain areas of the goldfish. The results obtained were also confirmed by intraperitoneal (IP) injection of glucose and insulin, respectively. To study the post-receptor signaling mediating insulin-induced SPX gene expression, a pharmacological approach coupled to Western blot to probe the activation status of target kinases was used in goldfish hepatocyte and brain cell cultures, respectively.

Results: Feeding in goldfish was effective in increasing plasma levels of glucose, insulin and SPX with parallel rise in SPX mRNA expression in the liver. Elevation in SPX mRNA levels was also observed in the liver as well as in brain areas involved in feeding control in goldfish with IP injection of glucose and insulin, respectively. In goldfish hepatocytes, SPX mRNA expression was up-regulated by insulin treatment and the stimulatory effect could be negated by blocking insulin receptor and occurred with rapid phosphorylation of $MEK_{1/2}$, $ERK_{1/2}$, P38 $^{MAPK,}$ PI3K and Akt. Insulin-induced SPX gene expression, interestingly, was found to be sensitive to the blockade of PI3K, Akt and P38 $^{MAPK}$, but not $MEK_{1/2}$ and $ERK_{1/2}$. Similar results were also obtained by repeating the experiments in goldfish brain cell culture.

Conclusions: These findings provide evidence that (i) feeding could trigger SPX signals in the periphery in fish model via up-regulation of SPX gene expression in the liver and increasing SPX levels in circulation, (ii) glucose-induced insulin secretion might serve as an endocrine signal linking feeding with SPX expression both in the central as well as in the periphery, and (iii) insulin-induced SPX gene expression were mediated by P38 $^{MAPK}$ and PI3K/Akt cascades at the tissue level.

### 0072

### Alpha-glucosidase inhibitor is suitable as combination therapy of SGLT2 inhibitor in Japanese patients with type 2 diabetes: hypothesis

Therapeutics of Diabetes

Y. Suzuki

*HDC Atlas Clinic, Tokyo, Japan*

According to open-label study of dapagliflozin (1), three drugs reduced HbA1c than other oral hypoglycemic agents or GLP1 injection therapies. One is alpha-glucosedase inhibitor (aGI), the other is glinide, and the third is pioglitazone.

The reason why alpha-GI(aGI) is so effective has not been clarified. SGLT2i is characterized as having effect of lowing and stabilizing fasting blood glucose. However, patients are likely to have compensatory over-eating (COE) (2). Therefore, we speculate that postprandial hyperglycemia in the morning is emphasized.

According to the phase III study of luseogliflozin (3) even though blood glucose curve of SGLT2i after eating meal is lower than that of placebo curve, hyperinsulinemia remains. Therefore, when miglitol is added, we hypothesize that the hyperinsulinemic state will be alleviated to normal state, thereby leading to normal insulin secretion curve and helping to reduce insulin resistance.

In addition, aGI, especially, miglitol, has more beneficial effect practically. It has a potential effect to induce mild diarrhea for some persons. SGLT2i, especially the product which has high selectivity to SGLT2 but not SGLT1, has potential effect to have constipation. Therefore, miglitol theoretically alleviates the tendency of constipation of high selective SGLT2i.

Furthermore at our clinic, the complaints of gas, which is the side effect of aGI, seems to be reduced. We hypothesized that, because patients who received SGLT2i therapy are likely to go toilet frequently due to polyuria, they can fart in the toilet at the same time. This time synergistic effect can let the patients not to suffer from the social trouble of fart, thereby making this adverse effect to be felt not so severe troublesome.

Thus, after SGLT2i treatment, miglitol has gained more attractive effect, by having the synergistic effect to suppress the postprandial hyperglycemia in the morning and by alleviating the constipation, with less worry about the trouble of fart.

References

1. Kaku K, et al. Dapagliflozin as monotherapy or combination therapy in Japanese patients with type 2 diabetes: an open-label study. Diabetes Ther. 5:415–33, 2014
2. Suzuki Y. Compensatory overeating (COE) could be a problem of diabetes diet education after sodium-glucose co-transporter 2 inhibitor treatment. The 6th international conf. on fixed combination of hypertension, dyslipidemia and diabetes, p18, 2015
3. Seino Y et al. Efficacy and safety of luseogliflozin as monotherapy in Japanese patients with type 2 diabetes mellitus: a randomized, double-blind, placebo-controlled, phase 3 study. Curr Med Res Opin. 30:1245–55. 2014.

### 0075

### A ratio of area under the C-peptide curve to area under the insulin curve in the first-phase insulin secretion during a hyperglycemic clamp correlates with the level of glucose tolerance

Integrated Physiology

N. Suematsu, H. Komada, K. Sakaguchi, T. Nakamura, T. Hamaguchi, A. Matsuoka, Y. Hirota and W. Ogawa

*Division of Diabetes and Endocrinology, Department of Internal Medicine, Kobe University School of Medicine, Kobe, Hyogo, Japan*

Objectives: Plasma insulin concentration is altered by both the secretion from the pancreas and the clearance in the liver. Thus, a plasma concentration ratio of C-peptide to insulin in steady state was used to be an index of hepatic insulin clearance. Hepatic clearance of insulin however reported to be constant during the rise of both insulin and C-peptide concentration in portal vein. The physiological meaning of the ratio of plasma C-peptide to insulin concentrations during non-steady state remains undetermined. We here investigated the significance of the ratio of C-peptide to insulin during changing insulin concentration.

Subjects and methods: A total of 134 subjects (NGT 63, IGT 21 and T2DM 50) were enrolled. A consecutive clamp (90-min hyperglycemic followed by 120-min hyperinsulinemic-euglycemic clamp) was performed for each participants. Then, a 75-g OGTT was conducted within 1 week after the clamp study. The area under the concentration curve (AUC) of C-peptide and that of insulin in the first-phase insulin secretion during hyperglycemic clamp were obtained to calculate the C-peptide to insulin ratio ($AUC_{C\text{-}peptide}$/ $AUC_{insulin}$). Fasting plasma glucose level (FPG), 2-h plasma glucose level (2 h-PG) and the AUC of plasma glucose levels ($AUC_{PG120}$) during OGTT were used for the indexes of glucose tolerance. The increment of the AUC in the first-phase insulin secretion during the hyperglycemic clamp (1st-PH) and AUC of insulin over a 90-min clamp period (total secretion) were used as indexes of glucose-stimulated insulin

© 2016 The Authors. Journal of Diabetes Investigation published by Asian Association for the Study of Diabetes (AASD) and John Wiley & Sons Australia, Ltd secretion. An insulin sensitivity index (ISI) was calculated as dividing glucose infusion rate at steady state by levels of both insulin and glucose at the end of hyperinsulinemic-euglycemic clamp. A clamp-based disposition index (clamp DI) was calculated as the product of 1st-PH and ISI. We investigated the relationships of $AUC_{C\text{-}peptide}/AUC_{insulin}$ with the indexes of glucose tolerance, insulin secretion, and insulin sensitivity ae well as with clamp DI.

RESULTS: $AUC_{C\text{-}peptide}/AUC_{insulin}$ was significantly correlated with fasting plasma glucose (FPG) ($r = 0.672$, $P < 0.001$), 2 h-PG ($r = 0.701$, $P < 0.001$) and $AUC_{PG120}$ ($r = 0.698$, $P < 0.001$). $AUC_{C\text{-}peptide}/AUC_{insulin}$ was also significantly correlated with 1st-PH ($r = -0.878$, $P < 0.001$), the total insulin secretion ($r = -0.815$, $P < 0.001$), and clamp DI ($r = -0.815$, $P < 0.001$). Furthermore, a weak correlation between the $AUC_{C\text{-}peptide}/AUC_{insulin}$ and the ISI ($r = -0.291$, $P = 0.001$) was observed.

CONCLUSION: The $AUC_{C\text{-}peptide}/AUC_{insulin}$ was consistently correlates with the level of glucose tolerance.

### 0076

### Fibroblast growth factor 23 is associated with the presence of coronary artery disease and the number of stenotic vessels

Macrovascular Complications

X. Hu[1], X. Ma[1], X. Pan[1], Y. Hao[1], Y. Luo[1], Z. Lu[2], Y. Bao[1] and W. Jia[1]

*[1]Department of Endocrinology and Metabolism, Shanghai Jiao Tong University Affiliated Sixth People's Hospital; Shanghai Clinical Center for Diabetes; Shanghai Key Clinical Center for Metabolic Disease;, Shang hai, China, [2]Department of Cardiology, Shanghai Jiao Tong University Affiliated Sixth People's Hospital, Shang hai, China*

OBJECTIVES: Fibroblast growth factor 23 (FGF23), a hormone-like fibroblast growth factor, has been reported to be involved in cardiovascular disease. The aim of this study was to investigate the association between serum FGF23 levels and the presence of coronary artery disease (CAD), as well as the number of stenotic vessels.

METHOD: A total of 254 eligible participants, including 167 men and 87 postmenopausal women were enrolled in this study. Coronary angiography was used for diagnosis of CAD. Serum intact FGF23 levels were determined by a two-sided sandwich enzyme-linked immunosorbent assay.

RESULTS: The median serum FGF23 levels of the entire study population were 39.9 (33.1–47.5) pg/mL. Serum FGF23 levels were higher in subjects with 1-vessel disease than those without CAD ($P < 0.05$), which further increased significantly in the subjects with multi-vessel disease compared to those with 1-vessel disease ($P < 0.05$). Serum FGF23 levels increased with cumulative number of stenotic vessels ($P$ for trend <0.001). Multiple stepwise regression analysis revealed estimated glomerular filtration rate (standardized $\beta = -0.298$; $P < 0.001$) and body mass index (standardized $\beta = 0.132$; $P = 0.049$) were independent factors correlated with serum FGF23 levels. Multivariate logistic regression analysis showed that serum FGF23 levels were positively and independently associated with the presence of CAD (odds ratio = 1.058, 95% confidence interval = 1.025–1.092; $P = 0.001$). Additionally, serum FGF23 levels were also correlated with multi-vessel disease significantly (odds ratio = 1.034, 95% confidence interval = 1.007–1.062; $P = 0.013$).

CONCLUSIONS: Serum FGF23 levels exhibit positive and independent associations with the presence of CAD and the cumulative number of stenotic vessels.

### 0077

### A small molecule that facilitates the differentiation into pancreatic endocrine cells from human ESCs/iPSCs

Islet Biology

Y. Kondo[1,2], T. Toyoda[2], M. Funato[2], S. Matsui[2], T. Sudo[2], X. Zhuang[1], A. Watanabe[2], A. Ohta[2], N. Inagaki[1] and K. Osafune[2]

*[1]Department of Diabetes, Endocrinology and Nutrition, Kyoto University, Kyoto, Japan, [2]Center for iPS Cell Research and Application (CiRA), Kyoto University, Kyoto, Japan*

Type 1 diabetes is characterized by absolute insulin deficiency state and persistent high levels of blood glucose. Although pancreas and pancreatic islet transplantation is established as the radical treatment of type 1 diabetes, problems of the transplantation therapies include shortage of donors and immune rejection. Generation of pancreatic β cells from embryonic stem cells (ESCs) or induced pluripotent stem cells (iPSCs) would lead to the development of a promising replacement therapy for insulin-dependent diabetes, such as type 1 diabetes. However, the induction of insulin-producing (INS(+)) cells from human ESCs/iPSCs is inefficient.

PURPOSE: To establish an efficient differentiation method of INS(+) cells from human ESCs/iPSCs by identifying novel small molecule inducers.

METHODS: Based on the previously-reported protocol (Kunisada Y et al., 2012), human iPSCs were induced into PDX1(+) pancreatic progenitors through SOX17(+) definitive endoderm, and then 4 factors (Nicotinamide, Forskolin, Dexamethasone and ALK5 inhibitor; 4Fs) and/or small molecules were added on the PDX1(+) cells. We used High-Throughput Screening (HTS) system to examine thousands of compounds from various libraries. Anti-insulin immunostaining was performed and the induction rate of INS(+) cells was quantitatively evaluated. Small molecules that more efficiently induce INS(+) cells than the previous protocol with 4Fs were defined as primary hits. We established an INSULIN-GFP human iPSC reporter line to monitor and isolate the INS(+) cells and evaluated the function and gene expression of INS(+) cells differentiated from human iPSCs. We elucidated the mechanisms of action of inducer compounds by RNA-sequencing analyses.

RESULTS: Out of about 1,250 small molecules that we screened, one hit compound was identified. When the hit compound was combined with the previous protocol with 4Fs, the induction rate of INS(+) cells was increased more than three-fold. We found that the hit compound facilitates the differentiation of endocrine cells including INS(+) cells both in vitro and ex vivo by expanding the NEUROG3(+) endocrine precursor cells. The INS(+) cells generated with the hit compound showed the ability to secrete C-peptide in vitro. We found that the mechanisms of action of the hit compound to induce the pancreatic endocrine differentiation include the inhibition of BMP4, which is identified by RNA-sequencing analyses of the differentiated cells induced by the hit compound.

CONCLUSION: We have identified one small molecule that facilitates the differentiation of human iPSCs/ESCs into pancreatic endocrine cells including insulin-producing β-like cells in vitro.

### 0079

### Polyneuropathy or neuropathic pain did not increase at pre-diabetc stage in Japanese population

Microvascular Complications

S. Kurisu[1,2], K.-I. Ogawa[1], H. Sasaki[1], H. Tanaka[1], M. Yamaneki[1], I. Nakanishi[1], H. Furuta[2], M. Nishi[2], K. Nanjo[3] and T. Akamizu[2]

*[1]Medicine, Kihoku Hospital, Katsuragi-Cho, Japan, [2]First Department of Medicine Wakayama Medical University, Wakayama, Japan, [3]Rosai Hospital, Wakayama, Japan*

OBJECTIVES: It is controversial whether polyneuropathy (PN) or neuropathic pain (NP) is occurred in the patients with mild impaired glucose tolerance (preDM) before the onset of diabetes. We examined the prevalence and risk factors of PN and NP in the regional population-based Japanese subjects (Katsuragi cohort) .

METHODS: Katsuragi cohort consisted of 387 cases (mean age 62.1 years), they received medical screening program of Katsuragi-Town. Known diabetic patents were excluded. They were divided into three groups according to the fasting plasma glucose (FPG) and HbA1c levels. 1) Normal group (N group: $n = 301$, HbA1c<6.0% and FPG <126 mg/dL), 2) newly diagnosed diabetic group (NDM group: $n = 15$, HbA1c = >6.5% and/or FPG = >126 mg/dL), 3) preDM group (preDM group; $n = 71$, cases who did not correspond to N and NDM groups). PN was diagnosed according to the criterion of probable diabetic neuropathy in Toronto consensus. Namely, two or more positive findings of the following three abnormalities was judged as PN, 1) decreased Achilles tendon reflexes (ATR), 2) impaired vibratory perception (VP) at the feet and 3) neuropathic symptoms in the feet. NP was defined by the presence of pain and/or tingling sensation in the feet accompanying with decreased ATRs and/or impaired VP. Prevalence of these findings was statistically analyzed by chi-square test between groups. Risk factors of PN and NP were analyzed using multiple logistic regression analysis.

© 2016 The Authors. Journal of Diabetes Investigation published by Asian Association for the Study of Diabetes (AASD) and John Wiley & Sons Australia, Ltd

Results: Averaged HbA1c (%) and body mass index (BMI) in N, preDM and NDM groups were 5.44, 5.72 and 6.47, and 22.2, 24.1 and 23.6, respectively. The prevalence (%) of PN in N, preDM and NDM were 5.1, 8.5 and 26.7. A significant increase was observed in NDM group ($P$ = 0.003). The prevalence (%) of NP in N, preDM and NDM were 1.7, 4.2 and 6.6, respectively. NP did not show a significant increase. The significant risk factors of PN were age, glucose tolerance and the carotid artery plaque. The significant risk factor of NP was age and dyslipidemia has been extracted as a protective factor.

Conclusions: Prevalence of PN or NP mildly elevated in pre-diabetic stage compared to normal subjects. But the difference was not statistically significant. Although the prevalence of PN increased significantly in newly diagnosed diabetic stage, the prevalence of NP did not increased significantly in newly diagnosed diabetic stage. Clinical risk factors of PN were age, glucose intolerance and the carotid artery atherosclerotic change. In Japanese type 2 diabetic patients, obesity was not a risk factor for PN in contrast to Caucasians.

### 0080

### Limb salvage achievement by maggot debridement therapy (MDT); the analysis of 164 patients with chronic refractory foot gangrene

Microvascular Complications

S. Kirinoki-Ichikawa[1], M. Miyamoto[1], G. Takagi[1], Y. Kubota[1], A. Tezuka[1], T. Iimura[2], R. Ogawa[2] and W. Shimizu[1]

[1]*Department of Cardiology, Nippon Medical School Hospital, Tokyo, Japan,* [2]*Department of Plastic Reconstructive Surgery and Aesthetic Surgery, Nippon Medical School Hospital, Tokyo, Japan*

Background: Diabetic gangrene is considered to be a refractory wound and a major cause of amputation, especially for diabetics with comorbid peripheral arterial disease (PAD), whose gangrene is more highly infected, ischemic, painful, and who generally also suffer malnutrition. Those adverse conditions often make surgical debridement resulting in aggravation of necrosis. Maggot debridement therapy (MDT) is considered one of the effective treatments for chronic wound, due to its non-invasive debridement, and anti-bacterial- and granulation power. Furthermore, MDT is easy and simple to handle for internal medicine doctors and nurses. We have employed maggot debridement therapy over one hundred eighty times and used thirty-eight thousand maggots over a decade. Based on this abundant experience, we have found that internal medicine specialists' involvement and 'coordination' in wound care are essential to successfully treat diabetic gangrene, more so than leaving wound treatment up to surgeons alone.

Objective: We performed MDT since 2004 on admission to the patients with previously diagnosed that major lower limb amputation was the only option in other hospitals, in order to eliminate methicillin-resistant Staphylococcus aureus (MRSA) and other bacterial pathogens, followed by revascularization technique, hyperbaric oxygen therapy, and other multidisciplinary therapies. We reviewed the effectiveness of MDT based on the outcome (limb salvage and life survival) of post-MDT patient analysis.

Methods: The 164 patients' survival period and limb survival period after MDT were reviewed. In addition, risk factors such as influence of MRSA on wound surface, DM, PAD and hemodialysis to the outcome was also analyzed.

Result: Average follow-up period was 7.1 $\pm$ 0.2 years. Univariate analyses showed the comorbidity of MRSA infection, PAD and hemodialysis were adverse prognostic factors ($P < 0.01$, log-rank test). Multivariate analyses by Cox regression analysis showed the independent risk factor for leg amputation was hemodialysis ($P$ = 0.001, OR 8.77). Limb salvage rate was 92.6% and 7-year survival rate was 91.4%. Given that previous articles indicate a 5-year survival rate of approximately 40% after ischemic limb amputation, it is certain that limb salvage and preserved ambulatory ability can improve not only patients' QOL but survival itself.

Conclusion: MDT is an effective treatment for severe chronic wound accompanied by multiple-drug-resistant bacteria, and it improved patient prognosis and prevented limb amputation.

### 0081

### A case of type 2 diabetes whose symptoms of carpal tunnel syndrome improved after taking SGLT2 inhibitors: especially tofogliflozin

Therapeutics of Diabetes

Y. Suzuki

*HDC Atlas Clinic, Tokyo, Japan*

Carpal tunnel syndrome (CTS) is more prevalent in diabetic patients than in non-diabetic population. Sodium-glucose co-transporter 2 inhibitor (SGLT2i) is a medication for glycemic control. We experienced a patient of type 2 diabetes, who had an interesting episode of the efficacy of SGLT2i on CTS symptoms. Her symptoms of CTS improved dramatically after SGLT2i treatment during spring and end of summer (from Apr 2014 till Nov 2014), while they recurred in autumn (from Nov 2014).

Sixty-five years of old, female, 154.4 cm height, 57.4 kg body weight (BMI: 24.2), was diagnosed to have type 2 diabetes at age 55. She had been treated with sitagliptin 100 mg/day with moderate glycemic control. HbA1c was around 6.7% before SGLT2i treatment.

Definite diagnosis of CTS was made two years ago by her manifestation of Tinel's sign, Phalen maneuver, hand pain, tingling, numbness and sleep disturbances. Because the symptoms were not relieved for long time, she was recommended surgical intervention for her hands. However, after SGLT2i medication (either of ipragliflozin 50 mg/day, luseogliflozin 2.5 mg/day, tofogliflozin 20 mg/day, or dapagliflozin 5 mg/day) during from spring to end of summer (from 23th Apr 2014 till 15th Nov 2014), symptoms of CTS (such as pain and numbness) in both hands and sleep disturbance have improved dramatically and disappeared.

As for the mechanism, we speculate that SGLT2i has a beneficial effect by reducing the body fluid volume, thereby releasing the pressure on the strangulated nerves to alleviate the CTS symptoms.

Interestingly, among the several SGLT2i, the patient preferred to take tofogliflozin rather than other SGLT2is. Tofogliflozin has a strong effect during the day time because the biological half-life is shorter than other SGLT2i (such as ipragliflozin, dapagliflozin, luseogliflozin, canagliflozin, and empagliflozin). The patient changed the SGLT2i each other and one by one. Finally, she decided to chose the medication because tofogliflozin has the most strong effect among the SGLT2is during the day time and because the symptoms of CTS gets severe during the day time.

In conclusion, although several SGLT2is seem not to be different, the strong effect during the day time is different depending on the short half-time. In addition, we propose that SGLT2i can be one of the choices for the oral conservative treatment of CTS with diabetes. Although other conservative treatment of CTS, such as steroids or diuretic produce side-effects like induction of hyperglycemia, SGLT2i such as tofogliflozin has a big advantage without side effect to glycemic control.

### 0082

### *Nidd6/of* QTL includes diabetogenic genes associated with obesity using new animal models in rats

Genetics and Epigenetics

J. Koto, D. Sasaki, R. Watadani and K. Matsumoto

*Kyoto Sangyo University, Motoyama, Kamigamo, Kita-ku, Kyoto-city, Japan*

Recently, it is well understood that the interactions between obesity and genetic predisposition are likely to be important in the etiology of type 2 diabetes. However little is known how obesity induces the onset of type 2 diabetes. Therefore, identification of causative genes for diabetes associated with obesity is necessary for elucidation of the mechanism of the onset of type 2 diabetes using new obese animal models. There are 14 diabetogenic loci are identified by QTL analysis of OLETF rats. But it is not clear which locus is associated with obesity. In this study, we investigated whether *Nidd6* QTL region relates to hyperglycemia and insulin resistance.

We used F344, sigle congenic introgressed *Nidd6* QTL region into F344 (F344-nidd6), single obese congenic introgressed Lepr-/- locus into F344 (F344-fa), and double congenic rats that have homozygotes for *Nidd6* and *Lepr-/-* locus

© 2016 The Authors. Journal of Diabetes Investigation published by Asian Association for the Study of Diabetes (AASD) and John Wiley & Sons Australia, Ltd

(F344-nidd6-fa) rats. We carried out OGTT to analyze blood glucose and insulin levels. Liver was dissected at fasting and postprandial period. RNA levels were determined using reverse transcribed and quantitative RT-PCR. Analysis of DNA sequence was performed.

Double congenic rats (F344-nidd6-fa) showed significantly higher blood glucose levels than those of obese control rats (F344-fa) at postprandial period. This phenotype was observed specifically in the obese strains. This result indicates that the *Nidd6* region is responsible for the onset of type 2 diabetes under condition of obesity. *Nidd6* region contains 60 genes. Therefore, we investigated genes expression of *Nidd6* region in the livers of these rats using Real Time PCR. We finally found five candidate genes for the onset of type 2 diabetes associated with obesity. There was no difference in their DNA sequences between double congenic rats and obese control rats about exons, 1000 base pairs on the 5′ upstream, and 3′utr of three candidate gene. Since double congenic rats showed significantly higher expression levels of G6Pase than those of obese control rats at postprandial period, a gene for a pathway of glycogenolysis may correlate to the hyperglycemia in the double congenic rats.

Our data showed that *Nidd6* QTL region includes a gene responsible for hyperglycemia, though further works are necessary to identify the causative gene (s).

### 0083

### Insulin lispro low mixture twice daily versus basal insulin glargine and prandial insulin lispro once daily in east asian and caucasian Type 2 diabetes mellitus patients

Therapeutics of Diabetes

T. Lew (Presenter Only)[1], J. H. Han[2], D. Edralin[3], R. Duan[4] and A. Rodriguez[5]
[1]*Presenting on behalf of Eli Lilly and Company, Indianapolis, IN, USA,* [2]*Lilly Korea Ltd., Seoul, Republic of Korea,* [3]*Eli Lilly, Pasig City, The Philippines,* [4]*Eli Lilly and Company, Indianapolis, IN, USA,* [5]*Lilly Spain, Alcobendas, Spain*

Objectives: This post-hoc analysis described the efficacy and safety of insulin lispro low mixture (LM) twice daily versus bedtime insulin glargine plus prandial insulin lispro (IGL) administered once daily before the largest meal in East Asian (EA) and Caucasian type 2 diabetes mellitus patients who previously failed to reach glycemic targets on basal insulin glargine with metformin and/or pioglitazone.

Methods: This phase 4, randomized, open-label, multinational, multicenter trial included patients with glycosylated hemoglobin (HbA1c) between ≥7.5% and ≤10.5%, and fasting plasma glucose ≤6.7 mmol/L.

Results: Baseline mean (SD) HbA1c values were numerically similar between groups in EA ($N$ = 79) and Caucasian ($N$ = 278) patients (EA: LM = 8.78 [0.71] vs IGL = 8.72 [0.90]; Caucasian: LM = 8.56 [0.78] vs IGL = 8.51 [0.70]). Mean (SD) HbA1c significantly ($P$ < 0.001) decreased from baseline to 24 weeks for both treatments in both subpopulations (EA: LM = −1.32% [0.96] and IGL = −0.89% [0.98]; Caucasian: LM = −1.24% [0.98] and IGL = −1.05% [0.97]). The proportion reaching HbA1c ≤7% at week 24 was LM = 33.3% and IGL = 22.9% for EA patients, and LM = 37.2% and IGL = 34.1% for Caucasian patients. Mean (SD) rate of hypoglycemia per 30 days was LM = 0.74 (1.16) and IGL = 1.22 (1.36) in EA patients, and LM = 1.38 (2.04) and IGL = 1.65 (2.43) in Caucasian patients. Mean (SD) change in weight gain was LM = 0.62 kg (2.78) and IGL = 0.51 kg (2.63) in EA patients, and LM = 1.77 kg (2.91) and IGL = 0.67 kg (3.09) in Caucasian patients.

Conclusions: Improved glycemic control was observed in EA and Caucasian patients treated with either LM or IGL.

### 0084

### Efficacy and safety of once-weekly dulaglutide versus insulin glargine in combination with metformin and/or a sulfonylurea in predominantly Asian patients with type 2 diabetes

Therapeutics of Diabetes

T. Lew (Presenter Only)[1], G. Ning[2], L. N. Ruiz[3], E. Filippova[4], K. H. Song[5], P. Li[6] and J. Yang[6]
[1]*Eli Lilly and Company, Indianapolis, IN, USA,* [2]*Rui Jin Hospital, Shanghai, China,* [3]*Investigación en Salud y Metabolismo S.C. Hospital CIMA, Chihuahua, Mexico,* [4]*Medinet, LLC, St. Petersburg, Russia,* [5]*Yeouido St. Mary's Hospital, Seoul, Republic of Korea,* [6]*Lilly Suzhou Pharmaceutical Company, Ltd, Shanghai, China*

Objectives: This randomized, open-label, multinational, parallel-arm study compared the efficacy and safety of once-weekly glucagon-like peptide-1 (GLP-1) receptor agonist dulaglutide (DU) to once-daily insulin glargine in patients with type 2 diabetes (T2D) uncontrolled on metformin (MET) and/or a sulfonylurea (SU).

Methods: A total of 789 patients (83.9% Asian; mean HbA1c [A1C], 8.36%; mean weight, 73.8 kg) were randomized (1:1:1) to DU 1.5 mg, DU 0.75 mg, or glargine. The primary and key secondary objectives were to test the noninferiority (margin = 0.4%) and superiority of DU 1.5 mg and 0.75 mg vs insulin glargine using a prespecified gatekeeping method, as measured by A1C change from baseline at Week 26.

Results: Both DU 1.5 mg and 0.75 mg demonstrated superiority to glargine (LS mean difference [%] [95% CI]: −0.55 [−0.71, −0.38] and −0.17 [−0.34, −0.00], respectively). More patients taking DU achieved A1C target <7.0% than those taking glargine: DU 1.5 mg (64.7%; 2-sided $P$ < 0.001 vs glargine), DU 0.75 mg (53.5%; 2-sided $P$ < 0.05 vs glargine), and glargine (40.6%). Weight (kg) decreased with DU 1.5 mg and 0.75 mg (LS mean [SE]: −1.51 [0.197], −0.88 [0.197]; 2-sided $P$ < 0.05 vs glargine) and increased with glargine (0.96 [0.197]). Total hypoglycemia incidence was lower in DU 1.5 mg and 0.75 mg (17.5% and 16.0%; 2-sided $P$ < 0.05 vs glargine) than glargine (26.8%). No severe hypoglycemia was reported. Diarrhea and nausea were more common with DU 1.5 mg (15.2%, 8.7%) and DU 0.75 mg (8.4%, 4.9%) vs glargine (1.6%, 0.8%).

Conclusions: Once-weekly dulaglutide is an effective and safe alternative to once-daily insulin glargine in patients with T2D taking MET and/or a SU who fail to achieve optimal glycemic control.

### 0085

### Administration of SGLT-2 inhibitor ipragliflozin improves insulin resistance, insulin secretion and reduces body weight and body fat mass in obese Japanese patients with type 2 diabetes mellitus

Clinical Diabetes

Y. Matsuhashi, S. Chikazawa, H. Otaka, K. Takahashi, K. Makita, A. Kanba, K. Matsuki, M. Yanagimachi, H. Murakami and M. Daimon
*Hirosaki University Graduate School of Medicine, Hirosaki, Aomori, Japan*

Objectives: We evaluated the efficacy of ipragliflozin(IPRA), a SGLT-2 inhibitor, on glycemic control, blood pressure(BP), body weight(BW) and body compositions when added to obese Japanese subjects with type2 DM (T2DM).

Method: Obese subjects with T2DM (BMI >25.0 kg/m$^2$) were enrolled ($n$ = 22); Diet ($n$ = 1), OADs ($n$ = 8), insulin ($n$ = 12) and GLP-1RA therapy ($n$ = 1). HbA1c, glycated albumin(GA), fasting plasma glucose (FPG), insulin(IRI), C-peptide(CPR) and glucagon were measured. Index of insulin resistance (HOMA-R) and insulin secretion (SUIT index) were calculated as follows; HOMA-R = IRI*FPG/405, SUIT index = 1485*CPR/(FPG-61.8). BP were checked at the outpatient clinic. BW and body compositions (body fat mass: BFM, lean body mass: LBM, total body water: TBW, protein and minerals) were analysed using the InBody770. IPRA was added without altering previous therapy. Differences in each parameters were evaluated with repeated-measure ANOVA. Changes of HbA1c and GA from baseline to 12 weeks (ΔHbA1c and ΔGA) were calculated. $P$ < 0.05 was defined as statistically significant. Data was mean ± standard deviation (SD).

© 2016 The Authors. Journal of Diabetes Investigation published by
Asian Association for the Study of Diabetes (AASD) and John Wiley & Sons Australia, Ltd

RESULTS: The IPRA dosage was 30.1 ± 10.9 mg initially and increased to 47.6 ± 7.5 mg ($P < 0.05$). HbA1c and GA decreased from 8.47 ± 1.59% to 7.55 ± 1.06% and from 20.2 ± 5.8% to 17.4 ± 4.1%, respectively ($P < 0.05$). FPG decreased from 171.4 ± 44.0 mg/dL to 140.9 ± 42.3 mg/dL ($P < 0.05$). HOMA-R decreased from 5.7 ± 5.2 to 4.3 ± 4.0 ($P < 0.05$), and SUIT index increased from 43.8 ± 37.2 to 57.0 ± 43.2 ($P < 0.05$). Systolic BP lowered from 140.0 ± 21.4 mmHg to 132.7 ± 18.6 mmHg ($P < 0.05$). BW decreased from 85.1 ± 14.1 kg to 82.3 ± 14.0 kg ($P < 0.05$). BFM decreased from 33.7 ± 9.5 kg to 31.7 ± 10.0 kg ($P < 0.05$). LBM also decreased significantly; however, such decrease appear to be due to the decrease in TBW (37.8 ± 8.0 L to 37.0 ± 7.5 L, $P < 0.05$), which is the most major component of LBM. BFM showed decrease over time with significant difference; whereas, the loss of TBW remained at the same level after IPRA therapy. Simple regression analysis showed that ΔGA was correlated not with ΔBW and ΔTBW, but with ΔBFM (r = 0.62, $P < 0.05$). ΔHbA1c had a weak association with ΔBFM (r = 0.39, $P = 0.19$).

CONCLUSIONS: Our data indicated that IPRA improved glycemic control, insulin resistance, insulin secretion, BP and BW. Both BFM and TBW were main factors responsible for BW reduction. BFM reduction was associated with improvement of glycemic control.

### 0087

### Inhibition of insulin secretion by adrenaline is mediated by change in activity of nonselective cation channel TRPM2

Islet Biology

K. Ito[1], M. Yoshida[1], H. Yamada[1], K. Dezaki[2], S. Ookawara[1], K. Tabei[1], M. Kawakami[1], Y. Morishita[1], T. Yada[2] and M. Kakei[1]

*[1]Saitama Medical Center, Jichi Medical University, Saitama, Japan, [2]Depertment of Integrative Physiology, Jichi Medical University, Shimotsuke, Japan*

OBJECTIVE: Adrenaline receptors are expressed in pancreatic beta-cells. Adrenaline (Adr) suppresses glucose-stimulated insulin secretion (GSIS) via Gi-coupled receptor with reduction of cyclic AMP production. However, it is suggested adrenaline may act on other sites for inhibition of insulin secretion. Recently, we have reported that incretin hormones and glucose over a threshold concentration of GSIS activate a class of nonselective cation channels (NSCCs) in additive manner and thereby enhancing GSIS. The major mechanism of the activation of NSCC is via cAMP-Epac-TRPM2 pathway. The aim of the present study is to examine whether inhibition of GSIS by adrenaline involves interactions with NSCC including TRP channel.

METHOD: Islets of Langerhans and pancreatic beta-cells were isolated from C57BLB6 mouse. Measurement of insulin secretion was performed by using static incubation and determined with ELISA-kit. Perforated whole-cell clamped currents were recorded at the holding potential of −70 mV in the presence of 100 μm tolbutamide.

RESULTS: Glucose at 16.7 mM (16.7 mMG) increased GSIS compared with 2.8 mMG ($P < 0.001$), and 100 pM exendin-4 (Ex4), 1 mM dibutyryl cAMP (dbcAMP) and 10 μM Epac activator (8-pCPT-AM) at 16.7 mMG additionally increased GSIS ($P < 0.05$) as compared to GSIS at 16.7 mMG alone. Similarly 16.7 mG increased NSCC current, and in the presence of 5.6 mMG 100 pM Ex4, 1 mM dbcAMP and 10 μM 8-pCPT-AM increased NSCC current ($P < 0.05$) as compared to those at 5.6 mMG alone. Furthermore, these agents induced the membrane depolarization and addition of 5 μM adrenaline hyperpolarized the membrane ($P < 0.05$ vs control). The increases in GSIS and NSCC current by glucose increase or the agents used above were suppressed by exposure to 5 μM adrenaline. Similarly NSCC currents was decreased from −2.15 ± 0.28 pA/pF (16.7 mMG) to −1.14 ± 0.34 pA/pF (16.7 mMG with Adr), $P < 0.05$; from −2.58 ± 0.70 pA/pF (100 pM Ex4) to −0.63 ± 0.09 pA/pF (100 pM Ex4 with Adr), $P < 0.05$; from −2.97 ± 0.40 pA/pF (dbcAMP) to −1.16 ± 0.25 pA/pF (dbcAMP with Adr), $P < 0.01$ and from −1.68 ± 0.19 pA/pF (8pCPT) to −0.68 ± 0.11 pA/pF (8pCPT with Adr), $P < 0.01$.

CONCLUSION: Adrenaline suppresses GSIS via in part inhibition of NSCC current and consequent hyperpolarization. It is suggested that mechanistic pathways are via Gi-coupled receptor binding with following reduction of cAMP production and an unidentified site located downstream of Epac activation before TRPM2 channel.

### 0088

### *Wfs1*-deficiency causes beta-cell dedifferentiation associated with enhanced er stress and oxidative stress, independently of hyperglycemia

Islet Biology

K. Amo-Shinoki[1], K. Tanabe[1,2], M. Hatanaka[1,2] and Y. Tanizawa[1,2]

*[1]Yamaguchi University Graduate school of medicine, Ube, Yamaguchi, Japan, [2]Ymaguchi University Hospital, Ube, Yamaguchi, Japan*

Wolfram syndrome is an autosomal recessive disorder characterized by juvenile-onset insulin-dependent diabetes mellitus, and is caused by mutations in the *WFS1* gene, which has been also known as a type 2 diabetes susceptibility gene. The *Wfs1* deficient (*Wfs1*$^{-/-}$) mice exhibit a selective beta-cell loss and impaired insulin secretion. This phenotype has been thought to be associated with an augmentation of ER stress. However, the underlying mechanisms remain unknown. The recent reports have suggested that the loss of insulin producing beta-cell in diabetes results from not only apoptosis but also dedifferentiation. In the present study, we investigated whether loss of beta-cell maturity is associated with beta--cell failure in *Wfs1*$^{-/-}$ mice. We analyzed change in expression of the key transcriptional factors maintaining islet maturity in addition to various stress responsive molecules in islets of *Wfs1*$^{-/-}$ mice. Islets of 18 weeks of age of *Wfs1*$^{-/-}$ mice, in which blood glucose levels were still maintained, exhibited elevated gene expression of ER stress- and oxidative stress-responsive genes such as CHOP, Bip, spliced XBP-1, superoxide dismutase 1 and thioredoxin reductase 1, relative to those in islets of wild type (WT) mice. At the same time, a robust reduction of MafA gene expression and a more than three-fold increase in Neurogenin3 gene expression were observed in *Wfs1*$^{-/-}$ mice relative to WT mice. Immunohistochemistry on pancreatic sections from *Wfs1*$^{-/-}$ mice showed that beta cells were severely decreased and that residual beta cells were weakly stained with insulin. Consistent with change in gene expression, MafA-expressing cells were markedly reduced. In contrast to that, some of islet cells with nuclei stained by Neurogenin3 appeared in *Wfs1*$^{-/-}$ islets. These findings were more apparent in Islets of *Wfs1*$^{-/-}$*A*$^{y}$/*a* mice in which beta-cell loss was accelerated by the addition of peripheral insulin resistance. At the same time, despite apparent loss of insulin-positive cells, only slight increase in dying insulin positive-cells, assessed by TUNEL staining, could be seen in islets of both *Wfs1*$^{-/-}$and *Wfs1*$^{-/-}$*A*$^{y}$/*a* mice relative to WT mice. Therefore, these results suggested that *Wfs1*-deficiency brought about beta-cell dedifferentiation, contributing to loss of functional beta cell.

### 0090

### Pancreatic volume is reduced in latent autoimmune diabetes in adults (LADA)

Clinical Diabetes

J. Lu[1,2], X. Hou[2], L. Zhang[2], C. Hu[2], J. Zhao[3], Y. Bao[2] and W. Jia[2]

*[1]Department of Endocrinology and Metabolism, Fengxian District Center Hospital, Shanghai, China, [2]Department of Endocrinology and Metabolism, Shanghai Jiao Tong University Affiliated Sixth People's Hospital, Shanghai, China, [3]Department of Radiology, Shanghai Jiao Tong University Affiliated Sixth People's Hospital, Shanghai, China*

AIM: Till now, the pancreatic volume in latent autoimmune diabetes in adults (LADA) has not been reported. We aimed to investigate the pancreatic volume (PV, cm$^3$) and its clinical significance in LADA patients.

METHODS: Patients with LADA ($n = 68$) and type 1 diabetes ($n = 58$) were recruited in the out-patient department between May and July in 2013. 71 inpatients with type 2 diabetes and 87 non-diabetic controls were selected from inpatients admitted to the one hospital during the same period. Abdominal computerized tomography scan was performed on each patient and PV was calculated.

RESULTS: The medians of PV in descending order were non-diabetic controls (68.6 cm$^3$), LADA (56.2 cm$^3$), type 2 diabetes (55.8 cm$^3$), and type 1 diabetes group (43.0 cm$^3$). The PV in LADA was comparable to that in type 2 diabetes but significantly greater than that in type 1 diabetes. PV was significantly correlated with gender, waist circumstance, BMI, diastolic blood pressure, HDL-C and body surface area in LADA patients (all $P < 0.05$). The correlation between PV and fasting C-peptide was high in LADA (r = 0.643, $P < 0.001$) and moderate in type 2 diabetes (r = 0.467, $P < 0.001$). The area under the ROC curve for PV

© 2016 The Authors. Journal of Diabetes Investigation published by Asian Association for the Study of Diabetes (AASD) and John Wiley & Sons Australia, Ltd predictive of absolute insulin deficiency (FCP <0.9 ng/mL) was 0.85 (0.76–0.94) in patients with LADA.

Conclusion: PV was significantly reduced in LADA patients compared with non-diabetic controls but not so obviously as that in patients type 1 diabetes. Pancreatic volume could effectively predict βcell function in LADA patients.

### 0091

### Determination of N$^{\varepsilon}$-(carboxymethyl) lysine (CML) in bones of streptozotocin-induced diabetic rats using LC-MS/MS and its comparison with bone strength

Clinical Diabetes

S. Arakawa[1,2], Y. Maruyama[2], J.-i. Shirakawa[2], M. Saito[1], K. Marumo[1] and R. Nagai[2]
[1]*Jikei University School of Medicine, Tokyo, Japan,* [2]*Tokai University School of Agriculture, Kumamoto, Japan*

Objectives: Pathogenesis of diabetes deteriorates bone strength, and Advanced Glycation End-products (AGEs) is considered as its one possible explanation.

AGEs is a generic term for over 40 compounds. Among them, pentosidine is commonly measured because its fluorescent feature makes the measurement relatively easy and it is reported to correlate with the total sum of fluorescent AGEs. And we have demonstrated in a prospective study that the urinary levels of pentosidine is a marker for the risk of fracture independent of bone mass.

With the advancement in the measurement technique, however, it becomes clear that pentosidine exists in bone in smaller quantities compared with other AGEs, and pentosidine in itself does not necessarily determine bone strength.

In the present study, we measured the levels of N$^{\varepsilon}$-(carboxymethyl) lysine (CML), a major AGE other than pentosidine in normal and diabetic bones using liquid chromatography tandem mass spectrometry (LC-MS/MS) and compared them with bone strengths using 3-point bending test.

Method: Bone samples were harvested from bilateral femoral bones of normal and streptozotocin-induced diabetic rats (DM rats). After pulverization of ipsilateral bones by liquid nitrogen, delipidation and demineralization, bone samples were reduced, hydrolyzed and loaded onto positive cation-exchange column. Then CML was determined by LC-MS/MS. And the maximum loads to produce fractures were measured in contralateral bones using 3-point bending test.

Results: Compared with normal rats, the levels of CML increased in bones of DM rats. And the maximum loads were significantly decreased in bones of DM rats compared with those of DM rats.

Conclusions: Our present study demonstrates that measured AGE can be a causative compound for the deterioration of bone strength just like pentosidine. Researches at the tissue, cellular and molecular levels have not provided a reasonable mechanism that AGEs may have on the deterioration of bone strength. Taken together, the determination of various AGEs using new measurement techniques like LC-MS/MS will be beneficial to understand the mechanism by which bone strength is deteriorated because of diabetes and other disorders.

### 0092

### Modifying a Japanese type 2 diabetes female who values her role as a mother by applying 'nursing practice model focusing on the attitudes at meals'

Education and Care Delivery

Y. Hishino[1], K. Endo[2], T. Nakagami[1,3], Y. Yoshida[1], R. Nojiri[1], Y. Uematsu[1], E. Ushikubo[1], M. Mori[1], M. Oota[1], A. Mizunuma[1], Y. Numata[1], K. Hayashida[1] and O. Tomonaga[1,3]
[1]*Diabetes And Lifestyle Center, Tomonaga Clinic, Tokyo, Japan,* [2]*Yamagata Prefectual University of Health Sciences, Yamagata, Japan,* [3]*Diabetes Center, Tokyo Women's Medical University School of Medicine, Tokyo, Japan*

Background: Traditionally, in Japan mothers play a significant role in the family as a primary care taker and can affect the attitudes and responses towards diabetes. Therefore, the mothers with diabetes have not been able to give priority to their own lives; thus, a reliable and efficient nursing model is necessary to promote the modification of their behaviors and to improve their health.

Objectives: We used 'A nursing model focusing on the attitudes at meals ' by Endo (2012) a patient-centered nursing practice model for a middle-aged type 2 diabetic female, designed to support their gender role and modify self-care actions via a change of their inner world.

Method: Participant was a Japanese type 2 diabetic having two different aspects in the family, role as a mother and the other the fact that she is diabetic. The Diabetes Educator (CDE) who met regularly with the participant, requested her to draw a sketch of a scene from the dining table. The researcher asked the attitudes at dining tables while observing the sketches, and investigated "the sense of her gender roles" expected by the family.

Results: Following this nursing model with the help of the CDE, the patient identified, 1) she was dedicated to putting the needs of her gender role as a mother before her needs as a diabetic. 2) preparing meals and taking care of the family were her gender responsibilities as a mother which she came to confirm and accept. These understandings influenced her self-care action towards her diabetes, and came to learn valuable skills in meal preparation and food choices. Regarding herself as diabetic, she was now able to modify her self-care actions. She asked the CDE how to incorporate carbs, fruits and daily products in her diet without causing any disruption in the family's meal preferences. And helped to keep her position as a symbolic Japanese mother who could manage her diabetes well at the same time.

Conclusion: "A nursing model focusing on the attitudes at meals" suited successfully to a Japanese house wife with type 2 diabetes. This is the result of the transformation of "sense of the gender role" to "meaningful sense of the gender role" from her inner world. It was essential to protect roles of the Japanese mothers within the families, in order to keep their positive thinking and attitude towards diabetes. This study was supported by JSPS KAKENHI Grant Number 5463394

### 0093

### Red wine resveratrol protects against metabolic vascular dysfunction

Microvascular Complications

C. L. H. Carol, L. J. Yun, L. Jian, W. Li, T. X. Yu and H. Yu
*Institute of Vascular Medicine and School of Biomedical Sciences, Chinese University of Hong Kong, Hong Kong*

Objectives: Resveratrol is a phytoestrogen found in more than 70 plants. As red wine resveratrol has been widely publicized to produce health benefits, it is essential to reveal more detailed cellular and molecular mechanisms underlying vascular and metabolic benefits of resveratrol through acting on newly elucidated molecular targets. Endothelial dysfunction is one of the earliest events in the development of diabetic vascular and renal complications. We have recently elucidated the importance of bone morphogenic protein-4 (BMP4) and cyclooxygenase-2 (COX-2) axis in endothelial dysfunction in hypertension and diabetes (Wong et al., 2010; Zhang et al., 2014). The present study aims at investigating whether resveratrol achieve its vaso-protection in obese and diabetic mice through inhibiting BMP4-COX-2 signaling cascade in endothelial cells.

Methods: Endothelial function or vascular reactivity was determined on wire myograph; real-time PCR was used to measure expression of mRNA; an adenovirus was constructed to overexpress COX-2; and reactive oxygen species (ROS) was estimated by DHE fluorescent dye.

Results: Treatment with resveratrol at nanomolar concentrations (10–100 nM) reverses BMP4-induced impairment of endothelium-dependent relaxations in the aortas of C57 mouse aortas. At micromolar concentrations (1–3 μM) inhibits acetylcholine-induced endothelium-dependent contraction in mouse carotid arteries in the presence of nitric oxide synthase inhibitor and this contraction can be abolished by indomethacin, a COX inhibitor. In isolated mouse aortas transduced with COX-2 over-expressing adenovirus for 48 h impairs endothelium-dependent relaxations and this effect is reversed by co-treatment with resveratrol (100 nM). However, resveratrol does not affect BMP4-induced COX-2 upregulation in human endothelial cells. Resveratrol also inhibits BMP4-stimulated ROS over-production in endothelial cells.

Conclusion: We provide novel experimental evidence demonstrating that red wine resveratrol at low concentrations is effective to preserve endothelial function and limits vascular oxidative stress through targeting the BMP4-COX-2 pathway, thus supporting the vascular benefits observed in humans.

© 2016 The Authors. Journal of Diabetes Investigation published by
Asian Association for the Study of Diabetes (AASD) and John Wiley & Sons Australia, Ltd

1. Wong WT et al., (2010) Bone morphogenic protein-4 impairs endothelial function through oxidative stress-dependent cyclooxygenase-2 upregulation: Implications on hypertension. Circulation Research 107(5):984–991.
2. Zhang Y et al., (2014) Inhibition of bone morphogenic protein-4 restores endothelial function in db/db diabetic mice. Arteriosclerosis, Thrombosis and Vascular Biology 34(1):152–159.

### 0094

### Outcomes of radioactive iodine after 5 years of follow-up and pattern of temporal changes in serum free thyroxine levels 48 weeks after radioactive iodine

Thyroid and Osteoporosis

Y. C. Au Yeung, C. M. Ng, Y. W. Ng, C. H. Choi and S. C. Tiu
*Department Of Medicine, Queen Elizabeth Hospital, Hong Kong*

Objective: It is known that serum thyroid hormone levels can rise after radioactive iodine therapy (RAI) for hyperthyroidism as a result of transient radiation thyroiditis, before it declines as a consequence of radiation-induced destruction of thyroid cells.

Reports showed that around 80–90% of patients developed hypothyroidism after RAI and 10–20% required second dose of treatment.

This study aims to study the temporal course of change in serum free T4 (fT4) level 48 weeks after RAI and successful rate after 5 years of follow-up.

Methods: 56 hyperthyroid patients who received RAI for the first time between 1st July 2009 to 31st March 2010 were arranged to have fT4 measured at week 0, 1, 2, 3, 4, 5, 10, 14, 18, 24, 36 and 48 after RAI.

A successful outcome was defined as achievement of hypo- or eu-thyroidism at 1 year and 5 year after RAI.

The relationship of baseline clinical parameters and outcome was studied by Speaman's rank-order correlation. Multivariate analysis was performed using logistic regression. The degree of fluctuation in fT4 within different time periods was assessed by analysis of variance (ANOVA).

Results: 69.2% ($n$ = 40) and 83.9% ($n$ = 47) of patients had a successful outcome at 1 year and 5 year respectively. Out of the 47 successfully treated patients 38.3% of them ($n$ = 18) remained euthyroidism at 5 year.

Only shorter duration of anti-thyroid drug treatment before RAI was associated with successful outcome at 1 year and 5 year.

For the change in fT4, the mean fT4 dropped from baseline (25.06 + /- 11.4pmol/L) to a nadir at around week 4 by 23% (19.24 + /- 9.06 pmol/L), then rose to a peak at around week 10 (Figure).

The degree of fluctuation in fT4 within a time interval, as reflected by the coefficient of variation (CV), was shown in Table. The fluctuation was greatest between week 6 to week 12.

Conclusion: RAI led to remission in 4/5 of hyperthyroid patients. Shorter duration of prior medication was associated with good outcome.

Wide fluctuations in fT4 levels occurred after RAI, and close monitoring, especially between 6 and 12 weeks, is warranted.

### 0095

### Significant importance in the relationship between pharmacists and patients in pharmacies that cover mainly diabetes patients

Education and Care Delivery

A. Sekine[1], H. Fujii[2], C. Takayama[1], T. Mori[1], T. Ota[1], Y. Asada[1], M. Nogawa[3], Y. kawagoe[2], Y. Watanabe[2], T. Miyakawa[3] and A. Nonaka[1]
*[1]Yamato Prescription Center, Higashiyamato-city, Japan, [2]Tama-center Mirai Clinic, Tama-city, Japan, [3]Kunitachi Mirai Clinic, Kunitachi-city, Japan*

We have four pharmacies that are receiving prescriptions from hospitals and clinics in the west Tokyo. The Pharmacies are A and B located next to a diabetes clinic, C located close to a pediatric and an otorhinolaryngology clinic, and D covered more than 160 (relatively many in Japan) medical facilities. We investigated patients' satisfaction especially for the pharmacies that cover regular patients with chronic diseases by comparing one another.

We administered the questionnaire to 1155 patients (male 45%, ~50y/o 31%, 50s 16%, 60s 21%, 70s 22%, 80y/o~ 10%) in the four pharmacies. We asked them degree of satisfaction using the Likert scale about the service of the pharmacy; Q1) waiting time, Q2) atmosphere and comfort, Q3) opening hours, Q4) attitude of pharmacists, Q5) attitude of receptionists, Q6) explanation of medicines, Q7) generic substitution, Q8) consideration for their privacy, Q9) reliance, Q10) hygiene, Q11) variation of medical supplies including Over-The-Counter drug, Q12) variation of health foods, and also their general evaluation (scale out of 5). We analyzed the relationship between the general score and each score of questions by multiple regression analysis (MRA). They also answered whether they have a primary pharmacy, the frequency of use, and the reasons why they chose that pharmacy.

Patient population is the youngest in C, the eldest in D (ANOVA; $P < 0.01$). Half of the patients in A and B are in their 50s and 60s. General score was highest in D and lowest in C ($P < 0.05$). The scores of Q1) to Q3) and Q8) to Q12) were different among generations ($P < 0.01$). The scores of Q2) to Q6) and Q8) to Q12) were different among pharmacies ($P < 0.05$). As a result of the MRA of each pharmacy, Q4) and Q10) have significant positive correlations in A and B. Only in A, Q12) was chosen as a significant factor. In the MRA of all pharmacies ($R^2 = 0.743$), ten variables have significant positive correlations with general score, ranked as influence in the following order: Q9), Q10), Q2), Q1), Q4), Q6), Q5), Q3), Q8), and Q7). One of the reasons why they chose A and B was; the efficient and close relationship with the clinics.

In conclusion, important factors affecting general evaluation were different according to pharmacies. The results suggest that the relationship between pharmacists and patients have an impact upon patients' satisfaction in pharmacies especially serving diabetes patients. Enriching health foods variation (and their information) can be a better service for health conscious people such as diabetes patients.

### 0096

### Effects of royal jelly supplementation on hyperglycemia and body weight in KK-Ay mice

Obesity

R. Watadani, D. Sasaki, J. Koto and K. Matsumoto
*Kyoto Sangyo University, Kyoto, Japan*

Royal jelly (RJ) has several pharmacological activities, including antitumor activity, anti-inflammatory activity, antioxidative activity and antiaging activity. Moreover, it has been reported that RJ increases insulin sensitivity and decrease blood glucose level in nondiabetic human. However, effects of RJ are not fully understood under condition of diabetes or hyperglycemia. Therefore we investigated whether RJ exerts protective effect on hyperglycemia or body weight using KK-Ay mice which are obese-type 2 diabetes mice.

We used female 8-week-old KK-Ay mice. Mice were divided into control and treatment groups. The treatment group was given RJ by gavage at 10 mg/kg or 100 mg/kg for 3 weeks. The oral glucose tolerance test (OGTT) was performed on day 7 and 21 after 24 h fasting. After 1 week after the end of OGTT, mice were killed and dissected liver and intrabdominal fat, muscle tissue. These tissues were extracted for mRNA for Isogen-II.

To examine effects of RJ in diabetic or hyperglycemic situation, we administered RJ orally to female KK-Ay mice at 8-week-old for 3 weeks. We showed that both the body weight and the blood glucose levels of the RJ treated mice were significantly lower than those of PB treated mice. However, there was no difference in the plasma insulin levels. Then, we examined gene expression levels involving obesity and blood glucose in liver and fat tissues from KK-Ay mice using real-time PCR. The expression of Ppargl in RJ treated mice was significantly increase in the livers than those of PB treated mice, and Pparg2 was significantly increased in the abdominal fats. The reduction of Ppargl expression in liver may suppress fat accumulation and gluconeogenesis. Moreover, the increase of Pparg2 expression may normalize lipid metabolism and glucose homeostasis.

In summary, these date suggest that RJ has may pharmacological effects through the reduction of both body weight and high plasma glucose under diabetogenic condition. Our data also suggest that the reductions of both body

© 2016 The Authors. Journal of Diabetes Investigation published by Asian Association for the Study of Diabetes (AASD) and John Wiley & Sons Australia, Ltd weight and blood glucose levels in RJ treated mice may occur through a direct action of RJ to Pparg expression.

### 0097

**Outpatient nursing care for the middle-aged woman of Type 2 diabetes 'Development of the nursing practice model focused on the attitudes at meals'**

Education and Care Delivery

K. Endo[1] and Y. Hishino[2]

[1]*Yamagata Prefectual University of Health Science, Yamagata, Japan,* [2]*Tomonaga Clinic, Tokyo, Japan*

BACKGROUND: It is difficult for the middle-aged woman to think of their own self-care, while going through menopause, and also meeting life events such as deprivation and changes, which causes deterioration to self-conceit feelings.

OBJECTIVES: The purpose of this research is to develop a nursing practice model which focused on the attitudes at meals, in order to support type 2 middle- aged female.

METHOD: The participants were in the range of 45–60 years old. The diabetes Educator having a regular visit with the participants, asked them to draw a sketch of a typical family meal, for helping them to describe the inner world. Qualitative synthesis method (KJ method) was used to analyze the participants' narration.

RESULTS: Hypothetical model was created based on two female participants in their 50 s. From the participants' narration, few factors of the inner world rose to the surface, which created the females attitudes at meals. The factors were a sense of gender role, a sense of self-regulation and also understanding health conditions and the supporting resources. As they narrate, the factors caused changes, where the Type 2 Diabetic middle-ages female participants started to think of their own self-care actions. As a conclusion, it led them to find out a way to live creatively, in character. We applied this hypothetical model to 10 of the participant whose age were 45–60 years old, 2–13 times per person during their visit, and confirmed this model validated.

CONCLUSIONS: This nursing model promotes and modifies female Diabetic patients from the inner world on their self-care actions, by focusing on their role of attitudes at meals. For this reason, this nursing model was confirmed validated, by preparing the readiness towards behavior modification, and made the Diabetes education effective.

### 0098

**Vitamin D deficiency increases the risk of peripheral neuropathy in chinese patients with type 2 diabetes**

Clinical Diabetes

Rui He, Yanyun Hu, Fang Liu, Hui Zeng, Lianxi Li and Weiping Jia

*Shanghai Jiaotong University Affiliated Sixth People's Hospital, Shanghai Clinical Medical Centre of Diabetes, Shanghai Key Laboratory of Diabetes, Shanghai Key Clinical Centre of Metabolic Diseases, shanghai, China*

OBJECTIVE: To investigate the relationship between vitamin D deficiency (VDD) and diabetic peripheral neuropathy (DPN)

METHODS: A total of 861 patients with type 2 diabetes were recruited in this cross-sectional study. VDD was defined as serum circulating 25-hydroxyvitamin D (25(OH)D) level <20 ng/mL. DPN was evaluated by neurological symptoms, neurological signs, neurothesiometer, and electromyogram.

RESULTS: The patients with DPN had significantly lower serum 25(OH)D concentration (15.59 ± 7.68 ng/mL) and higher prevalence of VDD (80%) than patients with signs of DPN (17.66 ± 7.50 ng/mL; 64.5%) and non-DPN patients (18.35 ± 6.60; 61.7%) (all $p < 0.01$). The prevalence of DPN and signs of DPN in patients with VDD was also higher than those without VDD (both $p < 0.01$). After adjusting for all potential confounders, VDD was still associated with increased risk of DPN [odds ratio (OR) 2.59 (1.48–4.53)] ($p < 0.01$). Logistical regression analysis further revealed that VDD was an independent risk factor for DPN ($\beta = 0.88$) ($p < 0.01$). ROC analysis indicated that serum 25(OH)D < 17.22 ng/mL hinted the signs of DPN and serum 25(OH)D < 16.01 ng/mL predicted the occurrence of DPN (both $p < 0.01$).

CONCLUSIONS: VDD is an independent risk factor for DPN and may be a potential biomarker for DPN in Chinese patients with Type 2 diabetes.

### 0099

**Association between angiopoietin-like protein 4 and arterial stiffness in patients with type 2 diabetes**

Macrovascular Complications

H.-K. Park, M.-K. Kwak, H.-J. Kim, D.-W. Byun, K.-I. Suh and M.-H. Yoo

*Soonchunhyang University of Hospital, Seoul, Republic of Korea*

Angiopoietin-like protein (ANGPTL) 4 has been shown to modulate lipoprotein metabolism mainly by inhibiting lipoprotein lipase activity. Recent findings in animal studies suggest that ANGPTL4 plays a role in the pathophysiology of dyslipidemia and atherosclerosis. However, the impact of ANGPTL4 on cardiovascular risk in type 2 diabetes mellitus (T2DM) remains unclear. Therefore, we investigated the relationship between plasma ANGPTL4 levels and subclinical atherosclerosis in patients with T2DM.

We recruited a total of 108 Korean patients with T2DM. Subjects with known cardiovascular diseases, chronic kidney disease, or active infection were excluded. Plasma ANGPTL4 levels were determined. We assessed vascular health status by measuring doppler-derived aortic pulse-wave velocity (PWV) and carotid intima-media thickness (IMT). Plasma ANGPTL4 correlated positively with aortic PWV, apolipoprotein B, and C-reactive protein, and negatively with high-density lipoprotein (HDL) cholesterol. However, ANGPTL4 did not correlate with carotid IMT, or triglyceride. Aortic PWV correlated with age ($r = 0.37$, $P < 0.01$), body mass index (BMI; $r = -0.21$, $P < 0.05$), and ANGPTL4 ($r = 0.23$, $P < 0.05$). A multiple regression analysis showed that plasma ANGPTL4 was independently associated with aortic PWV.

In summary, these results demonstrate that ANGPTL4 is associated with arterial stiffness in patients with T2DM, providing more insight into the role of ANGPTL4 in the pathogenesis of atherosclerosis in T2DM.

### 0100

**Improved automated glucose clamps using algorithm variations with the clampart device**

Clinical Diabetes

C. Benesch, L. Bochmann, J. Haensler and T. Heise

*Profil Germany, Neuss, Germany*

OBJECTIVES: The pharmacodynamic effects of anti-diabetic drugs are usually characterized in glucose clamp experiments where variable glucose infusion rates (GIR) keep blood glucose concentrations (BG) as closely as possible to a predefined target level. For a high glucose clamp quality low BG- und GIR-variability are desirable. In automated clamps GIR are calculated by implemented algorithms such as the Biostator algorithm published in 1982. For ClampArt, a new, CE-marked state-of-the-art glucose clamp device, we improved this algorithm to reduce the oscillations of both BG and GIR.

METHODS: We first improved the algorithm by numerical simulations of glucose clamps (in-silico). With the results of the simulations we started in-vitro experiments using a ClampArt device and a container with water and glucose as 'test subject'. Finally, we performed a small number of in-vivo clamps with real subjects using ClampArt with the optimized algorithm.

RESULTS: All three approaches showed that the improved algorithm reduced the amplitude of oscillation of both BG and GIR. The in-silico optimization of the algorithm showed a reduction in the amplitudes of the oscillations in BG to 43% and GIR to 66% of those observed with the Biostator algorithm. The in-vitro experiments showed similar results with a reduction of oscillations of both BG (61%) and GIR (76%). Finally, we observed in the in-vivo experiments reduced amplitudes of oscillations for BG (35%) and for GIR (65%).

CONCLUSIONS: In-silico, in-vitro and in-vivo experiments showed that an improved algorithm reduced BG-oscillations by 35%–61% and GIR oscillations

© 2016 The Authors. Journal of Diabetes Investigation published by Asian Association for the Study of Diabetes (AASD) and John Wiley & Sons Australia, Ltd by 65%–76%. This optimized algorithm will now be implemented in ClampArt and used for future glucose clamp studies.

### 0101
### Possible proteolyzation of circulating betatrophin in human serum

Other Endocrine Topics

S. Tokumoto, Y. Hamamoto, E. Okamura, Y. Kawasaki, E. Yamaguchi, S. Honjo and A. Hamasaki
*Tazuke Kofukai Foundation, Medical Research Institute, Kitano Hospital, Osaka, Japan*

OBJECTIVES: Betatrophin (BTH) is a secreted peptide which stimulates beta cell proliferation in mouse and rat models. However, the results of previous clinical investigations on BTH levels in human serum are inconsistent. We hypothesized that circulating BTH could be proteolyzed in human serum and investigated whether circulating BTH levels change when blood samples are collected with or without protease inhibitors.

METHODS: We obtained 3 sets of fasting blood samples collected into both a serum tube and a P800 tube (Becton Dickinson, NJ, USA) which contained a protease inhibitor cocktail from each healthy subject (n=10) and incubated each set at room temperature for different time intervals (0, 3 hours and 6 h), and afterwards frozen below −20°C until the assay. We also withdrew fasting blood samples into the same set of tubes from T2DM patients ($n$ = 20). Circulating BTH levels were determined using two commercial ELISA kits: N-terminal kits based on antibodies against the N-terminus of BTH which recognize (Wuhan Eiaab Science, Wuhan, China, Catalogue No.E11644 h), and C-terminal kits based on antibodies against the C-terminus of BTH (Phoenix Pharmaceuticals, CA, USA, Catalogue No.EK-051-55).

RESULTS: Both N-terminal and C-terminal ELISA kits showed linear regression ($r$ = 0.99) when measuring a human recombinant betatrophin. BTH levels in healthy subjects decreased in a time-dependent manner when assayed by C-terminal kits, but remained constant over time when assayed by N-terminal kits. Both ELISA kits demonstrated significant differences between BTH levels in serum tubes and in P800 tubes (P < 0.05, Wilcoxon test). The BTH levels in P800 tubes were much higher than those in serum tubes (31.91 ± 12.38 ng/mL vs 1.89 ± 1.70 ng/mL, by C-terminal ELISA kits; 599.44 ± 510.46 pg/mL vs 181.01 ± 45.48 pg/mL, by N-terminal ELISA kits). A significant correlation between BTH levels in serum tubes and in P800 tubes was found when measured by N-terminal kits (Spearman's rank-order correlation; $r$ = 0.70, P < 0.001), but not when measured by C-terminal kits ($r$ = 0.21, $P$ = 0.35).

CONCLUSION: Our results provide new evidences on the proteolytic regulation of BTH, suggesting the existence of a protease for BTH in human serum. The discrepancy between serum and protease inhibited plasma levels of circulating BTH must be taken into consideration in interpreting results of these commercial ELISA kits. Further studies are needed to elucidate the proteolytic regulation of BTH.

### 0102
### Two candidate genes for the onset of type 2 diabetes associated with obesity using new animal models of rats

Genetics and Epigenetics

D. Sasaki, J. Koto, R. Watadani and K. Matsumoto
*Kyoto Sangyo University, Kyoto, Japan*

Type 2 diabetes is brought on by sedentary life style, rich nutrition and obesity. However, little is known how obesity induces the onset of type 2 diabetes. To elucidate these mechanisms, it is necessary to identify diabetogenic gene associated with obesity. In this study, we investigate to identify diabetogenic gens associated with obesity using new obese animal models.

We generated several congenic strains: (1) single lean congenic introgressed Nidd2 QTL derived from OLETF rat into F344 (F.O-*Nidd2/of*), (2) single obese congenic introgressed *Lepr*$^{-/-}$ locus into F344 (F.ZF-*Lepr*) and (3) double congenic rats having obese and *Nidd2* QTL region (F.ZF-*Lepr*&*Nidd2/of*). F344 starain was purchased for normal lean control. Each strain was phenotyped for plasma glucose by oral glucose tolerance test (OGTT), insulin levels and insulin resistance by insulin tolerance test (ITT). In 25 weeks of age, they were sacrificed and then liver, skeletal muscle and abdominal fat tissues were dessected. RNA and protein levels were determined using reverse transcription-quantitative PCR and western blotting analyses, respectively.

The single lean congenic strain, F.O-*Nidd2/of* showed no significant changes in glucose levels compared with normal lean control F344 strain. However, the double congenic strain, F.ZF-*Lepr*&*Nidd2/of* (*Lepr*$^{-/-}$ and *Nidd2/of*) showed significantly higher glucose levels (60, 90, 120 min after glucose loading: $P$ < 0.001 vs F.ZF-*Lepr*), and significantly lower hypoglycemic response to insulin than those of the obese control strain, F.ZF-*Lepr* (*Lepr*$^{-/-}$) (30, 60, 90, 120 min after insulin injection: $P$ < 0.05 vs F.ZF-*Lepr*). These phenotypes were observed specifically in the obese strains, but not in the lean strains. These results indicate that the *Nidd2/of* locus harbours a diabetogenic gene associated with obesity. We measured the expression of 60 genes in the *Nidd2/of* QTL region between the strains and found that the levels of the mRNA expression of five genes were significantly different between the strains under condition of obesity. Three genes among the five genes were differentially expressed both in obese and lean rats, while the other two genes, *Coq2* and *Plac8* were differentially expressed specifically only in the obese rats, suggesting that these genes are candidates for the onset of type 2 diabetes associated with obesity in rats (*Coq2*: $P$ = 0.00387 and *Plac8*: $P$ = 0.00122)

Our data show that *Coq2* and Plac8 were candidate genes for the onset of type 2 diabetes associated with obesity in rats.

### 0107
### Oxygen uptake at anaerobic threshold in obese patients with Type2 diabetes mellitus

Exercise

R. Matsuki[1], Y. Hirasawa[1], Y. Umemoto[1], T. Ebisu[1], D. Yabe[2,3], T. Kurose[2,3] and Y. Seino[2,3]
[1]*Department of Rehabilitation, Kansai Electric Power Hospital, Osaka, Japan,* [2]*Center for Diabetes, Endocrinology and Metabolism, Kansai Electric Power Hospital, Osaka, Japan,* [3]*Yutaka Seino Distinguished Center for Diabetes Research, Kansai Electric Power Medical Research Institute, Osaka, Japan*

INTRODUCTION: Indicators of aerobic fitness such as anaerobic threshold (AT) are often used for functional evaluation of various populations. It was reported that aerobic capacity is associated with cardiac output, lung capacity, body composition, and carbohydrate and lipid oxidation capacity. In patients with Type2 Diabetes Mellitus (T2DM), oxygen uptake ($VO_2$) at AT is decreased in comparison with that of a healthy population. The relationship between $VO_2$ at AT ($ATVO_2$) and glycemic control, body composition, and lipid oxidation capacity in obese patients with T2DM has not been established.

PURPOSE: The purpose of this study was to examine the relationship $VO_2$ at AT with glycemic control, body composition, and lipid oxidation capacity in obese patients with T2DM.

METHOD: Nineteen T2DM patients (mean age: 43.1 ± 5.5 years, mean BMI: 29.8 ± 3.8 kg/m$^2$) underwent expiration gas analysis while performing incremental exercise test on a cycle ergometer. Subjects were excluded whose BMI was less than 25. The Ramp protocol (20 W/min) was used. $VO_2$, carbon dioxide output (VCO2), and maximum lipid oxidation were measured during the exercise test. The amount of lipid oxidation (g/min) during exercise was calculated by Bruzstein formula. AT was evaluated as ventilation threshold by V-slope method. $ATVO_2$ was compared to the standard value of the Japanese Circulation Association. $ATVO_2$ clarified the correlation between body mass index (BMI), glycosylated hemoglobin, fasting plasma glucose, skeletal muscle index (SMI), visceral fat sectional area, and maximum amount of lipid oxidation. SMI was calculated as skeletal muscle mass divided by body weight.

RESULT: $ATVO_2$ in obese patients with T2DM averaged 1182.2 ± 189.4 mL, which was 89.4 ± 21.7% compared to the standard value. $ATVO_2$ showed a significant negative correlation with the visceral fat sectional area ($r$ = –0.52, $P$ < 0.01) and a significant positive correlation with the maximum lipid oxidation amount, SMI (each $r$ = 0.74. $P$ < 0.01, $r$ = 0.76. $P$ < 0.01).

DISCUSSION: Aerobic capacity for obese patients with T2DM was generally decreased, and was influenced by skeletal muscle mass and visceral fat and lipid oxidation capacity during exercise. Aerobic capacity of T2DM was evaluated as in obese patients, in consideration of the relationship between visceral fat, lipid oxidation capacity and insulin resistance.

© 2016 The Authors. Journal of Diabetes Investigation published by Asian Association for the Study of Diabetes (AASD) and John Wiley & Sons Australia, Ltd

### 0109

### Efficacy and safety comparison of sitagliptin and glimepiride in elderly Japanese patients with type 2 diabetes: START-J

Therapeutics of Diabetes

Y. Terauchi[1,2], E. Araki[1,3], H. Ishida[1,4], M. Kitaoka[1,5], J. Satoh[1,6], D. Yabe[1,7], Y. Yamada[1,8], N. Shihara[1] and Y. Seino[1,9]

*[1]Japan Association for Diabetes Education and Care, Tokyo, Japan, [2]Department of Endocrinology and Metabolism, Yokohama City University Graduate School of Medicine, Kanagawa, Japan, [3]Department of Metabolic Medicine, Faculty of Life Sciences, Kumamoto University, Kumamoto, Japan, [4]Third Department of Internal Medicine, Division of Diabetes, Endocrinology and Metabolism, Kyorin University School of Medicine, Tokyo, Japan, [5]Division of Endocrinology and Metabolism, Showa General Hospital, Tokyo, Japan, [6]NTT EAST Tohoku Hospital, Miyagi, Japan, [7]Center for Diabetes, Endocrinology and Metabolism, Kansai Electric Power Hospital, Osaka, Japan, [8]Department of Endocrinology, Diabetes and Geriatric Medicine, Akita University School of Medicine, Akita, Japan, [9]Kansai Electric Power Hospital, Osaka, Japan*

Objective: DPP-IV inhibitors which rarely cause hypoglycemia are possible to be anti-diabetic agent as the first line therapy for elderly patients with type 2 diabetes (T2DM) who could not manage hypoglycemia sufficiently. The purpose of this study was to compare the efficacy and the safety of sitagliptin and glimepiride in drug naïve elderly Japanese patients with T2DM.

Subjects: Patients with T2DM who were OHA naïve or on α-GI/biguanide monotherapy (to be washed out 4 weeks prior to randomization). Age ≧ 60 y.o., HbA1c ≦ 6.9%, >8.9%.

Methods: A randomized, controlled, open-labeled, multicenter study was performed for 52 weeks. Starting dose for sitagliptin was 50 mg per day, could be increased up to 100 mg (25–50 mg; 30 ≦ GFR < 50). Starting dose for glimepiride was 0.5 mg per day, could be increased up to 6.0 mg. After that, each investigator titrated dose to aim at FPG < 130 mg/dL, with concerning about hypoglycemia.

Results: Analyzed subjects were 291 patients (148/143:sitagliptin / glimepiride) whose written informed consent were obtained and who started administration of the study drugs. Average age was 70.3 ± 5.6 / 71.1 ± 5.6 (yr), BMI was 24.1 ± 3.3 / 24.5 ± 3.9 and HbA1c was 7.5 ± 0.7 / 7.5 ± 0.7 (%), respectively. Any parameter did not have significant difference between the randomized two groups. HbA1c decreased significantly within 24 weeks in both groups. The changes in HbA1c from baseline to 24 weeks were –0.69 ± 0.62% (sitagliptin) and –0.86 ± 0.65% (glimepiride), respectively. While glimepiride group had significantly larger decrease in HbA1c ($P$ = 0.008) at 24 weeks, there was no significant difference between the two groups at 52 weeks (–0.65 ± 0.59% / –0.78 ± 0.69% $P$ = 0.069). A significant difference was observed in the changes in body weight from baseline to 52 weeks between the two groups (–0.37 ± 2.44 kg / 0.31 ± 2.92 kg $P$ = 0.043). The incidences of hypoglycemia were 7 (sitagliptin) and 23 (glimepiride) patients, respectively. Glimepiride group had significantly more frequent occurrence of hypoglycemia ($P$ = 0.002).

Conclusion: Our results showed that sitagliptin had slightly lower efficiency in 24 weeks, but had no significant difference in 52 weeks and higher safety about hypoglycemia. Sitagliptin might be one of the best choices for elderly patients with T2DM for whom hypoglycemia and its related effects are of special concern.

### 0110

### Large scale investigation of DPP-IV inhibitors therapy in Japan: UNITE study

Therapeutics of Diabetes

Y. Yamada[1,2], E. Araki[1,3], H. Ishida[1,4], M. Kitaoka[1,5], J. Satoh[1,6], Y. Terauchi[1,7], D. Yabe[1,8], N. Shihara[1] and Y. Seino[1,9]

*[1]Japan Association for Diabetes Education and Care, Tokyo, Japan, [2]Department of Endocrinology, Diabetes and Geriatric Medicine, Akita University School of Medicine, Akita, Japan, [3]Department of Metabolic Medicine, Faculty of Life Sciences, Kumamoto University, Kumamoto, Japan, [4]Third Department of Internal Medicine, Division of Diabetes, Endocrinology and Metabolism, Kyorin University School of Medicine, Tokyo, Japan, [5]Division of Endocrinology and Metabolism, Showa General Hospital, Tokyo, Japan, [6]NTT EAST Tohoku Hospital, Miyagi, Japan, [7]Department of Endocrinology and Metabolism, Yokohama City University Graduate School of Medicine, Kanagawa, Japan, [8]Center for Diabetes, Endocrinology and Metabolism, Kansai Electric Power Hospital, Osaka, Japan, [9]Kansai Electric Power Hospital, Osaka, Japan*

Objective: DPP-IV inhibitors were released as new hypoglycemic agents and have been widely used, but information, such as long-term efficacy, side effects, and more effective prescription, is still insufficient. The purpose of this study was to investigate the efficacy and the safety of DPP-IV inhibitors in daily clinical practice.

Methods: Patients with type 2 diabetes mellitus newly prescribed with DPP-IV inhibitors were enrolled. We evaluated the patient characteristic and the clinical data of 3 months, 6 months, 1 year and 2 years after the enrollment.

Results: 11,794 patients were enrolled. We analyzed data from 8,769 patients whose baseline data were obtained and who started administration of DPP-IV inhibitors. Mean age was 63.4 ± 11.7 years old, BMI was 24.9 ± 4.3 and HbA1c was 7.9 ± 1.3 (%). 22.9% of the patients were drug naive. The others had received other oral hypoglycemic agents, in which 33.4% were replaced with DPP-IV inhibitors and 43.6% were added-on with DPP-IV inhibitors. At 3 months, mean HbA1c significantly decreased to 7.2 ± 1.0% and remained unchanged until 2 years. Mean body weight had not changed. To evaluate the maintenance of efficacy, based on the changes in HbA1c from 3 months to 2 years, we divided the patients into three groups – improvement group (< 0%), no change group (0 ~ 0.35%), and deterioration group (>0.35%). The numbers of patients of each group were 2,129,985, and 1,350, respectively. HbA1c in the deterioration group returned to baseline in 2 years. Body weight significantly decreased in the improvement group (64.4 ± 13.7→63.6 ± 13.7 kg) but increased in the deterioration group (65.5 ± 14.1→65.9 ± 14.1 kg) from baseline to 2 years. Adverse events were reported 1,074 cases and 136 of them were hypoglycemia. While the most of cases of hypoglycemia used therapy combined with SU, 15 cases were on DPP-IV inhibitors monotherapy. Although causal relationship is unknown, 7 acute pancreatitis and 5 pancreatic cancer were reported.

Conclusions: DPP-IV inhibitors had efficacy in an early stage. It was important to control body weight using dietary and exercise therapy to maintain the efficacy. For safety, although severe side effects were rarely reported, we need to be careful for hypoglycemia.

### 0111

### Echo intensity is associated with insulin resistance in patients with type 2 diabetes

Exercise

Y. Hirasawa, R. Matsuki, H. Yamamoto, M. Kusaba, A. Hotta and Y. Umemoto

*Department of Rehabilitation, Kansai Electric Power Hospital, Osaka, Japan*

Objectives: Echo intensity (EI) on ultrasonography images of skeletal muscle is believed to reflect muscle quality. Previous studies have shown that EI is associated with muscle strength, muscle size and cardiovascular performance. The aim of the present study was to investigate whether muscle quality based on EI is associated with insulin resistance in patients with type 2 diabetes.

Method: Twenty-six patients with type 2 diabetes (53.3 ± 10.6) participated in this study. Patients receiving insulin therapy or antidiabetic drugs and those with fasting blood glucose levels above 140 mg/dL were

© 2016 The Authors. Journal of Diabetes Investigation published by Asian Association for the Study of Diabetes (AASD) and John Wiley & Sons Australia, Ltd excluded. EI and muscle thickness (MT) were determined by assessing ultrasound images. The value of EI was determined by gray scale analysis. The region of interest included the rectus femoris and vastus intermedius muscle. MT was defined as the sum of the rectus femoris and vastus intermedius muscle. The maximum isometric strength of knee extension at a knee angle of 90° was measured by hand-held dynamometer. Knee extension strength (KES) was calculated as the strength divided by the body weight (BW) or MT. Insulin resistance was evaluated by homeostasis model assessment – insulin resistance (HOMA-IR). All values are shown as the mean ± standard deviation. Correlations between EI, MT, KES, HOMA-IR and clinical characteristics were calculated using Pearson's correlation coefficients. Statistical significance was defined as $P < 0.05$.

Results: HOMA-IR showed a significant positive correlation with EI ($r = 0.41$, $P < 0.05$). HOMA-IR was not significantly associated with MT ($r = -0.32$, $P = 0.11$), KES divided by BW ($r = -0.34$, $P = 0.08$) and KES divided by MT ($r = 0.20$, $P = 0.33$). EI was also significantly associated with MT ($r = -0.39$, $P < 0.05$), but EI was not significantly associated with KES divided by BW ($r = -0.33$, $P = 0.10$), KES divided by MT ($r = -0.17$, $P = 0.40$), age ($r = 0.39$, $P = 0.06$), BMI ($r = 0.12$, $P = 0.58$), HbA1c ($r = 0.11$, $P = 0.96$), and duration of diabetes ($r = 0.28$, $P = 0.18$).

Conclusions: We found a significant positive correlation between EI and insulin resistance. The present results suggest that the EI may be a more important predictor of insulin resistance than muscle strength and muscle size in patients with type 2 diabetes.

### 0112

### Inhibitory effect of esculeoside A, a tomato-derived compound, on the pathogenesis of atherosclerosis, and the changes of its contents during maturation and processing of tomatoes

Macrovascular Complications

C. Furusawa[1], R. Owatari[2], C. Wakabayashi[2], Y. Fujiwara[3] and R. Nagai[1]

[1]*Department of Bioscience, School of Agriculture, Tokai University, Minamiaso, Kumamoto, Japan,* [2]*Department of Food and Nutrition, Laboratory of Biochemistry and Nutritional Science, Japan Women's University, Mejiro, Tokyo, Japan,* [3]*Department of Cell Pathology, Faculty of Life Sciences, Kumamoto University, Kumamoto, Kumamoto, Japan*

Objective: Recently, lifestyle-related diseases such as diabetes, hypertension and dyslipidemia are increasing rapidly. Lifestyle relates diseases cause atherosclerosis and eventually angina pectoris, myocardial infaction and cerebral infarction. We previously discovered that esculesogenin A (EsgA), an aglycone of esculeoside A (EscA) purified from maturated tomato fruits significantly inhibited the accumulation of cholesterol ester (CE) induced by acetylated low density lipoprotein (acetyl-LDL) in human monocyte-derived macrophages (HMDM). Furthermore, esgA prevented the expression of acyl-CoA: cholesterol acyl-transferase (ACAT)-1 protein. Oral administration of esculeoside A to apoE deficient mice significantly reduced the levels of serum cholesterol, triglycerides, LDL-cholesterol, and the areas of atherosclerotic lesions without any detectable side effects. However, the EscA was not detected in processed tomato products. In the present study, we measured EscA contents after processing.

Method: The amounts of EscA was determined by an RI-201H RI detector, (Shodex, Japan) with a Capcellpak C18 reverse phase column (4.6 × 250 mm, Shiseido, Japan) at a flow rate of 1 mL/min at 40°C using a Shimadzu HPLC system. Cherry tomatoes were homogenized in water, filtered, then heated at 50–100°C for 30 min or at 121°C for 20 min, followed by determination of the EscA content by HPLC. Furthermore, EscA contents were measured after tomato was dried by drying oven under different temperature.

Result: The contents of EscA in Cherry tomatoes and Momotaro tomatoes were 21-fold and 9-fold, respectively, higher than that of lycopene, which is the most well-known compound in tomatoes. However, the EscA in crude tomato extract decreased in a temperature-dependent manner. The degradation due to the heating process was inhibited under a pH of 9. Furthermore, the degradation of EscA was prevented in dried tomatoes.

Conclusions: The EscA content differs in the various types of tomatoes and during the heating process used for preservation. This information will be beneficial for developing new functional foods and preventive medicines.

### 0113

### Histone deacetylase regulates insulin signaling via two pathways in pancreatic β-cells

Islet Biology

Y. Sugiura[1], S.-I. Asahara[2], Y. Kawada[1], Y. Ihara[1], M. Hara[1], A. Kanno[2], M. Kimura-Koyanagi[2], T. Matsuda[2], S. Seino[3], W. Ogawa[2] and Y. Kido[1,2]

[1]*Division of Medical Chemistory, Kobe University Graduate School of Health Sciences, Kobe, Hyogo, Japan,* [2]*Division of Diabetes and Endocrinology, Kobe University Graduate School of Medicine, Kobe, Hyogo, Japan,* [3]*Division of Molecular and Metabolic Medicine, Kobe University Graduate School of Medicine, Kobe, Hyogo, Japan*

Objectives: We have previously reported that insulin signaling plays important roles in the regulation of pancreatic β-cell mass, the reduction of which is known to be involved in the development of diabetes. However, the mechanism of insulin signaling alteration in pancreatic β-cells remains unclear. So, we have established two clones of MIN-6 cells with different growth rate by isolating dispersed MIN-6 cells. Using two clonal MIN-6 cells, we confirmed that both expression of IRS2 and insulin signaling in the high proliferation clone were increased. Furthermore, we found that increased IRS2 expression was affected by histone acetylation in IRS2 promoter. Histone acetyl transferase and Histone deacetylase (HDAC) are known as the enzymes that control histone acetylation, but the role of HDACs in pancreatic β-cells remains to be determined. This study was designed to elucidate the role of HDACs in pancreatic β-cells.

Method: We examined the insulin signaling by immunoblot analysis using MIN-6 mouse insulinoma cell lines treated with various HDAC inhibitors. In addition, we examined the expression of HDAC in mouse pancreatic sections.

Results: In order to explore the epigenetic mechanisms involved in the increase in IRS2 expression observed in the high proliferated MIN-6 clone, we examined the expression of IRS2 in MIN-6 cells treated with DNA methyltransferase inhibitor or HDAC inhibitors. Treatment with the DNA methyltransferase inhibitor had no significant effect on the IRS2 expression, while the increased IRS2 expression was observed in MIN-6 cells treated with trichostatin A (TSA), a HDAC class I,II selective inhibitor. However, the insulin signaling in MIN-6 cells treated with TSA was decreased unexpectedly. Next, treatment with Apicidin, a HDAC class I selective inhibitor, also induced IRS2 up-regulation, but this treatment showed no change in insulin signaling unlike TSA. Thus, these results suggested that HDAC class I contributes to the expression of IRS2 and HDAC class II is associated with the decrease of insulin signaling. Among HDAC class II, we focused on HDAC6 because the expression of HDAC6 is particularly abundant in pancreatic islets. Interestingly, HDAC6 was observed specifically in pancreatic β-cells by the immunofluorescence staining in pancreatic sections of wild type mouse. Furthermore, decrease of insulin signaling was evident in MIN-6 cells treated with Tubacin, a HDAC6 specific selective inhibitor.

Conclusions: In pancreatic β-cells, the expression of IRS2 is suppressed by HDAC class I through histone deacetylation in promoter, while insulin signaling is up-regulated by HDAC6.

### 0114

### Evaluation of of immunogenicity of LY2963016 insulin glargine compared with insulin glargine in patients with type 2 diabetes mellitus

Therapeutics of Diabetes

J. Yeh, L. Ilag, M. Deeg, W. Huster, T. Costigan, J. Zielonka, R. Pollom, M. Prince and R. Konrad

*Eli Lilly and Company, IN, USA*

Objectives: LY2963016 (LY IGlar) is an insuline glargine product, with an identifical aminos acid sequence to insulin glargine (Sanofi-Aventis) (IGlar), demonstrating similar pharmacokinetic and pharmacodynamic profiles and no clinically meaningful differences from IGlar in patients with type 2 diabetes mellitus (T2DM). This study assessed the reponses and implications this might have for safety or efficacy.

© 2016 The Authors. Journal of Diabetes Investigation published by Asian Association for the Study of Diabetes (AASD) and John Wiley & Sons Australia, Ltd

METHODS: This was a 24-week, phase 3, randomized, double-blind, parallel study comparing the efficacy and safety of LY IGlar ($n$ = 376) and IGlar ($n$ = 380) in patients taking >=2 oral antihyperglycemic medications. Patients were insulin-naive (HbAlc: >=7.0% to <= 11.0%) or previously taking IGlar (HbAlc:=< 11.0%). Anti-insluin glargine antibodies (percentage binding) were measured for the overall 24-week treatment period. The relationships between anti-insuline glargine antibody levels or treatment-emergent antibody responses (TEARs) were evaluated.

RESULTS: Approximately 40% of patients entered the study taking IGlar, while 60% were insulin-naive. Similar proportions of patients completed the 24-week treatment period (LY IGlar 89%, IGlar 86%). The percentage of patients with detectable antibodies at baseline and throughout the treatment period were similar between groups ($P > 0.05$). There were no differences in TEAR incidence between treatment groups, and clinical outcomes (HbAlc, basal insulin dose, and total hypoglycemia) were not affected by TEAR status nor by insulin antibody levels ($P > 0.05$).

CONCLUSIONS: These clinical safety data demonstrate a highly similar immunogenicity profile of LY IGlar to IGlar, with no effects of anti-insulin glargine antibodies on selected efficacy and safety outcomes in patients with T2DM.

### 0116

### Mechanism of induction of incretin responsiveness by formation of pseudoislets

Insulin Secretion

N. Yokoi, M. Hashim, G. Gheni, R. Hoshikawa and S. Seino
*Kobe University Graduate School of Medicine, Kobe, Japan*

OBJECTIVE: Cell-cell interactions between pancreatic beta-cells and/or other cell types within islets are thought to be essential for normal regulation of insulin secretion. We previously reported that incretin responsiveness is drastically induced in incretin-unresponsive MIN6-K20 clonal beta-cells by formation of pseudoislets (PIs). In this study, we attempted to elucidate the mechanism of induction of incretin responsiveness by formation of PIs.

METHODS: PIs were formed from MIN6-K20 cells by culture on ultra-low attachment dishes for seven days. Electron microscopic analysis was performed on monolayer-cultured cells (MCCs), PIs, and mouse islets. Insulin secretion was measured by batch incubation method with stimulation of incretins such as glucagon-like peptide-1 (GLP-1) and glucose-dependent insulinotropic polypeptide (GIP). Metabolome analysis was performed by Capillary Electrophoresis/Mass Spectrometry (CE/MS) and Liquid Chromatography-tandem Mass Spectrometry (LC-MS/MS).

RESULTS: MCCs responded to glucose but not to incretins; formation of PIs drastically induced incretin responsiveness. Electron microscopic analysis showed that the number of insulin granules was increased and the mitochondria were well developed by formation of PIs. In the presence of GLP-1, glucose stimulated insulin secretion from PIs in a concentration-dependent manner. Conversely, in the presence of a high concentration of glucose, GLP-1 potentiated insulin secretion from PIs in a concentration-dependent manner. Insulin content in PIs was increased to a level similar to that in native islets. Metabolites of glycolysis, the TCA cycle, amino acids, and nucleic acids also were increased in PIs. We recently reported that glutamate produced from the malate-aspartate shuttle by glucose stimulation contributes to incretin responsiveness (Cell Rep, 2014). In the present study, aminooxyacetate (AOA), an inhibitor of this shuttle, was found not to affect the insulin secretory response to glucose but to virtually abolish the response to incretins in PIs.

CONCLUSION: Our results indicate that an increase in glutamate production through the malate-aspartate shuttle contributes to the induction of incretin responsiveness in PIs.

### 0117

### Inhibition of AGEs formation by national compounds and amelioration skin elasticity

Microvascular Complications

H. Sugawa[1], R.-I. Ohno[1], K. Maejima[2], M. Saigusa[2], M. Yamanaka[1], M. Nagai[1], M. Yoshimura[3], Y. Amakura[3] and R. Nagai[1]
[1]*Tokai University, Kumamoto, Aso-gun, Minamiaso, Kawayou, Japan,* [2]*Food Dev. Labs., Nippon Shinyaku Co., LTD, Kyoto, Minami-ku, Kisshoin'nishinoshomonguchi-cho, 14, Japan,* [3]*Col. of Pharm. Sci., Matsuyama University, Ehime, Matsuyama, Bunkyo-cho, 4-, Japan*

OBJECTIVES: Advanced Glycation End-products (AGEs) accumulate *in vivo* along with aging, with such accumulation being enhanced by aging-related diseases such as diabetic complications. It is known that preventive medicine is the most important approach to preventing the development of lifestyle-related diseases. We believe that the daily intake of AGEs inhibitors in natural products can play a beneficial role in preventing the pathogenesis of lifestyle-related diseases.

METHOD: We measured the inhibitory effect of natural compounds on pentosidine formation by incubation of ribose with gelatin in vitro, and pentosidine formation was measured by high performance liquid chromatography (HPLC). The study was conducted in accordance with the Declaration of Helsinki as a statement of ethical principles for medical research involving human subjects, including research on identifiable human material and data. The experimental protocol was approved by the ethics review committee of Kitahorie Hospital for intervention trials, and AGE analysis was approved by the ethics review committee of Tokai University (#14036). This study was conducted as an open trial which recruited healthy women between the ages of 32 to 48 years old. All subjects provided written informed consent. The median fasting blood glucose and hemoglobin A1c of the subjects were 83.6 ± 4.6 mg/dL and 5.25 ± 0.21, respectively. Two tablets (100 mg WEM in total), once a day, were administered to each subject and continued for 12 weeks. Routine blood and skin function testing were evaluated every 4 weeks.

RESULTS: Hot water extract of Mangosteen (Garcinia mangostana L.) pericarp (WEM) significantly inhibited pentosidine formation during gelatin incubation with ribose. Several compounds purified from WEM, such as garcimangosone D and rhodanthenone B, were identified as inhibitors of pentosidine formation. WEM was also administered to human healthy subjects, and measured serum pentosidine level by HPLC. As results, WEM significantly inhibited pentosidine formation. Oral administration of WEM reduced serum pentosidine contents. Furthermore, the moisture of the skin was also ameliorated by WEM.

CONCLUSIONS: The results demonstrated that intake of WEM reduced the glycation stress and resulted in the improvement of skin moisture. Therefore, intake WEM should be considered as a new food for preventing the accumulation of AGEs and improving functional disorders due to aging.

### 0118

### NOS1AP potentiates glucose stimulated-insulin secretion and protects against glucolipotoxicity-induced er stress in beta-cells

Insulin Secretion

Y. Zhao[1,2,3], D. C. Yin[1,2,3], K. D. Mu[1,2,3], C. R. Wang[1,2,3], C. Wang[1,2,3] and W. P. Jia[1,2,3]
*Shanghai Key Laboratory of Diabetes Mellitus, Shanghai Jiao Tong University Affiliated Sixth People's Hospital, Shanghai, China, Shanghai Diabetes Institute, Shanghai Jiao Tong University, Shanghai, China, Department of Endocrinology, Shanghai, China*

OBJECTIVES: Nitric oxide synthase associated protein (NOS1AP) is an adaptor protein regulating NO production through interaction with nNOS. The nNOS was reported to be associated with insulin release via reducing the intracellular calcium concentrations by inhibition of the inward $Ca^{2+}$ current through voltage-dependent $Ca^{2+}$ channels. We previously reported that NOS1AP single nucleotide polymorphism rs12742393 was associated with type 2 diabetes. In the present study we aimed to investigate the effect of NOS1AP on beta-cell function.

METHODS: INS-1 cells were transfected with recombinant adenoviruses encoding NOS1AP (Ad- NOS1AP), or EGFP. GSIS was performed in INS-1 cells transfected with Ad- NOS1AP. ER stress in INS-1 cells was induced with either

© 2016 The Authors. Journal of Diabetes Investigation published by Asian Association for the Study of Diabetes (AASD) and John Wiley & Sons Australia, Ltd

25 mM glucose or 500 mM palmitate for 16 h. Insulin levels were measured by ELISA assay and protein expression of phosphorylated eukaryotic translation initiation factor 2a (p-eIF2a), activating transcription factor 4 (ATF4), and C/EBP homologous transcription factor (CHOP) were detected using western blot analysis.

RESULTS: NOS1AP was highly expressed in human islets. 16.7 mmol/L glucose induced 2 h insulin secretion increased significantly in INS-1 cells tranfected with NOS1AP than that of control group (GFP group) ($P < 0.05$). This NOS1AP effect on glucose induced insulin secretion was abolished when cells were pretreated with verapamil (100 μM), an L-type calcium channel blocker. As expected, exposure of INS-1 cells to 25 mM glucose or 500 mM palmitate for 16 h up-regulated protein levels of p-eIF2a, ATF4, and CHOP compared with control cells. Overexpression of NOS1AP in INS-1 cell eliminated this increase, suggesting a role of this protein in regulating beta-cell ER homeostasis.

CONCLUSION: Our data demonstrate that NOS1AP plays a role in regulating beta-cell cells through potentiating GSIS and maintenance of ER homeostasis, which implicates NOS1AP as a therapeutic target for treatment of diabetes.

### 0119

### Thyroid stimulating hormone is independently associated with diabetic peripheral neuropathy in type 2 diabetes patients

Microvascular Complications

W. Zhao, F. Liu, F. Liu, C. Pang and W. Jia

*Shanghai Key Laboratory of Diabetes, Department of Endocrinology & Metabolism, Shanghai Jiao-Tong University Affiliated Sixth People's Hospital, Shanghai Clinical Medical Center of Diabetes, Shanghai, Shanghai, China*

OBJECTIVES: The association between thyroid stimulating hormone (TSH) and type 2 diabetes mellitus(T2DM)is well known. However, whether TSH is related to diabetic peripheral neuropathy (DPN) has not been studied. The aim of this study was to explore the relationship between TSH and DPN in Chinese patients with T2DM.

METHODS: In this cross sectional study, 605 patients with T2DM were enrolled. Subjects were divided into three groups: DPN, signs of DPN and non-DPN group. Serum TSH concentration was measured by chemiluminescence immunoassay. Subclinical hypothyroidism (SCH) was defined as an elevated TSH level (>4.0 mIU/L) and a normal free thyroxine (FT4) level. DPN was evaluated by neurological symptoms, neurological signs and electromyogram.

RESULTS: Serum TSH levels were significantly higher in DPN[2.42(1.53–4.52)mIU/L] and signs of DPN(2.75(1.74–3.83)mIU/L)patients compared with non-DPN T2DM patients [1.65(1.08–2.4)mIU/L] (both $P < 0.01$).The prevalence of DPN and signs of DPN in the SCH group was higher than in the euthyroid group (14.09% *vs.* 35.63%; 19.85% *vs* 37.93%, both $P < 0.01$). Spearman's correlation analysis showed that serum TSH level was positively associated with DPN(r=0.172, $P < 0.01$). A significant independent association between TSH and DPN was found by multiple logistic regression analysis after adjusted for potential confounding variables [odds ratio(OR)=1.365, $P < 0.01$]. The patients were sequentially assigned to quartiles according to the TSH level. Compared with TSH quartile 1 (referent), patients in quartile 2 [OR=1.862; 95% confidence interval (CI), 1.3–2.668; $P < 0.01$], quartile 3 (OR=1.617; 95%CI, 1.123–2.330; $P = 0.01$) and quartile 4 (OR=3.799; 95%CI, 2.677–5.391; $P < 0.01$) had higher risk of DPN. Receiver-operating characteristic curve (ROC) analysis revealed that the optimal cutoff point of TSH to indicate DPN was 3.045 mIU/L in male patients and 2.94 mIU/L in female patients.

CONCLUSIONS: There was a strong and independent association between TSH and DPN in Chinese population with T2DM. High serum TSH level may be a potential risk factor for DPN.

### 0120

### The indicating value of lower extremity artery disease screening for the cardiovascular disease in type 2 diabetes patients

Macrovascular Complications

X. Hua, Y. Hu, J. Zhao, J. Zhao, H. Zeng, L. Li, F. Liu, Y. Bao and W. Jia

*Department of Endocrinology and Metabolism, Shanghai Jiaotong University Affiliated Sixth People's Hospital, Shanghai Clinical Medical Center of Diabetes, Shanghai Key Clinical Center, Shanghai, China*

OBJECTIVE: This study is to clarify whether lower limb artery disease screening has indicating value for the diagnosis of CAD by analyzing the association between lower extremity vascular disease and coronary vascular disease(CAD) in patients with type 2 diabetes mellitus(T2DM).

METHODS: A total of 268 hospitalized patients with type 2 diabetes were recruited, and their clinical data and biochemical parameters are collected. All patients underwent coronary CT angiography (CTA) scan, vascular ultrasound and ankle-brachial index (ABI) measurement. The ABI <0.9 was defined as the diagnosis of lower limb ischemia (LLI). The vascular stenosis is more than 50% shown by CTA, or a history of percutaneous coronary intervention or percutaneous transluminal coronary angioplasty were considered as CAD. According to ABI values, patients were divided into three groups: ABI <0.9 ($n = 25$), ABI 0.9–1.3 ($n = 207$) and ABI > 1.3 ($n = 36$). The prevalence of CAD and its oddis ratio were analyzed.

RESULTS: The prevalence of CAD in ABI<0.9 group (82.5%) was higher significantly than that of normal ABI group(34.7%) and high ABI group(40%, both $P < 0.01$). The occurrence of CAD in LLI group was also significantly higher than non-LLI group (79.66% *vs.* 56.19%, $p < 0.01$). In male patients, the risk of CAD in LLI group increases 1.4 times than non-LLI group (OR = 2.411, 95% CI = 1.018–5.558, $p < 0.05$). In female patients, the risk increased more than 20 times (OR = 22.3, 95% CI = 1.394–11.034, $p < 0.01$). According to OR analysis of LLIfor CAD, it showed that the risk of CAD was 5.5-fold increase by LLI (OR = 6.5, 95% CI 2.08–20.30, $P < 0.001$).

CONCLUSIONS: Rather than ultrasound instrument, low ABI is associated with CAD in type 2 diabetes patients. The risk of CAD increases 5.5 times with lower limb ischemia.

### 0121

### Results of phase III trials suggest the difference of SGLT2/ SGLT1 inhibition between dapagliflozin and canagliflozin in Japanese type 2 diabetes patients

Therapeutics of Diabetes

Y. Suzuki

*HDC Atlas Clinic, Tokyo, Japan*

A phase III trial of dapagliflozin (52 weeks) in Japanese Type 2 diabetes (T2DM) patients indicates a significant decrease of HbA1c in combination with alpha-glucosidase (a-GI), thiazolidinedione (TZD), or glinide (GLD). The reduction was –0.81% with a-GI, –0.86% with TZD, and –0.76% with GLD. The data suggest that dapagliflozin has synergistic effect with post prandial glucose regulator like a-GI and GLD, and with insulin sensitizer like TZD (1).

A phase III trial of canagliflozin (52 weeks) in Japanese T2DM patients indicates a significant reduction of HbA1c in combination with DPP-4 inhibitor (DPP-4i), TZD, or GLD. The reduction was −1.04% with DPP-4i and TZD, and −1.06% with GLD. Compared with dapagliflozin, canagliflozin seems to have more synergistic effect with DPP4i than with a-GI in reducing HbA1c (2).

Canagliflozin has a suppressive effect against postprandial glucose, compared with dapagliflozin (3). Theoretically, in the case of canagliflozin, because of low-potency SGLT1 inhibition, the non-renal (intestinal) effect may also be considered, which may contribute to better control of postprandial hyperglycaemia. In literature, so far, this contribution remains to be better analyzed in humans. However, when we look at the results of phase III trials in Japanese T2DM patients, the difference seems to be apparent.

Namely, because of low-potency SGLT1 inhibition, canagliflozin has an effect to increase plasma active GLP-1 concentration (4). As for GLP-1, miglitol is well

© 2016 The Authors. Journal of Diabetes Investigation published by Asian Association for the Study of Diabetes (AASD) and John Wiley & Sons Australia, Ltd known to enhance GLP-1 secretion by delaying the absorption of glucose in upper intestine (5). Therefore, canagliflozin has both SGLT2i effect plus non-renal (intestinal) effect like miglitol, which can induce the anorectic effects (similarly to incretin-based therapies), leading to better glycemic control. As is shown in a phase III trial of canagliflozin, the beneficial synergistic effect with DPP4i suggests that canagliflozin has a-GI-like anorectic effects similarly to miglitol. In addition, the less reduction of HbA1c by canagliflozin when administered with a-GI suggests that the intestinal effect of calagliflozin offsets the synergistic effect of a-GI.

In conclusion, phase III trials of dapagliflozin and canagliflozin suggest the importance of their difference of SGLT2/SGLT1 inhibition in humans. The difference is obviously indicated as we compare the synergistic and offset effect when they are administered with a-GI and with DPP4i.

REFERENCES

1. Diabetes Ther 5:415–33, 2014
2. J Diabetes Investig 6:210–8, 2015
3. Diabetes Obes Metab 17:188–97, 2015
4. J Pharmacol Sci 127:456–61, 2015
5. Diabet Med 26:187–8, 2009

### 0124

### Major increases in morning glucose levels may predict nocturnal hypoglycemia in type 2 diabetes

Epidemiology

S. Takeishi, A. Mori, M. Kawai, Y. Yoshida, H. Hachiya, T. Yumura, S. Ito, T. Shibuya, N. Fushimi, N. Ohashi and H. Kawai

*Ichinomiya nishi Hospital, Ichinomiya-city, Aichi, Japan*

OBJECTIVES: Although a relationship between nocturnal hypoglycemia and sudden death has been suggested, predicting nocturnal hypoglycemia is challenging in patients with type 2 diabetes, as many are unaware of their condition, especially advanced or elderly patients. Nocturnal hypoglycemia may be associated with increased morning glucose levels; however, the mechanism of this relationship remains unknown. We therefore studied the association between morning glycemic variability and nocturnal hypoglycemia in patients with type 2 diabetes using continuous glucose monitoring (CGM) data to determine if morning glucose levels can predict nocturnal hypoglycemia.

METHOD: We retrospectively analyzed 106 patients with type 2 diabetes who underwent CGM during admission. The parameters used as indices for glycemic variability included pre-breakfast glucose level (Pre-breakfast), highest postprandial glucose level within 3 h after breakfast (Highest level), time from start of breakfast to the highest postprandial glucose level (Highest time), difference between pre-breakfast and highest postprandial breakfast glucose levels (Increase), area under the curve (AUC) (≥180 mg/dL) for glycemic variability within 3 h after breakfast (Morning AUC), post-breakfast glucose gradient (Gradient), and the increased glucose level/pre-breakfast glucose level ratio (Increase/Pre-breakfast). We analyzed the associations between these factors (explanatory variables) and nocturnal hypoglycemia (<70 mg/dL from 0:00 to 8:00) (response variable) using logistic regression analysis. Receiver operating characteristic (ROC) analysis was used to determine optimal cut-off values to predict nocturnal hypoglycemia (Hypoglycemia).

RESULTS: Twenty-eight patients had hypoglycemia (26.4%). In univariate analysis, Pre-breakfast, Highest level, Highest time, Increase, Morning AUC, and Increase/Pre-breakfast were significantly associated with Hypoglycemia. After stepwise multivariate adjustment using these parameters as explanatory variables, Pre-breakfast, Highest level, Increase and Increase/Pre-breakfast were independently significantly associated with Hypoglycemia (*odds ratio [OR] 0.97 (95% confidence interval [CI] 0.95–0.998) P = 0.03, OR 1.07 (95% CI 1.01–1.12) P = 0.02, OR 1.12 (95% CI 1.01–1.24) P = 0.03, and OR 1.06 (95% CI 1.01–1.12) P = 0.03*, respectively). The cut-off values were: Pre-breakfast: 123 mg/dL, sensitivity 0.89, specificity 0.55, AUC 0.78 (*95% CI: 0.68–0.87, P < 0.0001*); Increase: 90.5 mg/dL, sensitivity 0.75, specificity 0.64, AUC 0.76 (*95% CI: 0.65–0.86, P < 0.0001*); and Increase/Pre-breakfast: 90.2%, sensitivity 0.75, specificity 0.76, AUC 0.78 (*95% CI: 0.69–0.87, P < 0.0001*).

CONCLUSIONS: Major increases in morning glucose levels were associated with and may predict nocturnal hypoglycemia in patients with type 2 diabetes. Considering the risk for nocturnal hypoglycemia, treatments should be adjusted in patients with pre-breakfast glucose levels <120 mg/dL, increased glucose levels ≥90 mg/dL, and increase/pre-breakfast ratios ≥90%.

### 0126

### Association of increased body mass index and avascular necrosis of femoral head

Obesity

A. Lim-Teodoro[1], C. Clado-Reyes[1] and H. Paulino[2]

*[1]World Citi Medical Center, Quezon City, The Philippines, [2]Wellton Hospital, Seoul, Republic of Korea*

BACKGROUND/OBJECTIVE: The prevalence of obesity has been increasing over the past several decades and has drawn interest in recent studies because of its modifiable nature. Recent studies showed that there is an increase likelihood of total joint arthroplasty in patients who are overweight and obese due to osteoarthritis. This study investigates if being overweight and obese can lead to total hip arthroplasty due to death of bone cells known as avascular necrosis. The aim of this study is to determine the prevalence of increased body mass index among patients with avascular necrosis. We further determine if being overweight and obese is associated with avascular necrosis.

METHODS: This is a retrospective cross sectional study. Anthropometric data were gathered and body mass index were calculated. Overweight and obese are classified according to the cut off guidelines based on the race of origin. Patients were classified as underweight if their BMI was <18.5, normal weight if it was 18.5 to 22.9, overweight if it was 23.0 to 24.9, and obese if it was >25.

RESULTS: There are 284 patients with avascular necrosis of the femoral head who underwent total hip arthroplasty from January to December 2014 were included in the study. One hundred eighty two patients were categorized as being overweight and obese with a prevalence rate of 64%. Significant association was noted for increasing weight and body mass index of >23 in patients with avascular necrosis of femoral head as proven by a p value of <0.0001.

CONCLUSION: The study revealed a high prevalence of overweight and obesity among patient with avascular necrosis. It was also noted that increased weight and body mass index is associated with avascular necrosis. Considering the growing incidence of overweight and obesity in the world and the potential link eventually to avascular necrosis, control of body weight must be addressed and awareness of this condition could lead to better patient outcome. Overweight and obesity is a modifiable condition. Hence, early diagnosis and appropriate intervention can prevent or delay the need for future joint replacement.

### 0128

### The relationship of circulating lipocalin-2 levels and incident cardiovascular events in subjects with or without type 2 diabetes — a 7-year prospective study

Macrovascular Complications

G. Wu[1], H. Li[1], Q. Fang[1], S. Jiang[1], L. Zhang[1], J. Zhang[1], X. Hou[1], J. Lu[1], Y. Bao[1], A. Xu[2] and W. Jia[1]

*[1]Shanghai Jiao Tong University, Shanghai, China, [2]University of Hong Kong, Hong Kong*

OBJECTIVES: In diabetic patients, cardiovascular disease (CVD) was the most common underlying cause of death. Adipose tissue inflammation and perturbation of adipokine secretion may contribute to the pathogenesis of CVD. Among the adipokines, Lipocalin-2 (LCN2) was shown to be positively associated with CVD in cross-sectional studies. We aimed to evaluate the prospective association of LCN2 with CVD involving a population-based cohort comprising 770 Chinese subjects with up to 7 years follow-up.

METHOD: Serum LCN2 levels were measured using ELISA. Independent predictors of CVD development were identified using Cox proportion hazards regression. The predictive performances of the various models were assessed by Kaplan-Meier analysis.

RESULTS: Serum LCN2 level was increased in subjects who had CVD at baseline. Among those without baseline CVD, 31 subjects with baseline T2DM (29.0% of the total subjects with baseline T2DM) and 67 subjects without baseline T2DM (11.2% of the total subjects without baseline T2DM) developed CVD during follow-up. LCN2 was a predictor of CVD development during 7-year follow-up in men, which remained statistically significant after adjustment for traditional cardiovascular risk factors (hazard ratio: 1.038 [95%CI: 1.017–1.060]) and high sensitive C-reactive protein (hsCRP). Kaplan-Meier analysis suggested combination

© 2016 The Authors. Journal of Diabetes Investigation published by Asian Association for the Study of Diabetes (AASD) and John Wiley & Sons Australia, Ltd of LCN2 and hsCRP might improve the prediction of CVD events in male subjects. When subjects with or without T2DM at baseline were analyzed separately, baseline LCN2 levels in male subjects without T2DM who developed CVD events during follow-up (19.5 μg/L [12.9–32.0]) had the trend to be higher than those who did not develop CVD events (17.1 μg/L [11.8–24.5], $P = 0.060$), although the difference was not statistically significant. In subjects with T2DM at baseline, LCN2 levels in male subjects who developed CVD events (22.7 μg/L [18.2–38.8]) also had the trend to be higher than those who did not develop CVD events (18.0 μg/L [12.3–25.8], $P = 0.057$). However, such relationship between baseline LCN2 and CVD development was not seen in female subjects.

CONCLUSIONS: LCN2 level is an independent predictor of CVD events in men in a population-based cohort, irrespective of the presence or absence of baseline T2DM. Measurement of serum LCN2 might be useful for early detection and intervention of CVD, especially for people with T2DM due to higher incidence of CVD among those patients.

### 0130

### Attenuation of streptozotocin-induced pancreatic beta cell death in transgenic fat-1 mice via autophagy activation

Islet Biology

D.-M. Lim, W.-M. Hwang, K.-Y. Park and S.-Y. Han

*Department of Internal Medicine, Konyang University School of Medicine, Daejon, Republic of Korea*

BACKGROUND: Diabetes incidence has rapidly increased owing to changes in eating habits. Inflammatory factors and beta (β) cell dysfunction due to high-fat diets aggravate chronic diseases and complications. However, omega-3 dietary fats have anti-inflammatory effects, and autophagy involvement in diabetes etiology has been reported. Therefore, we aimed to examine protective effects of autophagy on diabetes, using fat-1 transgenic mice with omega-3 self-synthesis capability.

METHODS: Streptozocin (STZ) administration induced β cell dysfunction in mice, we then measured blood glucose levels and water consumption. Via H&E and Masson's trichrome staining we quantitatively assessed STZ-induced changes in the number, mass, and fibrosis of pancreatic islets of fat-1 and control mice. We identified the microtubule-associated protein 1A/1B light chain 3-immunoreactive puncta in β cells and quantified p62 levels in the pancreas of fat-1 and control mice.

RESULTS: STZ-induced diabetic phenotypes, including hyperglycemia and polydipsia, were significantly attenuated in fat-1 mice. Histological determination using H&E and Masson's trichrome staining revealed protective effects of the fat-1 mutation on cell death and scarring of pancreatic islets after STZ-injection. In β cells of control mice, autophagy was abruptly activated after STZ-treatment. Basal autophagy levels were elevated in fat-1 mice β cells, which persisted after STZ-treatment. Interestingly, analysis of p62 demonstrated that STZ-induced impairment of autophagic clearance was significantly reversed in fat-1 mice pancreas.

CONCLUSIONS: Fat-1 transgenic mice with n-3 PUFA self-synthesis capability have protective effects against STZ-induced β cell death, and n-3 PUFA had protective effects against diabetes, through activating autophagy in β cells.

### 0131

### The effects of homocysteine and hyperglycemia on the proliferation of aortic vascular smooth muscle cells of genetically determined obese type 2 diabetes rat

Macrovascular Complications

S. H. Yu[1], S.-H. Kim[2], H. Y. Chung[3], J. M. Yu[1], H. J. Yoo[1], H. Oh[4], J. H. Park[4] and D. S. Kim[4]

*[1]Division of Endocrinology and Metabolism, Hallym University College of Medicine, Seoul, Republic of Korea, [2]Division of Endocrinology & Metabolism, Cheil General Hospital & Women's Healthcare Center, Dankook University College of Medicine, Seoul, Republic of Korea, [3]Division of Endocrinology and Metabolism, Kyung Hee University College of Medicine, Seoul, Republic of Korea, [4]Division of Endocrinology and Metabolism, Hanyang University College of Medicine, Seoul, Republic of Korea*

OBJECTIVES: Hyperhomocysteinemia is recognized as a independent risk factor for the development of atherosclerosis in type 2 diabetes mellitus. The aim of this study was to investigate the role of homocysteine and hyperglycemia on the proliferation of rat aortic vascular smooth muscle cells (VSMCs).

METHODS: Rat aortic VSMCs were isolated from the Otsuka Long-Evans Tokushima Fatty (OLETF) by enzyme methods. The VSMCs were incubated in the presence of homocysteine with/without PD98059, and wortmannin for 48 h in different concentrations of glucose. Then the proliferation was evaluated by a methylthiazoletetrazolium (MTT) and flow cytometric analysis (FACS) assay. Western blot analysis was performed using phospho-p44/42 mitogen-activated protein (MAP) kinase antibody, phosphoinositide 3-kinase (PI3) kinase and NF-κB antibody. And apoptotic pathway were analyzed.

RESULTS: We found enhanced proliferation of VSMCs incubated with high glucose medium compared to normal glucose media using the methylthiazoletetrazolium assay. And this proliferation of VSMCs were more enhanced with homocysteine. In western blot analysis, immunoexpression of phospho-p44/42 MAP kinase by homocysteine was significantly increased in a dose-dependent manner ($P < 0.05$). VSMC proliferation and immunoexpression of phospho-p44/42 MAP kinase antibody and PI3 kinase antibody were significantly increased by homocysteine at various concentrations of glucose ($P < 0.05$). In the apoptotic pathway, Bcl-xl, phospho-Bad, and caspase-3 were not affected

CONCLUSIONS: These results suggest that the MAP kinase and PI3 kinase pathway may be one of the main mechanisms involved in rat VSMC proliferation caused by homocysteine and hyperglycemia.

### 0132

### Synthetic reprogramming factor may increase the efficiency of the generation of iPS cells

Genetics and Epigenetics

F. Liu[1], B. Feng[2], Y. Bao[1], W. Lu[2], W. Zhao[1], S. Chen[1], W. Xu[1], Q. Li[1], J. Hu[3] and W. Jia[1]

*[1]Department of Endocrinology and Metabolism, Shanghai Jiao Tong University Affiliated Sixth People's Hospital, Shanghai Clinical Center for Diabetes, Shanghai Diabetes Institute, Shanghai Key Laborator, Shanghai, China, [2]Department of Endocrinology, the Affiliated East Hospital of Tongji University, Shanghai, China, [3]Department of Medicine, VA Palo Alto Health Care System, Stanford University Medical School, Palo Alto, USA*

Somatic cells can be reprogrammed into induced pluripotent stem cells (iPSCs) by defined transcription factors Oct4, Sox2, c-Myc and Klf4 or Lin28, Which allow creation of patient- and disease- specific stem cells. However the low efficiency and slow kinetics of the reprogramming process have hampered progress with this technology. Here we hypothesize the transcription role of transcription factor Oct4 is weak. So we use a stronger transcription promoter VP16 linking with Oct4 binding domain to construct new synthetic reprogramming factors (sRFs) in order to increase the efficiency of the generation of iPSCs.

OBJECTIVES: To construct synthetic reprogramming factor (sRF) and preliminary evaluate the efficiency of these synthetic reprogramming factors (sRFs) in the generation of induced pluripotent stem cells (iPS cells).

METHODS: We rebuilt the transcription factor Oct4 by introducing the potent transcriptional domain Viral protein 16 (VP16) into synthetic reprogramming factors using molecular cloning technique. And then we checked them by cell transfection and gene sequence. To evaluating the role of sRF, we measured the activity of luciferase of firebird and renilla by luciferase assay system. Also we investigated the target genes expression of Oct4 gene after stimulating 293T cells of sRFs by reverse transcription-polymerase chain reaction (RT-PCR).

RESULTS: (1) We successfully constructed five recombinant plasmids named IVO, IVOF, OCT, ORT, TVO, respectively. After transfected 293T cells, we observed green fluorescence by fluorescence microscope, which show these five sRFs could be normally expressed in 293T cells. (2) The sRF-IVO and sRF-TVO are much stronger than the control sRF-OCT, but sRF-IVOF and sRF-ORT are much weaker than sRF-OCT. (3) The expressions of pluripotency marker genes such as Oct4, Nanog and Sox2 increased after stimulating by sRF-IVO and sRF-TVO respectively.

CONCLUSIONS: Classic stem cell transcription factor Oct4 can be reconstructed into stronger factor sRF by fusing VP16. The sRFs (IVO and TVO) may increase the generation of iPS cells.

© 2016 The Authors. Journal of Diabetes Investigation published by
Asian Association for the Study of Diabetes (AASD) and John Wiley & Sons Australia, Ltd

### 0133

### A retrospective review of the use of adrenal venous sampling in a regional hospital and the development of clinical prediction score to diagnose unilateral primary aldosteronism

Pituitary and Adrenal Disorders

C. T. Chung and Y. F. E. Lau
*Department of Medicine, Pamela Youde Nethersole Eastern Hospital, Hong Kong, Hong Kong*

Background: Subtype classification of primary aldosteronism is important in view of the potential curability of unilateral disease by adrenalectomy. Adrenal venous sampling is recommended to be the gold standard for this purpose. However, this procedure was invasive, poorly standardized, and not widely available.

Objectives: The aims of this study are to review the use of adrenal venous sampling in a regional hospital, and to identify patients' characteristics that can predict unilateral aldosterone hypersecretion, with a view to select out some patients in whom AVS can be exempted.

Methods: Patients with primary aldosteronism who had undergone adrenal venous sampling for subtype classification from July 2007 to December 2014 in a regional hospital were included. Medical records were reviewed and analysed retrospectively. The baseline clinical features, biochemical parameters and imaging result of the patients together with the success rate of adrenal venous sampling were reviewed. Clinical variables independently associated with a lateralized adrenal venous sampling in multivariate logistic regression were used to derive a clinical prediction score.

Results: Total 92 AVS had been performed in 91 patients from July 2007 to December 2014. 47 AVS (51.1%) were conclusive using pre-defined criteria while 45 AVS (48.9%) were inconclusive. Failure in cannulation of the adrenal veins was the most common cause of inconclusive AVS (40 out of 45, 89%). AVS had good accuracy in diagnosing unilateral form of primary aldosteronism in terms of post-surgery improvement or cure of hypertension, normalization of potassium levels and the aldosterone-renin ratio.

Based on the adrenal venous sampling result, 21 and 26 patients were diagnosed to have unilateral and bilateral forms of primary aldosteronism respectively,. A clinical prediction score for predicting unilateral form of primary aldosteronism was built based on the regression coefficients of the following three variables: 1) potassium supplement requirement, 2) aldosterone-renin ratio and 3) presence of adrenal adenoma or thickening in CT adrenals. ROC curve analysis for the ability to diagnose unilateral primary aldosteonism showed a score of ≥6 points had 76.2% sensitivity and 92.3% specificity, score of ≥4 points had 100% sensitivity and 46.2% specificity, and a score of 8 points had 19% sensitivity and 100% specificity. The area under the ROC curve was 0.907 (95% confidence interval 0.823–0.990).

Conclusion: AVS predicts good surgical outcome accurately in patients with unilateral primary aldosteronism. The clinical prediction score developed could discriminate between unilateral and bilateral primary aldosteronism and is useful for selecting patients in whom AVS can be exempted.

### 0135

### Impact of genetic variants identified in genome-wide association studies of diabetic retinopathy in Chinese patients with type 2 diabetes

Genetics and Epigenetics

C. Y. Cheung[1], E. Y. Hui[1], K. H. Kwok[1], Y.-C. Woo[1], P. C. Lee[1], W.-S. Chow[1], R. Gangwani[2], C. H. Fong[1], M. M. Yuen[1], R. L. Wong[1], A. Xu[1], D. S. Wong[2], P.-C. Sham[3] and K. S. Lam[1]
[1]*Department of Medicine, The University of Hong Kong, Hong Kong,* [2]*Department of Ophthalmology, The University of Hong Kong, Hong Kong,* [3]*Department of Psychiatry, The University of Hong Kong, Hong Kong*

Objectives: Diabetic retinopathy (DR) is the most common microvascular complication of type 2 diabetes (T2DM). Novel DR-susceptibility loci have been identified from recent genome-wide association studies (GWAS) in various populations. The major objective of this study was to examine the association of these newly identified DR-associated single nucleotide polymorphisms (SNPs) with DR in Chinese patients with T2DM.

Methods: A cross-sectional case-control study on sight-threatening DR (STDR) based on participants from the Hong Kong West Diabetes Registry was performed. STDR cases were defined as T2DM patients with either proliferative DR (PDR) or pre-PDR, or with clinically significant macular oedema. Non-DR controls were T2DM patients without retinopathy. We genotyped 36 SNPs showing top association signals with DR in previous GWAS in 1490 non-STDR controls and 567 STDR cases, including 309 subjects with PDR. Multiple logistic regression models with adjustment for confounding factors, including gender, age, duration of diabetes, and the presence of hypertension, were employed to examine for the associations with STDR or PDR.

Results: SNP rs2115386 of the insulin receptor gene (*INSR*) showed the strongest association with DR: $P_{adjusted} = 9.13 \times 10^{-4}$ (OR[95%CI]: 1.28[1.11–1.48]) for STDR, and Padjusted = $1.12 \times 10^{-4}$ (OR: 1.44[1.20–1.74]) for PDR. An intergenic SNP, rs4462262, located between *ZWINT* and *MRPS35P3* ($P_{adjusted} = 0.041$; OR: 1.38[1.01–1.89]) and rs599019 located downstream of *COLEC12* ($P_{adjusted} = 0.019$; OR: 1.19[1.03–1.38]) also showed marginal associations with STDR, but not with PDR alone. On the other hand, rs10199521 (*MYT1L-LOC729897*; $P_{adjusted} = 0.022$; OR: 1.25[1.03–1.51]), and rs899036 (*API5*; $P_{adjusted} = 0.049$; OR: 1.36[1.00–1.85]) were independently associated only with PDR. Similar results were observed when glycated hemoglobin (HbA1c) was also included in the adjustment models. In combined genetic risk analyses, the combined genetic risk score (GRS) constructed with the significant SNPs showed an OR of 1.24 ($P_{adjusted} = 1.20 \times 10^{-5}$) and 1.35 ($P_{adjusted} = 2.00 \times 10^{-6}$) for each additional risk allele with STDR and PDR, respectively.

Conclusion: We have successfully confirmed the significant associations between severe DR (STDR and/or PDR) and several genetic variants identified in previous GWAS. The highly significant combined GRS suggest potential applications in risk stratification for enhanced DR screening. Our findings on rs2115386 of the insulin receptor gene *INSR* have provided supporting evidence for a role of insulin resistance, or the compensatory hyperinsulinaemia, in the pathogenesis of DR.

### 0136

### The intergenerational differences of biochemical characteristics in korean patients with newly diagnosed type 2 DM

Epidemiology

J. H. Park, H. Oh, W. H. Choi, Y. H. Ahn and D. S. Kim
*Department of Internal Medicine, College of Medicine, Hanyang University, Seoul, Republic of Korea*

Objective As the prevalence of obesity in Korea has increased, the incidence of type 2 diabetes (T2DM) in younger generation has increased. This study was aimed to investigate the generational difference of Korean patients with newly diagnosed T2DM.

Methods In this study, 129 patients, who were newly diagnosed with T2DM, were included. Patients were divided into 3 groups according to onset age as follows: Group I ($n = 26$), ≤ 39; Group II ($n = 68$), ≥ 40 and ≤ 59; and Group III ($n = 35$), ≥ 60.

Results Group I had mean value of higher height (171.8 ± 8.2 cm, $P < 0.001$) and larger weight (86.5 ± 24.5 kg, $P < 0.001$). Body mass index was higher in Group I (29.0 ± 6.6 kg/m$^2$, $P < 0.001$) compared with other groups. Glycated hemoglobin was also higher in Group I (10.3 ± 2.6%, $P < 0.001$) compared with the other groups. HOMA-IR tended to be higher in Group I (5.3 ± 3.7) compared with the other groups. However, HOMA-beta tended to be lower in Group III (42.0 ± 25.5%) compared with the other groups. There was no significant difference in the presence of hypertension and dyslipidemia in each group.

Conclusion This study shows that younger generation with newly diagnosed T2DM is associated with higher body mass index and has increased insulin resistance.

### 0137

### Bisphenol compounds modulate estrogen receptor but not wnt-signaling pathway in human endometrial epithelial (Ishikawa) cell and suppress spheroid attachment

Endocrine Disruptors

H. Fan, W. Yueng and C. Lee
*The University of Hong Kong, Hong Kong*

Background: Bisphenol A (BPA) is one of the well-known chemicals with weak estrogenic activity that might affect our endocrine function, and therefore, named

© 2016 The Authors. Journal of Diabetes Investigation published by
Asian Association for the Study of Diabetes (AASD) and John Wiley & Sons Australia, Ltd as endocrine disrupting chemicals (EDCs). Due to the potential health hazardous of BPA onto our bodies, BPA is being substituted with Bisphenol S (BPS) and BIsphenol F (BPF). Yet, their effects on human health and reproduction remain largely unknown.

Objectives: To study the effects of bisphenol compounds on cell proliferation, expression of estrogen receptors (ERs) and molecules of Wnt-signaling pathway in human endometrial Ishikawa cell and on spheroid attachment.

Methods: Cell proliferation and trypan blue assays were used to study the effects of bisphenol compounds on Ishikawa cells proliferation. The expression of steroid receptors and molecules of Wnt-signaling pathway were quantified by Western Blotting. Dual Luciferase Assay was used to compare the effect of bisphenol compounds on the activation ERE-luciferase activity. Spheroids-endometrial epithelial cells co-culture assay was used to assess the attachment potential of Jeg-3 spheroid (blastocyst surrogate) onto Ishikawa cells – an in vitro model to mimic embryo attachment in vivo.

Results: BPA and BPF at 100 μM inhibited Ishikawa cells proliferation at 24 h, and BPS at 100 μM inhibited cell proliferation at 48 h. Similarly BPA and BPF, but not BPS at 100 μM reduced total cell number by trypan blue at 24 h. BPA, BPS and BPF at 100 μM suppressed the expression of ERα, ERβ and GPR30. BPA and BPS at 10 μM suppressed the expressions of ERβ and GPR30; while BPF at 1 μM suppressed the expression of ERβ and GPR30. BPA and BPF at 100 μM suppressed expression of E-cadherin (cell adhesion molecule), but not molecules of Wnt-signaling pathway (GSK-3β and β-catenin). BPA and BPF at 1–10 μM and BPS at 10 μM activated the ERE-luciferase expression in transfected cells. The effect could be blocked by ICI 182780 (ERs antagonist) and MPP (ERα antagonist), but not by PHTPP (ERβ antagonist) or G15 (GPR30 antagonist). Bisphenols suppressed spheroids attachment at BPA 10–100 μM; while BPS and BPF only at 100 μM.

Conclusions: High concentrations of Bisphenol compounds inhibit Ishikawa cells proliferation, expression of ER receptors and E-cadherin, but not molecules of Wnt-signaling pathway. BPA, BPS and BPF exhibited weak estrogen activity, partly acting through the ERα. High concentration of Bisphenol compounds inhibits spheroid attachment onto Ishikawa cells suggesting a potential impact on embryo attachment in vivo. [The work is partly supported by GRF grant HKU17120415 to KFL]

### 0138

### Interactome analysis discovers of type 2 diabetes signatures complementary to GWAS loci

Genetics and Epigenetics

J. W. Li[1], H. M. Lee[2], K. Y. Yip[3,4], R. C. W. Ma[2,5], J. C. N. Chan[2,5] and T. F. Chan[1,5]

[1]*Hong Kong Bioinformatics Centre, School of Life Sciences, The Chinese University of Hong Kong, Shatin, N.T., Hong Kong,* [2]*Department of Medicine and Therapeutics, The Chinese University of Hong Kong, Prince of Wales Hospital, Shatin, N.T., Hong Kong,* [3]*Department of Computer Science and Engineering, The Chinese University of Hong Kong, Shatin, N.T., Hong Kong,* [4]*Division of Genomics and Bioinformatics, School of Biomedical Sciences, The Chinese University of Hong Kong, Shatin, N.T., Hong Kong,* [5]*Hong Kong Institute of Diabetes and Obesity, The Chinese University of Hong Kong, Shatin, N.T., Hong Kong*

Objective: GWAS have identified more than 80 loci associated with Type 2 Diabetes (T2D), but could not fully explain its heritability. In GWAS, each individual variant is treated as a testing unit although gene-gene interactions are likely to play significant roles in complex diseases. We aim to utilize transcriptome data to identify novel pathways implicated in T2D.

Method: In this study, we integrated transcriptome analysis of expression data from peripheral blood mononuclear cells (PBMC) in a case-control study (10 cases: 10 controls) to demonstrate insights of a widely studied complex disease could be obtained by discovering disease modules in a comprehensive tissue-specific interactome. We first devised a method to construct a comprehensive tissue-specific interactome, making use of both experimental validated interactome network and our Type 2 diabetes (T2D) case-control transcriptome sequencing data. We then identified interactome signatures possessing significant connections to T2D-GWAS genes and clusters of transcriptionally altered genes.

Results: Through the Interactome-transcriptome analysis, we identified a long tail of 420 genetic loci, termed T2D interactome (T2Di) signatures. The validity of the T2Di signatures was vigorously and systematically cross-examined with a series of large-scale datasets and public databases. According to data retrieved from the Roadmap Epigenomics Project, the signatures were enriched in T2D-affected cell type-specific regulatory elements, a phenomenon observed in diseases of which growth trajectory plays a major role. Based on data sourced from the Genotype-Tissue Expression Project (GTEx) and Multiple Tissue Human Expression Resources (MuTHER), our T2Di signatures were also enriched in eQTL loci. By integrating those *cis*- and *trans*- eQTL signals, we showed that the T2Di signatures were enriched in T2D association signals. We also made use of information extracted from MeSH and OMIM, and performed traits analysis that shows further supporting evidence from diseases comorbidity and symptomatology. The interactome also rediscovered current T2D drug targets and identified potentially druggable ones. We discovered a novel T2D-associated gene near a well-established GWAS locus. This association was further supported by long-range chromatin interaction based on chromosome conformation capture (Hi-C) data. Finally, our T2Di signatures were independently assessed, with high significance, by using a machine learning-based gene prioritization method that was recently published.

Conclusions: Interactome signatures complement the existing GWAS loci, explaining the T2D pathogenesis.

Funding information: This study was supported by Hong Kong Foundation for Research and Development, a Focused Investment Scheme (CUHK) granted to J.C.N.C, a GRF461712 and a CUHK3/CRF/11G granted to T.F.C. and T12-402/13N granted to R.C.W.M.

### 0139

### Strategy for Treatment of Diabetic Polyneuropathy by Cell Transplantation: Possible Utilization of Dental Pulp Stem Cells

Microvascular Complications

K. Naruse[1], M. Hata[2], M. Omi[2], T. Himeno[3], N. Nakamura[1], M. Miyabe[1], M. Kondo[3], H. Kamiya[3], J. Nakamura[3] and T. Matsubara[1]

[1]*Department of Internal Medicine, School of Dentistry, Aichi Gakuin University, Nagoya, Japan,* [2]*Department of Removable Prosthodontics, School of Dentistry, Aichi Gakuin University, Nagoya, Japan,* [3]*Division of Diabetes, Department of Internal Medicine, Aichi Medical University School of Medicine, Nagakute, Japan*

Objective: Progenitor/stem cell transplantation ameliorates diabetic polyneuropathy in experimental animal models. These cell transplantations increased nerve conduction velocity and nerve blood flow as well as intraepidermal nerve fiber density in diabetic animals. Mesenchymal stem cell is an attractive candidate for cell therapy. Especially, dental pulp stem cell (DPSC) which is a sort of mesenchymal stem cells located in the dental pulp is a potential source for cell therapy, since DPSCs can be easily obtained from teeth extracted for orthodontic reasons. In this study, we evaluated the therapeutic potential of DPSC transplantation for diabetic polyneuropathy.

Methods: We isolated DPSCs from the dental pulp of extracted incisors of Sprague-Dawley rats and expanded in the culture system. Cultured DPSCs were transplanted into the unilateral hindlimb skeletal muscles of the STZ-induced diabetic rats and the effects of DPSC transplantation were evaluated 4 weeks after the transplantation. We also examined the effects of cryopreserved DPSCs on diabetic polyneuropathy.

Results: Transplantation of DPSCs significantly improved the impaired sciatic nerve blood flow, sciatic motor/sensory nerve conduction velocity, capillary number-to-muscle fiber ratio and intra-epidermal nerve fiber density in the transplanted side of diabetic rats. Some of the transplanted DPSCs differentiated into vascular endothelial cells, which may increase capillary ratio by the cooperation with the resident vascular endothelial cells. In vitro experiment revealed that DPSC culture supernatant increased neurite outgrowth. We also confirmed that the transplantation of cryopreserved DPSCs ameliorated sciatic nerve blood flow and sciatic nerve conduction velocity as well as freshly-isolated DPSCs.

Conclusion: Since DPSCs can be isolated from teeth extracted in young adults, the obtained DPSCs which can be cryopreserved until needed are not only youthful but also may be stem cells before the onset of diabetes. These findings suggest that DPSCs may be a promising cell source for the treatment of diabetic polyneuropathy.

© 2016 The Authors. Journal of Diabetes Investigation published by Asian Association for the Study of Diabetes (AASD) and John Wiley & Sons Australia, Ltd

### 0140

### Poor glycaemic control is associated with impaired macrophage cholesterol efflux in type 2 diabetes mellitus

Lipid Disorders

J. G. S. Tsun, S. W. M. Shiu, Y. Wong and K. C. B. Tan
*The University of Hong Kong, Hong Kong*

OBJECTIVES: Macrophage foam cell formation plays an important role in the development of atherosclerosis. Accumulation of cholesterol in macrophages is partly influenced by the cells' capability to efflux cholesterol to extracellular cholesterol acceptors. We have evaluated whether there were changes in macrophage cholesterol efflux in patients with poorly controlled type 2 diabetes.

METHOD: Forty type 2 diabetic patients and fifty non-diabetic controls were recruited. Peripheral blood monocytes were isolated and differentiated into macrophages using autologous serum. Cholesterol efflux assay was performed by measuring the percentage of [$^3$H]cholesterol transferred from subject's monocyte-derived macrophages to fixed concentrations of exogenous apolipoprotein (apo) AI, HDL, $HDL_2$ and $HDL_3$ as cholesterol acceptors.

RESULTS: Diabetic patients had elevated triglyceride and lower HDL than controls. As expected, $HbA_{1c}$ was much higher in diabetic patients than control (9.3% ± 0.9 vs 5.4% ± 0.5, $P < 0.01$). In type 2 diabetic patients, macrophage cholesterol efflux to apo AI (18.9% ± 9.5 vs 26.8% ± 10.5, $P < 0.01$) and to HDL (34.2% ± 7.8 vs 43.8% ± 8.6, $P < 0.01$) was significantly impaired compared to controls. Similar reduction in cholesterol efflux to $HDL_2$ and $HDL_3$ was also observed. Macrophage cholesterol efflux to apo AI ($r = -0.39$, $P < 0.01$) and HDL ($r = -0.53$, $P < 0.01$) correlated inversely with $HbA_{1c}$.

CONCLUSIONS: Macrophage cholesterol efflux was significantly impaired in poorly controlled patients with type 2 diabetes mellitus and might contribute to their increased risk of developing cardiovascular diseases. Whether improvement in glycaemic control will restore macrophage cholesterol efflux warrants further investigations.

### 0141

### Sequential changes in clinical findings of type 1 diabetic patients by replacing insulin glargine with insulin degludec

Therapeutics of Diabetes

Y. Kato[1], H. Kamiya[1], M. Kondo[1], T. Himeno[1], E. Naito[1], Y. Sugiura[1], C. Kojima[1], Y. Yamada[1], T. Ando[1], S. Noda[1], H. Nakai[1], E. Nagao[1], T. Hayami[1], E. Asano[1], M. Motegi[1], M. Yoshida[1], Y. Hayashi[1], R. Shimauchi[1], K. Kato[2] and J. Nakamura[1]
[1]*Division of Diabetes, Department of Internal Medicine, Aichi Medical University School of Medicine, Nagakute, Aichi, Japan,* [2]*Laboratory of Medicine, Aichi Gakuin University School of Pharmacy, Chikusa, Nagoya, Japan*

AIMS: It has been known that insulin degludec (Deg) has a prolonged action to lower blood glucose levels but hypoglycemia at night rarely occurs during the treatment with Deg, and that the stationary phase appears 2–3 days after the onset of treatment with Deg. Thus, Deg has different characteristics from those of insulin glargine (Gla). However, the changes in clinical findings of patients by replacing Gla with Deg have been rarely investigated by using continuous glucose monitoring (CGM). This study was aimed at examining how the replacement of Gla with Deg affects the clinical findings of patients with type 1 diabetes mellitus by using CGM.

METHODS: Eleven outpatients with type 1 diabetes mellitus who were given Gla twice a day (male/female: 1/10, age: 57.5 ± 11.6 years, duration of diabetes: 14.2 ± 10.7 years, HbA1c: 7.8 ± 0.5%, BMI: 22.1 ± 2.6 kg/m$^2$) were enrolled in this study. In these patients, the mean of 24 h' blood glucose levels and its standard deviation (SD), frequency of hypoglycemia (<70 mg/dL), mean blood glucose level at night (0–6 a.m.), and frequency of hypoglycemia at night (0–6 a.m.) were sequentially determined after replacing administration of Gla twice a day with Deg once a day by using CGM. Insulin dose administered were sequentially determined after replacing administration of Gla twice a day with Deg once a day.

RESULTS AND DISCUSSION: The mean of 24 h' blood glucose levels seemed to temporarily elevate and thereafter improve. However, the mean of 24 h' blood glucose levels, SD, and frequency of hypoglycemia before and 1–5 days after the replacement of Gla with Deg were not significantly different. There were also no significant differences in the mean blood glucose levels and frequency of hypoglycemia at night between before and after the drug replacement. Furthermore, there was no significant difference in the basal insulin dose between before and after the drug replacement and also the bolus insulin dose did not change by the drug replacement.

CONCLUSION: In patients with type 1 diabetes mellitus, administration of Deg once a day exhibits a very similar hypoglycemic effect to that of Gla twice a day.

### 0142

### Comparison of acquisition data by the medtronic sof-sensor and enlite in continuous glucose monitoring system

Clinical Diabetes

N. Hirai[1], Y. Kato[2], H. Kamiya[2], M. Takagi[1], K. Yamaguchi[1], M. Komiyama[1], M. Kondo[2], T. Himeno[2], Y. Sugiura[2], E. Naito[2], C. Kojima[2], Y. Yamada[2], T. Ando[2], R. Kasagi[2], S. Noda[2], H. Nakai[2], E. Nagao[2], E. Asano[2] and J. Nakamura[2]
[1]*Central Clinical Laboratory, Aichi Medical University Hospital, Nagakute, Aichi, Japan,*
[2]*Division of Diabetes, Department of Internal Medicine, Aichi Medical University School of Medicine, Nagakute, Aichi, Japan*

BACKGROUND: Two types of sensors, the Sof-sensor® and the Enlite, are available to continuous glucose monitoring (CGM) system (CGMS). The purpose of this study was to evaluate and compare the efficacies of both types of sensors for determination of blood glucose levels in CGMS.

METHODS: Six healthy adult males (HbA1c: 5.5 ± 0.2%) and 6 adult males with type 2 diabetes (DM) (HbA1c: 6.8 ± 0.6%) were enrolled in this study. Both types of sensors were inserted subcutaneously into the left and right abdominal regions of the study subjects, respectively, CGMS readings were obtained for 6 days using iPro2 as the recorder. In the period, oral glucose tolerance test (OGTT) was conducted and plasma glucose levels (PG) were determined before, 30, 60, and 120 min after OGTT. CGM values obtained using both types of sensors for the period of 6 days were compared and CGM values obtained at OGTT were compared with PG.

RESULTS: In 6 healthy subjects, all mean CGM values obtained using both types of sensors 30, 60, and 120 min after OGTT had no significant differences from the PG. In subjects with DM, the mean CGM values obtained 60 and 120 min after OGTT using Sof-sensor were significantly lower than the PG ($P < 0.05$), whereas those obtained using Enlite had no significant differences from the PG. Furthermore, in subjects with DM, all mean CGM values during postprandial rise in blood glucose levels (2 h after meal) obtained using the Enlite were significantly higher than those obtained using the Sof-sensor, and the maximal difference between them was 20 mg/dL. Mean CGM values throughout the period of 6 days in healthy subjects obtained using the Sof-sonsor and the Enlite were 98.1 mg/dL and 97.2 mg/dL, respectively, and those in subjects with DM were 148.5 and 147.0 mg/dL, respectively. SDs of CGM values obtained using both types of sensors in healthy subjects were 13.7 mg/dL with the Sof-sensor and 13.9 mg/dL with the Enlite, and those in subjects with DM were 35.5 mg/dL with the Sof-sensor and 36.2 mg/dL with the Enlite.

CONCLUSION: During postprandial rise in blood glucose levels, CGM values obtained using the Enlite approximate more closely to the PG than those obtained using the Sof-sonsor, although CGM values obtained using both types of sensors are very similar at the other usual times. Results demonstrate that the Enlite is superior to the Sof-sonsor in responding rise in blood glucose levels in CGM.

### 0143

### Impact of original diabetes self-management education program including individual self-care plan 'my goals sheet' on glycemic control

Education and Care Delivery

N. Tanaka, K. Watanabe, T. Hyo, H. Kuwata, S. Onizaki, M. Satogami, K. Uose, S. Watanabe, S. Hamasaki, T. Menya, H. Noda, N. Hirose, M. Iwasaki, T. Okamoto, S. Shimizu, Y. Hirawsawa, N. Kitatani, D. Yabe, T. Kurose and Y. Seino
*Kansai Electric Power Hospital, Osaka, Japan*

OBJECTIVES: In-patients with diabetes mellitus are referred to Diabetes-educational program (DEP) during hospitalization, and DEP is usually provided by

© 2016 The Authors. Journal of Diabetes Investigation published by
Asian Association for the Study of Diabetes (AASD) and John Wiley & Sons Australia, Ltd diabetologists and health care professionals in Japan. In our hospital, we developed a new DEP including lectures, discussions with patients, diet and exercise instructions, Diabetes Conversation Map™ provided by diabetes specialists, nurses, dietitians, pharmacists, physiotherapists, laboratory technicians and so on. We also developed 'My Goals Sheet' (MG Sheet), which is a self-evaluation tool for declaring a patient's goals in his/her life for shorter, intermediate and longer period and self-directed goal setting in healthy eating, physical activity, medication and reducing risks addressed through DEP. The aim of this study is to investigate the efficacy and validity of the new DEP in glycemic control.

METHODS: Retrospective, observational study was conducted with 51 patients who participated in the new DEP and completed their own MG Sheet (Group N, male / female =31 / 20, mean ± SD age: 60.5 ± 13.9 years; BMI: 26.8 ± 4.3 kg/m$^2$; HbA1c: 9.2 ± 1.9%). As a control group, patients who participated in previously adopted DEP (mainly video lectures) (Group C, $n$ = 51) were included. Their age, sex, anthropometric factors and glycemic control were matched with Group N. $HbA_{1c}$ levels were evaluated until 9 months after the admission with DEP.

RESULTS: $HbA_{1c}$ levels after both of DEP decreased during follow-up period: Group N vs Group C; 9.2% vs 9.2% at baseline; 7.9% vs 8.9% in 1 month; 7.4% vs 7.7% in 3 months; 7.5% vs 7.8% in 6 months; 7.4% vs 8.0% in 9 months. $HbA_{1c}$ in Group N sustained significantly lower level after 9 months ($P$ = 0.0289) compared to Group C. Patients who could set assessable and achievable goals in 6 or 7 items out of 7 questionnaires in MG Sheet in Group N reached significantly greater reductions in changes of $HbA_{1c}$ compared with Group C (−2.2% vs −1.1% in 9 months, $P$ = 0.0373), although those who could not set proper goals in Group N reached no significant reductions (−1.3 vs −1.1 in 9 months).

CONCLUSIONS: New DEP combined with MG Sheet might be effective in maintaining lower $HbA_{1c}$ levels lower if the assessable and achievable goals are determined appropriately by the patients.

### 0144

### A case of mitochondrial diabetes with tRNA $^{Leu(UUR)}$ mutation at position 3271 and autoimmune pancreatitis after SGLT2 inhibitor treatment

Y. Suzuki
*HDC Atlas Clinic, Tokyo, Japan*

A male patient who had mitochondrial diabetes associated with tRNA $^{Leu(UUR)}$ mutation at position 3271 was first reported in 1996 and has been followed for over 20 years. At the age of 46, his glycemic control worsened together with marked elevation of glutamic acid decarboxylase antibody (GADA). After GADA spontaneously declined again, good glycemic control was maintained with oral hypoglycemic agents.

In early 2014, he started treatment with a sodium-glucose transporter 2 (SGLT2) inhibitor, showing rapid weight loss in a 3-month period from 59.5 kg (BMI of 19.7 kg/m$^2$) to 56 kg (BMI of 18.5) and achieving excellent glycemic control with a hemoglobin $A_{1c}$ ($HbA_{1c}$) of 6.2% (44 mmol/mol).

In March 2015 (aged 59 years), routine blood tests revealed elevation of pancreatic parameters, with a serum amylase of 3.32 μkat/L (normal: <2.09), CA19–9 of 55.3 μ/mL (normal: <37), elastase-I of 330 ng/dL (normal: <300), and immunoglobulin G4 of 1.15 g/L (normal: <1.05). Imaging did not detect a pancreatic mass or any signs of pancreatitis. Along with a gradual rise of GADA to 1.7 U/mL (normal: <1.5), $HbA_{1c}$ increased to 6.5% (48 mmol/mol).

Because ketone bodies were elevated to 484 μmol/L (normal: 26–122), we advised him to increase his intake of carbohydrate and protein to stop catabolism. Thereafter, ketone bodies decreased and the pancreatic parameters returned to normal. In addition, GADA declined to 0.9 U/mL and $HbA_{1c}$ returned to 6.2% (44 mmol/mol).

Electron transport chain dysfunction and excessive generation of reactive oxygen species are hallmarks of mitochondrial injury in the diabetic pancreas and other organs. It is possible that mitochondrial activity exceeds energy requirements in patients receiving SGLT2 inhibitors, increasing the release of oxidants and leading to pancreas damage. Electron transport chain dysfunction and oxidative stress could have been augmented in our patient by mitochondrial membrane defects due to the 3271 mutation. Therefore, we investigated whether compensatory overeating could attenuate our patient's autoimmune phenomena. Interestingly, compensatory overeating reduced euglycemic ketosis and prevented exacerbation of the autoimmune changes, along with a return to excellent glycemic control.

In conclusion, physicians should take care if they prescribe SGLT2 inhibitors for patients with mitochondrial diabetes. When patients with type 2 diabetes treated by SGLT2 inhibitors develop euglycemic ketosis and unusual adverse reactions, mitochondrial disorders might have a potential role. This is the first reported evidence that abnormal fluctuation of pancreatic markers and immunoglobulin G4 can be associated with mitochondrial DNA abnormality.

### 0145

### Family history of diabetes increases the risk of metabolic disorders but does not affect risk reducing behavior in young Korean adults: from the Korean national health and nutrition surveys 2010

Epidemiology

J. H. Moon[1], E. Roh[1], K. M. Kim[1,2], J. H. Moon[1,2], S. Lim[1,2], H. C. Jang[1,2] and S. H. Choi[1,2]
[1]*Seoul National University, Seoul, Republic of Korea,* [2]*Seoul National University Bundang Hospital, Seongnam, Republic of Korea*

OBJECTIVE: Genetic predisposition is one of the key risk factors of type 2 diabetes mellitus (T2DM). We investigated the impact of a family history of diabetes on the prevalence of T2DM and metabolic syndrome and metabolic risk reducing behavior in Korean young adults.

METHOD: We used the Korea National Health and Nutrition Surveys 2010 for analysis. Subjects aged 25 to 44 years were included and family history of diabetes in first-degree relatives was obtained from self-reported questionnaire. The prevalence of T2DM and metabolic syndrome were estimated using the American Diabetes Association guideline and the revised National Cholesterol Education Program criteria. Exercise was defined as moderate or vigorous intensity physical activities and dietary data were collected by 24-h recall interview. A subgroup of their parents ($n$ = 578, 28.1%) were included for the correlation analysis for metabolic parameters between their offspring.

RESULTS: Among total 2,059 participants, subjects with a family history of diabetes ($n$ = 489, 23.7%) had significantly higher prevalence of impaired fasting glucose (14.3% vs. 11.7%) and T2DM (6.7% vs. 1.8%) compared with those without a family history ($n$ = 1,570). Metabolic syndrome (21.3% vs. 12.1%) and each component (except HDL cholesterol) were more prevalent in subjects with a family history. Among subjects without diabetes ($n$ = 1,998), fasting glucose (91.7 vs. 89.9 mg/dL) and triglyceride (99.0 vs. 89.0 mg/dL) concentration were significantly higher in subjects with family history. Participants' BMI, waist circumference, and triglyceride concentration were significantly correlated with that of both parents', whereas fasting plasma glucose and HOMA-β were only correlated with that of mother. The risk reducing behavior including exercise (18.2% vs. 19.7%) and calorie intake (2174.8 vs. 2149.1 kcal/day) did not substantially differ by family history of diabetes. Among subjects who developed T2DM, recognition (63.6% vs. 57.1%) and treatment rate (42.4% vs. 32.1%) were slightly higher in subjects with family history, but their fasting glucose was higher (170.9 vs. 140.3 mg/dL, $P$ = 0.050) suggesting poorly controlled diabetes in this group.

CONCLUSION: In subjects with a family history of diabetes had higher prevalence of T2DM and metabolic syndrome and worse cardiovascular risk factors. Our findings suggest that family history of diabetes can be a valuable tool to distinguish young adults at increased risk of future metabolic disorders.

### 0146

### Cardiovascular Risk Management of Diabetes Patient in South Korea

Macrovascular Complications

D.-M. Kim and C. Kim
*Hallym University College of Medicine, Seoul, Republic of Korea*

Current guidelines on cardiovascular (CV) disease prevention recommend targeted management after assessment of CV risks using many of available method

© 2016 The Authors. Journal of Diabetes Investigation published by Asian Association for the Study of Diabetes (AASD) and John Wiley & Sons Australia, Ltd even if the patient is asymptomatic. This study was performed to explore how CV high risk is differently detected between 2 distinct methods: non-invasive test (NIT) and risk calculation and how the awareness of CV high risk impacts physician and patient behavior for risk management in diabetes patient.

A prospective, observational study was carried out in 22 hospitals in Korea. 622 type 2 diabetes patients aged ≥40 years were assessed by Carotid Ultrasound (CUS) and United Kingdom Prospective Diabetes Study (UKPDS) risk engine. CV high risk from CUS was defined as carotid intima-media thickness ≥ 1 mm or plaque presence. Before and 6 months after the test, patients completed a questionnaire on health-related behaviors and physicians collected data on treatment patterns via chart review.

Among 622 (mean age: 60.02 ± 9.50 years), 271 (43.5%) and 66 (10.6%) patients were stratified as CV high risk from CUS and UKPDS, respectively. Approximately 40% of patients at moderate and low risk from UKPDS were determined as high risk from CUS. The awareness of high risk from CUS altered physician's treatment patterns ($P = 0.021$) for managing major CV risk factors: blood pressure (BP) and lipid. Along with CUS, risk levels by UKPDS also impacted physician's behavior: more changes of treatment pattern in high than in low risk level for BP (12.0 vs 15.6%) and lipid (13.8 vs 21.6%). Patients noted increased health-related behaviors: smoking cessation and dietary changes ($P < 0.005$, respectively) in 6 month follow up than before CUS and bigger in high risk.

This study identified NIT could detect more CV high risk patients than risk calculation. However, awareness of CV high risk itself has a positive impact on physician and patient behavior, regardless of assessment methods. Therefore, assessing CV risks using varied methods with the patient could be better for risk management in diabetes patient.

### 0147

### Annual progression rates of chronic kidney disease stages 3A, 3B and 4 to end stage renal disease in diabetes patients in Singapore

Microvascular Complications

Y. Ang and B. H. Heng
*National Healthcare Group, Singapore, Singapore*

Background: Singapore has the 4th highest incidence (285 cases per million) of end stage renal disease (ESRD) in the world after Jalisco, Taiwan and United States of America. Singapore also has the highest rate of patients with new ESRD due to diabetes mellitus (66 cases per million).

Objectives: The objectives of the study is to determine the annual progression rates of chronic kidney disease by the different stages to ESRD in diabetes patients in Singapore. Knowing the rates would help us in evaluating the effectiveness of our interventions to retard the alarming progression to end stage renal disease.

Method: This is a retrospective cohort study of diabetes patients with stage 3A, 3B and 4 chronic kidney disease (estimated glomerular filtration rate [GFR] less than 60 mL/min per 1.73 $m^2$ on 2 occasions taken 90 days apart) who visited the 9 government-funded primary care clinics (National Healthcare Group polyclinics) from 1 January 2007 to 31 December 2007. The patients were followed up till 30 December 2014 and the event of interest was progression to ESRD (estimated GFR less than 15 mL/min per 1.73 $m^2$ on 2 occasions taken 90 days apart). Serum creatinine was measured using an Isotope Dilution Mass Spectrometry traceable standard and estimated GFR was estimated using the abbreviated Modification of Diet in Renal Disease equation and standardised creatinine. The annual progression rates were determined using time to event analysis. All analyses were conducted using STATA (StataCorp, College Station, TX, USA) statistical software, version 12.0. The study was approved by the National Healthcare Group Domain-specific Ethics Board E which is an independent committee constituting of medical, scientific and nonscientific members.

Results: There were 4,935 stage 3A patients, 4,034 stage 3B patients and 1,212 stage 4 patients. Their mean ages were 70.7 (standard deviation [SD]: 9.8), 72.0 (SD: 10.0) and 72.4 (SD: 10.3) years old respectively. The mean follow up periods were 3.5 (SD: 2.3), 3.3 (SD: 2.2) and 2.4 (SD: 1.9) years respectively. The percentage of patients who progressed at the end of the study period were 2.8, 9.3 and 36.8% respectively. The annual progression rates to ESRD were 0.8 (95% CI 0.6–0.9), 2.8 (95% CI 2.5–3.1) and 15.2 (95% CI 13.9–16.7)% respectively.

Conclusions: We have estimated the annual progression rates to ESRD in diabetes patients and found that the rates are higher with higher stages of disease. Interventions to retard progression can use these rates to evaluate their effectiveness.

### 0148

### Targeted metabolomics study reveals incomplete fatty acids oxidation and abnormal amino acids catabolism in patients with diabetic kidney disease – a metabolic signature suggestive of mitochondria impairment and metabolic inflexibility

Microvascular Complications

J. J. Liu[1], S. C. Lim[1], J. P. Kovalik[2], S. Ghosh[2], E. S. Tai[3], J. H. Ching[2], C. N. Ong[3], S. K. M. Low[1], T. Subramaniam[1] and C. F. Sum[1]
[1]*Khoo Teck Puat Hospital, Singapore,* [2]*Duke-NUS Graduate Medical School, Singapore, Singapore,* [3]*National University of Singapore, Singapore, Singapore*

Introduction and aims: The pathophysiological basis for development and progression of diabetic kidney disease (DKD) in patients with type 2 diabetes (T2DM) are still incompletely understood. Metabolomics offers a high resolution evaluation of small molecules and provide insights for disease mechanisms. We aim to study metabolic abnormalities associated with DKD in patients with T2DM by targeted metabolomics.

Subjects and Methodology: In this case-control study, 149 subjects with T2DM and overt DKD (urinary albumin-to-creatinine ratio, ACR >= 300 mg/g, regardless of eGFR level) and 149 T2DM subjects without manifestation of DKD (urinary ACR < 30 mg/g and eGFR >= 60 mL/min/1.73 $m^2$) were matched by race, gender, age and diabetes duration. HPLC-MS/MS targeted metabolomics was applied to quantify 7 organic acids in tricarboxylic acid cycle (TCA), 45 sub-species of acylcarnitines and 16 amino acids in plasma.

Results: Age of the subjects was 57+/−10 years, 62.5% were male, 71.2% were Chinese and diabetes duration was 10.7+/−7.0 years. Urinary ACR was 861 (IQR 462–2098) mg/g and eGFR was 62+/−32 mL/min in cases whist ACR was 9 (IQR 6–18) mg/g and eGFR was 94+/−21 mL/min in controls ($P < 0.0001$ and $P < 0.001$, respectively). Subjects with overt DKD had higher HbA1c (8.8 +/−2.2 vs 8.1 +/−1.8%, $P = 0.004$) and higher SBP (144+/−21 vs 131+/17 mmHg, $P < 0.0001$). There were no differences in body mass index (BMI) between two groups.

Targeted metabolomics assay showed that medium-chain acylcarnitines (C12-OH/C10-DC, C8:1-OH/C6:1-DC, C8-OH/C6-DC, C8:1-DC, C8-DC and C6, *q-values* range from 2.87e-6 to 9.87e-13) and short-chain acylcarnitines (C2, C3-DC, C4, C4-DC, C4-OH, C5, C5:1, C5-DC, *q-values* range from 7.79e-5 to 2.03e-22) were significantly higher in subjects with DKD as compared with controls. In addition, plasma levels of Trp, Ala, Val, Ile/Leu were lower (*q-values* from <0.0005 to 1.03e-6) and the level of TCA intermediate citrate was significantly higher (*q-value* 1.01e-7) in patients with DKD. There were no significant differences in long-chain aclycarintine levels (C16 to C-22) between cases and controls.

Interpretation/Conclusion: Elevated plasma medium-chain acylcarnitines, acetylcarnitine (C2) and citrate levels suggest incomplete fatty acid oxidation (FAO) in patients with DKD due to elevated influx of FAO and/or reduced TCA activities. Higher short-chain (C3–C5) acylcarnitines accompanied by lower Trp, Ala, Val, Ile/Leu levels indicate enhanced amino acids (especially branched-chain amino acids) catabolism. The mismatch between FAO, amino acids catabolism and TCA activity points to dysregulation in substrate usage for cellular energy production in patients with DKD which may result from impaired mitochondria function.

### 0150

### Effects of switching conventional continuous subcutaneous insulin infusion therapy to sensor augmented pump therapy on diurnal glycemic profiles in Japanese type 1 diabetic patients

Clinical Diabetes

A. Matsuoka, Y. Hirota, T. Hamaguchi, S. Urai, N. Suematsu, A. Sou, T. Nakamura, H. Komada, Y. Okada, K. Sakaguchi and W. Ogawa
*Kobe University Graduate School of Medicine, Department of Internal Medicine, Division of Diabetes and Endocrinology, Kobe/Hyogo, Japan*

Objectives: We investigated effects of switching conventional continuous subcutaneous insulin infusion (CSII) therapy to sensor augmented pump (SAP) therapy on diurnal glycemic profiles in Japanese type 1 diabetic patients.

© 2016 The Authors. Journal of Diabetes Investigation published by Asian Association for the Study of Diabetes (AASD) and John Wiley & Sons Australia, Ltd

METHODS: Treatment with conventional CSII was changed to that with SAP (MiniMed 620G, Medtronic) in 18 Japanese type 1 diabetic outpatients (gender, male 2/ female 16; age, 41.2 ±16.9 years; BMI, 22.7 ±3.7 kg/m$^2$; duration of diabetes, 11.6 ±8.3 years; duration of pump therapy, 3.7 ±1.7 years). Within 1 month before the change of the treatment, glycemic profiles of the patients were investigated with the use of a retrospective continuous glucose monitoring (CGM) system. Three months after the change of the treatment, time of hypoglycemic range (<70 mg/dL), time of hyperglycemic range (>180 mg/dL), AUC of hyperglycemic range, AUC of hyperglycemic range, mean glucose level, standard deviation (SD), and mean amplitude of glycemic excursions (MAGE) were calculated with CGM data equipped with the SAP, and were compared to those obtained before the change of the treatment. HbA1c, GA and insulin dosage were also investigated before and 3 months after the change of the treatment.

RESULTS: The average time of hypoglycemic range decreased significantly (159.5 to 76.0 min/day, $P = 0.01$) and the average AUC of hypoglycemic range tended to decrease (1.2–0.8 mg/dL*hr, $P = 0.09$) after the change of the treatment. No differences in the average time of hyperglycemic range, the average AUC of hyperglycemic range, the mean glucose level, SD, and the MAGE were observed after the change of the treatment. The levels of HbA1c (7.8–7.7%, $P = 0.43$) and of GA (22.1–23.2%, $P = 0.17$) were unaltered after treatment change, as was in insulin dosage. The median sensor use over 3 months was 72.5%.

In patients with HbA1c levels <8.0%, the level of HbA1c was unaltered (7.1–7.3%, $P = 0.37$) whereas the average time of hypoglycemic range decreased significantly (170.5–101.0 min/day, $P = 0.01$). In contrast, in patients with HbA1c levels ≧8.0%, the level of HbA1c decreased significantly (8.6–8.1%, $P = 0.03$) and the average time of hypoglycemic range tended to decrease(145.7–31.5 min/day, $P = 0.06$).

CONCLUSION: The switching conventional CSII therapy to SAP therapy resulted in the tendency to reduce hypoglycemia. In patients with HbA1c levels <8.0%, the average time of hypoglycemia decreased without a significant decrease in the HbA1c level, and in patients with HbA1c levels ≧8.0%, the HbA1c level decreased with tendency to decrease in the average time of hypoglycemia.

### 0151

### Clinical characteristics and outcomes of urinary tract infections in diabetic and non-diabetic patients

Clinical Diabetes

A. Kawaguchi[1], A. Sako[1], T. Kondo[2], Y. Masui[1] and H. Yanai[1]

[1]*Department of Internal Medicine, Kohnodai Hospital, National Center for Global Health and Medicine, Ichikawa, Chiba prefecture, Japan,* [2]*Department of Psychiatry, Kohnodai Hospital, National Center for Global and Medicine, Ichikawa, Chiba prefecture, Japan*

OBJECTIVES: Diabetic patients have higher incidence of urinary tract infections (UTIs) and increased risk of complications and mortality due to UTIs. The aim of this study was find the differences in the clinical characteristics and outcomes of UTIs between diabetic and non-diabetic patients.

METHODS: We selected the patients who admitted to the department of internal medicine because of UTIs between January 2012 and October 2014. We excluded the patients who had hospital- acquired UTIs and the patients who had neither pyuria nor bacteriuria. We retrospectively collected the data including demographic data, biochemistry data, microbiological data and clinical outcomes. We compare the characteristics and outcomes between diabetic patients and non-diabetic patients by using t-test and chi-square test as appropriate. Institutional review board approved this study.

RESULTS: Among 3,139 patients who admitted to the department of internal medicine during study period, total of 99 cases were eligible and 36 were type 2 diabetic patients. The mean plasma glucose level was 222 ± 98 mg/dL and the mean HbA1c was 7.5 ± 1.6%. No patients used Sodium glucose trasporter-2 inhibitor. Thirty four cases were male, and mean age was 73 years old. Diabetic patients had significantly higher counts of blood leukocytes (13,300 ± 5,900 /mm$^3$ vs 11,200 ± 4,400 /mm$^3$, $P = 0.045$), and marginally higher serum C-reactive protein(CRP) (13.4 ± 9.5 mg/dL vs 10.8 ± 7.8 mg/dL, $P = 0.148$). The most common organism isolated from urine in both diabetic and non-diabetic patients was Escherichia coli (50.0% vs 51.9%, $P = 0.87$). There was no difference in the isolation rates of extended-spectrum β-lactamase producing Escherichia coli (5.9% vs 14.8%, $P = 0.31$). In terms of clinical outcomes, rates of bacteremia were similar in both groups (44.1% vs 34.5%, $P = 0.36$). The duration of hospitalization tended to be longer (26.1 day vs 20.7 day, $P = 0.20$) in diabetic patients. Only one death was observed in non-diabetic patients.

CONCLUSIONS: Although higher blood leukocyte counts and serum CRP suggested more sever conditions of UTIs in diabetic patients as compared with non-diabetic patients,there was no significant difference of the duration of hospitalization and mortality between diabetic and non-diabetic patients.

### 0153

### Damage associated molecular pattern (DAMP) proteins S100A8 and S100A9 are involved in exacerbating doxorubicin-induced cardiomyopathy in diabetic heart

Clinical Diabetes

X. Pei, B. Y. Yung, S. P. Yip, L. W. Chan, C. S. Wong, M. T. Ying and P. M. Siu

*The Hong Kong Polytechnic University, Kowloon, Hong Kong*

OBJECTIVES: Cardiomyopathy is a well-known clinical problem in the heart that occurs in type 2 diabetic mellitus and during doxorubicin chemotherapy. The number of diabetic cancer patients is indeed increasing but surprisingly the cardiac damaging effects of the anti-cancer drug doxorubicin on a diabetic heart have not been well-examined. As the signaling mechanisms of the doxorubicin-induced cardiomyopathy in a type 2 diabetic heart are mostly unknown, this study examined the molecular signaling that is responsible for doxorubicin-induced cardiotoxicity in type 2 diabetic heart.

MATERIALS AND METHODS: Fourteen- to eighteen-week-old db/db diabetic and db/+ non-diabetic mice were randomly assigned to the following groups: db/+-Saline ($n = 8$), db/+-DOX (DOX, doxorubicin; $n = 8$), db/db-Saline ($n = 8$), and db/db-DOX ($n = 8$). Mice assigned to DOX group were exposed to an intraperitoneal (i.p.) injection of DOX at a dose of 15 mg/kg to induce cardiomyopathy. Mice in sham groups (Saline) were i.p. injected with the same volume of saline instead of DOX. Mice were euthanized by overdose of ketamine and xylazineon 5 days or 7 days after the experiment. Ventricular fractional shortening was examined as an indicator of cardiac function by transthoracic echocardiography. Microarray analysis was adopted to examine the changes of the whole transcriptional profile in response to the administration of doxorubicin in diabetic heart.

RESULTS: The presence of diabetic cardiomyopathy was evident by the reduction in fractional shortening shown in db/db mice. Doxorubicin induced cardiac dysfunction in db/+ non-diabetic mice on day 5 after the administration of doxorubicin as illustrated by a significant reduction from 69.3% to 52.1% in the cardiac fractional shortening. Doxorubicin significantly induced a further impairment of cardiac contractile function in db/db diabetic mice when compared to db/+ non-diabetic mice on day 7 but not day 5 after the doxorubicin administration. The microarray analysis identified a panel of regulatory genes associated with cardiac remodeling, inflammatory response, oxidative stress, and DNA/RNA stability/repair in the doxorubicin-induced cardiac injury in diabetic heart. The microarray results were confirmed by the analysis of transcript expression of selected genes by real time PCR. Notably, two damage-associated molecular pattern (DAMP) genes, namely S100A8 and S100A9, were evidently shown to have a unique specific expression pattern related to the doxorubicin-induced cardiomyopathy in diabetic heart.

CONCLUSIONS: These findings provide important pre-clinical information for future research to develop effective treatment or intervention in solving the clinical problem of the doxorubicin-induced cardiac toxicity in diabetic cancer patients.

### 0154

### Effects of resveratrol-mediated SIRT1 activation on insulin, apoptotic and catabolic singling in aged skeletal muscle exposed to doxorubicin

Insulin Signaling/Action

B. T. Tam[1], T. K. Sin[1], A. P. Yu[1], S. P. Yip[1], B. Y. Yung[1], L. W. Chan[1], C. S. Wong[1], J. A. Rudd[2] and P. M. Siu[1]

[1]*Department of Health Technology and Informatics, Faculty of Health and Social Sciences The Hong Kong Polytechnic University, Hung Hom, Kowloon, Hong Kong, China,* [2]*School of Biomedical Science, Faculty of Medicine The Chinese University of Hong Kong, Sha Tin, New Territories, Hong Kong, China*

OBJECTIVES: Doxorubicin is a commonly used anti-cancer medication treating malignancies. However, the effects of doxorubicin on insulin signaling in skeletal

© 2016 The Authors. Journal of Diabetes Investigation published by Asian Association for the Study of Diabetes (AASD) and John Wiley & Sons Australia, Ltd muscle remain unknown. In the current study, we studied the aging effects on insulin signaling in skeletal muscle and tested the hypothesis that treatment of doxorubicin results in disruption of insulin signaling in aged skeletal muscle. We further examined whether activation of SIRT1, a NAD-dependent protein deacetylase, by resveratrol alters insulin signaling in doxorubicin-treated skeletal muscle.

METHODS: Two- and 10-month old Senescence Accelerated Mice Prone 8 (SAMP8) were examined. Mice were randomly assigned to the following 5 groups receiving different treatments – 1) Saline (SC); 2) Doxorubicin (DV); 3) doxorubicin + resveratrol (DR); 4) doxorubicin + resveratrol + sirtinol (DRS); and 5) doxorubicin + resveratrol + EX527 (DRE).

RESULTS: Membranous Glut4 protein, activity of PDH and phosphorylation of Akt were decreased in aged muscles compared to young muscles. Phosphorylation of IRS-1 and PDK4 protein content were increased in aged muscles compared to young muscles. Doxorubicin decreased the protein expression of PDK1, phosphorylation of Akt and SIRT1 deacetylase activity and increased the protein expression of PDK4 in young muscle. However, it only decreased the protein expression of PDK1 and SIRT1 deacetylase activity in aged muscle. Intriguingly, resveratrol normalized the doxorubicin-induced alterations in insulin signaling such as decreasing PDK4 protein expression and increasing phosphorylation of Akt and PDK1 protein expression in young muscle.

CONCLUSIONS: Aging impaired the insulin signaling in skeletal muscle. To our surprise, doxorubicin did not further worsen the insulin signaling in aged muscle. Markers of insulin signaling were affected by doxorubicin and resveratrol in young muscle but not in aged muscle. Resveratrol restored the insulin signaling in young skeletal muscle treated with doxorubicin by a SIRT1-dependent signaling manner.

### 0155

### Early phase glucagon secretion associated with gip reflects preferentially glucose disposal after oral glucose loading

Integrated Physiology

A. Hamasaki[1,2], A. Muraoka[1,3], N. Harada[1], S. Yamane[1], E. Joo[1], K. Suzuki[1], K. Shibue[1], T. Harada[1], K. Iwasaki[1] and N. Inagaki[1]

*[1]Department of Diabetes, Endocrinology and Nutrition, Graduate school of Medicine, Kyoto University, Kyoto, Japan, [2]Tazuke Kofukai Medical Research Institute Kitano Hospital, Osaka, Japan, [3]Department of Diabetes and Endocrinology, Shiga Medical Center for Adults, Moriyama, Japan*

BACKGROUND: Plasma glucagon concentrations decrease after oral glucose ingestion in normal glucose tolerance (NGT), while, in type 2 diabetes (T2DM) those fail to decrease appropriately, and furthermore increase paradoxically. We previously showed that gut-derived factor is involved in the mechanisms of paradoxical increase of glucagon concentration after oral glucose loading in T2DM and that alpha-cell response to GIP might lead to inappropriate glucagon secretion. Together with the impaired insulin secretion, inappropriate glucagon response to oral glucose loading (associated with secretory response of gut-derived factors) is thought to be important for glucose disposal. The relationships between them, however, remain to be elucidated. In this study, we examined the relationship between insulin, glucagon, incretin secretion during oral and intravenous glucose loading and calculated the gastrointestinally mediated glucose disposal (GIGD) for evaluation of ability for glucose disposal after oral glucose loading, aiming to investigate relationship glucose disposal function and islet and gastrointestinal hormones.

METHOD: Isoglycemic 3-h oral (75 g oral glucose tolerance test (OGTT)) and intravenous glucose administration (ivGTT) were performed in eight Japanese T2DM and eight NGT subjects. Isoglycemic intravenous glucose infusion was performed using artificial pancreas system STG-22 (Nikkiso, Tokyo, Japan). Plasma and serum concentrations of glucose, insulin, CPR, glucagon, and incretin hormones were measured. GIGD was calculated by using the amount of glucose required by intravenous infusion to reproduce the glucose excursions after the oral glucose loading. Incretin effect was also calculated by the difference in integrated incremental β-cell secretory responses during OGTT and ivGTT.

RESULT: In OGTT, early phase insulin secretion is significantly impaired and early phase glucagon response is significantly increased in T2DM. Not insulin concentration but early phase glucagon response is significantly correlated to GIGD ($P < 0.02$). Incretin effect calculated by β-cell secretory responses in T2DM is not significant different compared to that in NGT, and there is no significant relationship between GIGD and incretin effect (by β-cell secretory responses).

CONCLUSION: Our results indicate that gut-derived factors and their modification of glucagon secretory response is involved in the glucose disposal after oral glucose loading and that early phase glucagon concentration is preferentially related to ability for glucose disposal.

### 0157

### Association of Endothelin-1 (ET-1) and matrix metallopeptidase-9 (MMP-9) with metabolic syndrome in middle-aged and senior Chinese adults

Prediabetes and Metabolic Syndrome

P. H. A. Yu, W. Y. Yau, K. S. Chan, S. Yu, T. Chung and M. F. P. Siu

*The Hong Kong Polytechnic University, Hong Kong*

OBJECTIVES: Metabolic syndrome (MetS) contains a clustering of cardiovascular risk factors including high blood pressure, central obesity, insulin resistance, central obesity and dyslipidemia. People diagnosed with MetS are more susceptible to cardiovascular disease and diabetes mellitus. It has been reported that the abundances of a vasoconstrictor, endothelin-1 (ET-1), and a matrixin, matrix metallopeptidase-9 (MMP-9), were significantly higher in patients with diabetes mellitus and cardiovascular disease. This study aimed to examine the association of MetS with ET-1 and MMP-9 in middle-aged and senior adults.

METHODS: Adults with age ranged from 50 to 92 were voluntarily recruited in this study. All subjects were screened for MetS according to the diagnostic guideline of the United States National Cholesterol Education Program (NCEP) Expert Panel Adult Treatment Panel (ATP) III criteria. The serum levels of ET-1 and MMP-9 were assayed by commercially available chemiluminescent enzyme-linked immunoassay kits.

RESULTS: There are 149 adults ($n = 75$ for Non-MetS Control group and $n = 74$ for MetS group) recruited. The serum level of ET-1 is significantly higher in the MetS-positive subjects compared to the MetS-negative subjects ($P = 0.03$) and is significantly higher in subjects with high blood pressure ($P = 0.001$), high fasting blood glucose ($P = 0.006$) and central obesity ($P = 0.006$). The serum concentration of MMP-9 is significantly higher in subjects positively diagnosed with MetS ($P = 0.001$) compared to the non-MetS Control. The MMP-9 levels of subjects with high blood pressure ($P = 0.001$), high fasting blood glucose ($P = 0.012$), low high-density lipoprotein (HDL) ($P = 0.001$), high triglyceride ($P = 0.018$) and central obesity ($P = 0.001$) are significantly higher compared to subjects negatively diagnosed with those parameters.

The correlation analysis has revealed that the concentration of ET-1 is positively correlated to systolic blood pressure ($P = 0.001$, R=0.423), waist circumference ($P = 0.001$, R=0.274) and fasting blood glucose ($P = 0.002$, R=0.253), while negatively correlated with HDL ($P = 0.001$, R=0.394). The concentration of MMP-9 is positively correlated with systolic blood pressure ($P = 0.001$, R=0.423), waist circumference ($P = 0.001$, R=0.305), fasting blood glucose ($P = 0.001$, R=0.321) and negatively correlated with HDL ($P = 0.001$, R=0.390).

CONCLUSION: The serum levels of ET-1 and MMP-9 are significantly higher in subjects with MetS compared to those without MetS. The circulating levels of ET-1 are also higher in subjects with high blood pressure, high fasting blood glucose and central obesity while the level of MMP-9 is higher in subjects with either one of the MetS parameters. Both circulating levels of ET-1 and MMP-9 are correlated with systolic blood pressure, waist circumference, fasting blood glucose and HDL.

### 0159

### A 24-week structured multidisciplinary weight reduction program in overweight type 2 diabetic patients in a hospital outpatient setting

Clinical Diabetes

T. W. Wong[1], K. M. Ching[1], W. S. Chan[2], H. Lee[1], S. C. Wong[1], A. S[1] and J. Y. Y. Leung[1]

*[1]Ruttonjee Hospital, Hong Kong, [2]Pamela Youde Nethersole Hospital, Hong Kong*

BACKGROUND: Weight reduction is an essential and challenging management for overweight type 2 diabetic (T2DM) patients.

© 2016 The Authors. Journal of Diabetes Investigation published by
Asian Association for the Study of Diabetes (AASD) and John Wiley & Sons Australia, Ltd

OBJECTIVES: To examine the effects of two different meal plans on weight loss and glycemic control in T2DM subjects.

METHOD: It is a randomized, non-blinded, single center trial of meal replacement group (MR) vs dietary prescription group (DP) over 24 weeks. Subjects in MR were to replace at least one meal with less than 300 kcal. While subjects in DP were to undergo self-reduction in caloric intake based on dietary principle.

Both groups received 10 individual face-to-face sessions with endocrinologist, dietitian, or diabetic nurse for a total of 300 min.

RESULTS: From February 2014 to December 2014, 37 subjects (54% male vs. 46% female), with mean age of 54 years (±6.85 years), mean BMI of 31.68 kg/m$^2$ (±4.24 kg/m$^2$), and mean DM duration of 12 years (± 6.09 years) were recruited.

By the end of study, 46% had achieved both reduction in body weight and HbA1c, 27% had weight loss only, while 19% had HbA1c reduction only. 8% of participants showed improvement in neither body weight nor HbA1c.

Mean weight loss was 1.81 kg (±2.85 kg) and 1.89 kg (± 2.37 kg) at 12 and 24 weeks respectively in MR ($P$ = 0.015). Mean weight loss was 2.00 kg (±1.59 kg) and 1.99 kg (±2.25 kg) at 12 and 24 weeks respectively in DP ($P$ = 0.001). There was no significant difference in weight loss between two groups ($P$ = 0.83).

HbA1c decreased from 7.66% (±1.13%) to 7.01% (±0.92%) to 7.00% (±0.92%) at 0, 12 and 24 weeks respectively in MR; and from 7.47% (±0.82%) to 7.02% (±0.76%) to 7.17% (±0.68%) respectively in DP. A greater mean HbA1c reduction of 0.53% (±0.81%, $P$ = 0.013) was achieved in MR, in comparison with 0.31% (±0.64%, $P$ = 0.053) in DR, though with no significant difference ($P$ = 0.35).

Waist circumference reduction was greater in MR than in DP at 24 weeks, −2.39 cm vs. −1.56 cm respectively ($P$ = 0.55). Likewise, visceral fat area reduction was greater in MR than in DP at 24 weeks, −4.82 cm$^2$ vs. −2.28 cm$^2$ respectively ($P$ = 0.46).

Among the insulin users ($n$ = 15), insulin requirement was reduced from average daily dose of 61 to 51 units in MR ($n$ = 8), and from 81 to 70 units in DP ($n$ = 7).

CONCLUSIONS: This structural 24-week multidisciplinary weight reduction program tailored for T2DM patients demonstrated that meal replacement or diet prescription were equally effective, resulting in weight loss of around 2 kg, improvement in HbA1c at 0.3–0.5%, and reduction in insulin daily dose by around 10 units.

### 0160

### A local retrospective cohort of subjects with thyrotoxic heat disease

Thyroid and Osteoporosis

C. L. Wong, H. K. V. Tam, L. M. Fung, Y. K. D. Chan and P. K. E. Lam

*Department of Medicine and Geriatrics, Caritas Medical Centre, Hong Kong, China*

OBJECTIVES: To estimate the proportion of subjects with thyrotoxic heart disease and to investigate the clinical characteristics and outcomes associated with thyrootoxic atrial fibrillation and heart failure.

METHOD: This was a retrospective cohort study performed in the Department of Medicine and Geriatrics, Caritas Medical Centre from year 2004 to year 2013. A total of 126 patients, 28 to 91 years old, presenting with thyrotoxicosis and related cardiac complications were identified by using the Clinical Data Analysis and Reporting System using International Classification of Diseases 9 coding. Information on demographic data, thyroid status, presenting cardiac condition, echocardiographic findings and treatment of the cardiac conditions were retrieved. Factors associated with persistence of thyrotoxic atrial fibrillation, left ventricular systolic dysfunction at presentation and its persistence after euthyroidism were examined.

RESULTS: Among 1755 patients who had a registered diagnosis of thyrotoxicosis, 115 (6.6%) patients presented with atrial fibrillation, 64 (3.6%) patients presented with congestive heart failure and 54 (3.1%) patients presented with coexisting atrial fibrillation and congestive heart failure. Among those 115 patients who had thyrotoxic atrial fibrillation, 42 (36.5%) patients had persistent atrial fibrillation. History of smoking (adjusted odds ratio 8.4. 95% CI [1.4, 12.7], $P$ = 0.011), moderate to severe tricuspid regurgitation (adjusted odd ratio 4.9, 95% CI [1.1, 22.1], $P$ = 0.041) and a larger left atrial diameter (adjusted odd ratio 2.5, 95% CI [1.2, 5.4], p=0.019) were associated with persistence of atrial fibrillation on multivariate logistic regression. Subjects with LV systolic dysfunction were significantly younger (mean age 59 ± 16 years vs 70 ± 15 years, 95% CI [−20, −3], $P$ = 0.007) and were less likely to have hypertension (22.7% vs 54.1%, $P$ = 0.019) on univarate analysis while the presence of dilated cardiomyopathy (57.1% vs 0%, $P$ = 0.011) was the only clinical factor associated with persistent LV systolic dysfunction. These factors became non-significant on multivariate analysis.

CONCLUSION: Persistence of thyrotoxicosis related heart disease occurred in nearly 40% of patients in the present study despite restoration of euthyroidism. History of smoking, moderate to severe tricuspid regurgitation and larger left atrial diameter might suggest a higher risk of persistent atrial fibrillation. Clinical factors associated with persistent left ventricular systolic dysfunction remained to be determined. Prospective study is warranted to further evaluate the predictor factors of persistent left ventricular systolic dysfunction.

### 0161

### Incidence of diabetes and dyslipidemia among Japanese patients treated with olanzapine

Clinical Diabetes

T. Kondo[1], A. Sako[2], K. Nonogaki[2], T. Mitsutsuka[2], H. Nanasawa[1], Y. Uju[1], T. Ito[1], T. Enomoto[1], T. Hayakawa[1] and H. Yanai[2]

*[1]Department of Psychiatry, Kohnodai Hospital, National Center for Global Health and Medicine, Ichikawa, Chiba, Japan, [2]Department of Internal Medicine, Kohnodai Hospital, National Center for Global Health and Medicine, Ichikawa, Chiba, Japan*

OBJECTIVE: Second-generation antipsychotics are known to have metabolic side effects. Most of previous studies on these metabolic side effects have been reported from Western countries. We investigated the incidence and risk factors of diabetes and dyslipidemia among Japanese patients prescribed olanzapine.

METHOD: Eligible cases were patients who received olanzapine ≥28 days between November 2011 and October 2013, and had laboratory data before and after the start of olanzapine until 12 months after the end of prescription. We retrospectively reviewed clinical charts and collected the demographic and laboratory, and prescription data. We used Kaplan-Meier analysis to estimate the cumulative incidence of 'possible diabetes (diabetes cannot be ruled out)' (HbA1c 6.0–6.4%), 'diabetes (strongly suspected of having diabetes)' (HbA1c ≥ 6.5%), hypertriglyceridemia (triglyceride ≥150 mg/dL), high LDL-cholesterol (LDL-cholesterol ≥140 mg/dL) and low HDL-cholesterol (HDL-cholesterol <40 mg/dL). We studied the risk factors of each metabolic abnormalities using the log-rank test.

RESULTS: A number of patients eligible for the analysis of incidence of diabetes was 267. Male were 114, mean age was 42.5 years, and mean duration of mental illness was 13.4 years. Eight patients developed diabetes, and cumulative incidence was 2.0 and 7.7% at 12 and 36 months, respectively. Longer duration of mental illness, hyperuricemia and glucose intolerance were significant risk factors for diabetes. Among 212 patients eligible for the analysis of incidence of possible diabetes, 29 patients developed possible diabetes, and cumulative incidence was 8.7 and 30.9% at 12 and 36 months, respectively. Older age, longer duration of mental illness and hypertriglyceridemia were significant risk factors for possible diabetes. A number of patients eligible for the analysis of incidence of hypertriglyceridemia, high LDL-cholesterol and low HDL-cholesterol is 177, 68 and 95, respectively. Among these patients, a number of patients who developed hypertriglyceridemia, high LDL-cholesterol and low HDL-cholesterol is 75, 19 and 18, respectively. Cumulative incidence at 12 months of hypertriglyceridemia, high LDL-cholesterol and low HDL-cholesterol is 39.4, 27.1 and 25.7%, respectively. Older age and longer duration of mental illness were not significant risk factors for dyslipidemia.

CONCLUSION: Our study showed the relatively high incidence of metabolic abnormalities in Japanese patients taking olanzapine. It is necessary to monitor blood glucose, HbA1c and lipid profile regularly while taking olanzapine. Especially in the patients with older age, longer duration of mental illness, it is necessary to pay attention to the occurrence of impaired glucose metabolism.

© 2016 The Authors. Journal of Diabetes Investigation published by Asian Association for the Study of Diabetes (AASD) and John Wiley & Sons Australia, Ltd

### 0162

### A new method for foods education using doctor's restaurant, with cooking live demonstration and real eating low calorie diet and low salt diet

Education and Care Delivery

M. Kosuga[1], H. Tamura[1], M. Obara[1], R. Takahashi[1], S. Fujine[2], K. Sugawara[1], E. Hiradate[1] and H. Takahashi[1]

[1]*Hiroshi Tamura's Hilltop Clinic, Kitakami city Iwate Prefecture, Japan,* [2]*Doctor's Restaurant Ichigeya, Kitakamicity Iwate Prefecture, Japan*

Background: In general out eating menu at restaurant supply high calorie foods and salty foods in Japan. So it is difficult to eat healthy foods when eating out for diabetic patient. In addition to it, it is hard to educate patient how to cook low calorie food and low salt diet. So we opened 'doctor's restaurant Ichigeya' in front of our clinic since September 2013(at Kitakami city Iwate prefecture,Japan).

Objectives: To give more effective foods education for diabetes patients and hypertension patients by cooking live demonstration and real eating healthy foods at doctor's restaurant.

Method: 1. Healthy menu: Many menus supplied at the restaurant are supervised by a physician and registered dietitian. The concepts of the healthy menus are as follows.

Total calorie is less than 600 kilocalorie. The total salt of foods contains less than 2.5 g.

Total amount of the vegetables is over 120 g.

2. Therapy menu: We also supply 'therapy menu' for patients. For example low protein menu for chronic kidney disease patients, high iron menu for anemic patients, low purine body menu for gout patients, low allergen foods for foods allergy patients and so on.

3. Healthy dinner event with cooking live demonstration: We have held healthy dinner event almost 4 times in year. Total number of the dinner event is eight times. In the dinner event, our clinic staff broadcasts the healthy cooking methods by chef from the kitchen of the restaurant to the TV display at the main hall of the restaurant, by using handy video camera. In the dinner event we also present the educational lecture by the doctor and registered dietitian of our clinic about healthy diet using the TV display.

Result: Almost 52% customers ordered the healthy menu in the restaurant. The participant number to the dinner event was 254 (male:24, female230) in total. Mean age was 72 years old(male), and 61 years old (female). Fifteen percent of participants were patients with diabetes. Fifty percent of participants had other disease (hypertension, hyperlipidemia and so on). The results of questionnaire from participants are almost good responses.

Conclusion: The doctor's restaurant supervised by doctor and registered dietitian is effective method for patients who need diet therapy. The healthy menus and healthy cooking live demonstration at the doctor's restaurant helps to understand the appropriate amount of foods and appropriate seasoning for low calorie and low salt diet.

### 0163

### Clinical characteristics of Japanese children with MODY 2 detected by a urine glucose screening at schools

Genetics and Epigenetics

H. Furuta[1], S. Matsuno[1], M. Miyawaki[1], A. Doi[1], S. Uraki[1], H. Ariyasu[1], H. Kawashima[1], M. Nishi[1], K. Nanjo[2] and T. Akamizu[1]

[1]*Wakayama Medical University, Wakayama, Japan,* [2]*Wakayama Rosai Hospital, Wakayama, Japan*

Objectives: Maturity-onset diabetes of the young type 2 (MODY2) results from heterozygous mutations in the glucokinase (GCK) gene. MODY2 should be differentiated from other types of young onset diabetes because management differs. Screening criteria to predict MODY2 is propounded in Caucasian. We evaluated the criteria in Japanese children with MODY2.

Method: Five children (Male/Female: 3/2) from four families, who were diagnosed as having MODY2 in a urine glucose screening at school, were enrolled in this study.

Results: The ages at diagnosis was 9.2 ± 2.9 years (mean ± SD, 7–14 years). All subjects were thinnish and the HOMA-R was 1.0 ± 0.5 (0.49–1.6). The HOMA-β and an insulinogenic index on the oral glucose tolerance test (OGTT) were 24.0 ± 14.7 (11.6–48) and 0.4 ± 0.3 (0.14–0.9), respectively. It has been reported in Caucasian that people with MODY2 have mild hyperglycemia such as a fasting glucose between 5.4 and 8.3 mmol/L and HbA1c between 5.8 and 7.6%. In our patients, the fasting glucose and HbA1c were also ranged 6.0–6.9 mmol/L and 5.9〜6.5%, respectively. It has also been reported that a 2-h glucose increment during an OGTT is ≦3 mmol/L in MODY2. In our patients, the increment was ranged 0.2–3.6 mmol/L. Almost MODY2 patients have inherited the gene mutation from an affected parent. In three of our four families, one of parents had been diagnosed as having mild hyperglycemia. Nobody was treated by either oral hypoglycemic drug or insulin.

Conclusions: The screening criteria of MODY2 propounded in Caucasian is also useful in Japanese children.

### 0164

### Anti-diabetic effects and actions of dwarf lilyturf tuber (maidong) in high fat diet induced obese mice

Insulin Secretion

D. Mao[1], Ronald, C. W. Ma[2], C. S. Lau[3], C. C. Wang[1] and W. H. Tam[1]

[1]*Department of Obstetrics & Gynaecology, The Chinese University of Hong Kong, Hong Kong,* [2]*Division of Endocrinology & Diabetes, Department of Medicine & Therapeutics, The Chinese University of Hong Kong, Hong Kong,* [3]*Institute of Chinese Medicine, School of Chinese Medicine, The Chinese University of Hong Kong, Hong Kong*

Our objective is to investigate the effects of polysaccharide extract from Dwarf Lilyturf Tuber (Maidong) (MP) on insulin resistance and β cell function using high fat diet (HFD) induced obese mice model. C57BL6J mice were randomly allocated into 3 groups after being fed with 10 weeks of HFD (60%cal lard). Over the next 4 weeks, groups 1 & 2 were then treated with MP at 200 (1× clinical dose) & 400 (2× clinical dose) mg/kg body weight respectively, and group 3 received water as control while they continued on HFD. C57BL6J mice, being fed with normal diet for 14 weeks, served as non-diabetic control (group 4).

Then, all mice underwent an insulin tolerance test (ITT) followed by an oral glucose tolerance test (OGTT) on the following day, which were performed after 6 h and 15 h overnight fast respectively. Blood samples were taken from tail vein at 0, 30, 60 and 120 min in both tests. Serum glucose and insulin levels were compared between groups using repeated measures of ANOVA and area under the curve. There was no significant change in the glucose profile after MP treatment in both ITT and OGTT. However, insulin levels were significantly higher at OGTT in both groups 1 ($P = 0.03$) and group 2 ($P = 0.003$) to group 3. In summary, we demonstrate an increase in insulin secretion, but without improvement in insulin resistance and glucose levels after treatment with MP on diabetic HFD obese mice. We speculate that MP might contain some active components which may amplify the pancreatic β cell function.

### 0167

### Comparison of abdominal visceral adipose tissue area measured by computed tomography with that estimated by bioelectrical impedance analysis method in Korean subjects

Obesity

D.-H. Lee[1], K. S. Park[1], S. Ahn[2], E. J. Ku[3], K. Y. Jung[4], Y. J. Kim[1], K. M. Kim[1], J. H. Moon[1], S. H. Choi[1], K. S. Park[5], H. C. Jang[1] and S. Lim[1]

[1]*Seoul National University Bundang Hospital, Seongnam, Republic of Korea,* [2]*Medical Research Collaborating Center of Seoul National University Bundang Hospital, Seongnam, Republic of Korea,* [3]*Chungbuk National University Hospital, Cheongju, Republic of Korea,* [4]*Eulji University Hospital, Seoul, Republic of Korea,* [5]*Seoul National University College of Medicine, Seoul, Republic of Korea*

Objectives: We evaluated the concordance between visceral fat area (VFA) estimated by bioelectrical impedance analysis (BIA) or computed tomog-

© 2016 The Authors. Journal of Diabetes Investigation published by Asian Association for the Study of Diabetes (AASD) and John Wiley & Sons Australia, Ltd raphy (CT) in Korean subjects with a wide range in age and body mass index (BMI).

Method: In 1006 individuals (mean age 55.2 ± 11.8 [19–87] years, mean BMI 26.0 ± 3.5 [17–46] kg/$m^2$, 48.9% men), VFA quantified by CT was compared with VFA using a multifrequency BIA machine within 15 days. Concordance rates were compared by age or BMI using correlation analysis, Bland-Altman plots, and intraclass correlation coefficient (ICC). Using BIA data, we established a regression formula to reflect CT-VFA.

Results: The mean VFAs by CT and BIA were 131.9 ± 57.3 $cm^2$ and 110.5 ± 33.9 $cm^2$, respectively ($r$ = 0.605, P < 0.001). The mean difference was 21.4 ± 45.6 $cm^2$, tending to increase with BMI. In women with BMI < 25 kg/$m^2$ or age < 50 years, the VFAs by BIA were similar to those by CT (ICC = 0.496 in BMI < 25 kg/$m^2$ and ICC = 0.638 in age < 50 years). However, the difference was greater in men with BMI ≥ 25 kg/$m^2$ or age ≥ 50 years. Applying our formula, the difference between estimations decreased to 12.0 ± 39.8 $cm^2$.

Conclusions: VFA estimated by BIA correlated well with that by CT, but a more accurate formula is needed to match CT data, particularly in older men or subjects with a high BMI.

### 0169

### The usefulness of newly long acting insulin degludec for 2 years in japanese type 1 diabetes

Therapeutics of Diabetes

T. Miyakawa, Y. Kawagoe, M. Nogawa, H. Yoshimura, M. Hasegawa, Y. Watanabe and H. Fujii

*Tama-center Mirai Clinic, Tama Tokyo, Japan*

Objective: We have used the newly long acting insulin as Degludec for 69 Type 1 diabetes outpatients for 2 years. And 29 outpatients of them we performed CGMS (Continuous Glucose Monitoring System; Medtronic iPro™2). To assess the usefulness of Degludec (1) for 2 years and (2) for daily profile used CGMS.

Subjects and Methods: To analyze (1) the time course of HbA1c, body weight, insulin dose for 2 years in type 1 outpatients, who switch from insulin Glargine ($n$ = 36;GroupA) or insulin Detemir ($n$ = 28;GroupB) to insulin Degludec. $n$ = 69, 53.0 ± 16.8 y/o, BMI 21.6 ± 2.9. C-peptide 0.24 ± 0.41 ng/dL, (2) the time course of blood glucose profile who switch from insulin Glargine or insulin Detemir to insulin Degludec, by CGMS) at the time of switching ($n$ = 19) and 1–3 months after switching ($n$ = 10). We investigated indicators of the dawn phenomenon (DP); ⊿BG (pre-breakfast BG minus minimum BG at 2 to 6 AM).

Results: (1) The mean HbA1c was significantly improved from 7.94 ± 1.03 to 7.59 ± 1.03 ($P$ < 0.0001)%.Body weight was slightly increased from 56.1 to 57.0 kg ($P$ < 0.01). 31 cases have continued improvement of >0.5% of HbA1c level without severe hypoglycemia. 28 cases were not changed HbA1c level, but were decreased hypoglycemia ($P$ < 0.05). Only 7 cases were worsened >0.5%. 3 cases were dropout or changed insulin.

Group A; The mean HbA1c was improved slightly from 8.00 to 7.80 ($P$ < 0.01)%. Body weight was slightly increased. Insulin doses was not changed.

Group B: The mean HbA1c was from 7.86 to 7.58 ($P$ < 0.01)%. Body weight was not changed. Insulin doses was decreased from 16.8 to 11.7 μ/day.

(2) Frequency of nocturnal ($P$ < 0.01)and daytime ($P$ < 0.05) hypoglycemia was decreased than former insulins by CGMS. Nighttime and daytime hypoglycemia was correlated with ⊿BG ($P$ < 0.01). ⊿BG and CPR were correlated ($P$ < 0.03).

Conclusion: (1) Degludec is useful to improve HbA1c level, decrease hypoglycemia switching from Glargine and Detemir in Japanese Type1 diabetes patients.

(2) Frequency of nocturnal hypoglycemia was decreased. However, Dawn phenomenon during use is dependent on residual intrinsic insulin secretions, and the patients with more marked Dawn phenomenon tended to experience hypoglycemia during the day or night. Careful titration of the stable insulin degludec is required in response to fasting blood glucose levels in type 1 diabetes.

(3) As a result 45% of patients have continued improvement of >0.5% of HbA1c level without severe hypoglycemia,40% was not changed HbA1c, but decreased hypoglycemia. The titration of their treatment have done carefully about fasting blood glucose level for 2 years.

### 0171

### Plasma acylated ghrelin is inversely associated with blood pressure and fasting blood glucose in Chinese adults with metabolic syndrome

Prediabetes and Metabolic Syndrome

F. N. Ugwu, X. M. Pei, A. P. Yu, V. Ma and P. M. Siu

*The Hong Kong Polytechnic University, Hong Kong, Hung Hom, Kowloon, Hong Kong*

Objectives: Ghrelin is a gut peptide that controls different aspects of metabolism. Acylated and unacylated ghrelin are the two principal forms of the peptide but acylated ghrelin is the active form in circulation. Plasma ghrelin levels may be altered in individuals suffering from metabolic syndrome. Nonetheless, the orexigenic influence of ghrelin with the risk factors remains to be elucidated. Therefore, this study aimed to examine whether the manifestation of metabolic syndrome might be related to the circulating acylated ghrelin level. The association of plasma ghrelin with risk factors including central obesity, hypertension, dyslipidemia and hyperglycemia was also determined.

Methods: Subjects consisted of Hong Kong Chinese adults (n = 70; mean ± SD age = 56 ± 9.4 years), divided according to the National Cholesterol Education Program (NCEP) criteria into three groups: non-metabolic syndrome non-obese (NMNO), non-metabolic syndrome obese (NMO), and metabolic syndrome obese (MSO). Informed consent was obtained from the subjects and thereafter, systolic blood pressure (SBP), diastolic blood pressure (DBP), waist circumference, fasting plasma glucose, triglycerides, high-density lipoprotein cholesterol and acylated ghrelin were assessed.

Results: The results revealed that there was a statistically significant difference in the plasma ghrelin levels among groups as determined by one-way ANOVA ($P$ = 0.043). Post hoc analysis showed that the mean plasma ghrelin level of the NMNO subjects was significantly higher than the MSO participants ($P$ = 0.020) but the ghrelin level was not statistically different between the NMNO and NMO subjects. Pearson's correlation analysis revealed a statistically significant negative correlation between plasma ghrelin level and SBP ($r$= –0.397; $P$ = 0.001), DBP ($r$=–0.252; $P$ = 0.035) and glucose level ($r$= –0.298; $P$ = 0.012). Negative trends between ghrelin and triglycerides ($r$= –0.205; $P$ = 0.088) and waist circumference ($r$= –0.204; $P$ = 0.090) were observed.

Conclusions: These results suggest that plasma acylated ghrelin level is related to the diagnostic risk factors of metabolic syndrome. Additional research is needed to further dissect the exact role of plasma ghrelin in metabolic syndrome as well as hypertension and type 2 diabetes mellitus.

### 0172

### Influences of withdrawal and dose reduction of pioglitazone on metabolic parameters in patients with type 2 diabetes: a chart-based analysis

Therapeutics of Diabetes

H. Katsuyama[1], T. Fukunaga[2], H. Hamasaki[1], H. Adachi[1], S. Moriyama[1], M. Kobayashi[1], C. Hiraishi[1], A. Kawaguchi[1], A. Sako[1], S. Mishima[1] and H. Yanai[1]

*[1]Department of Internal Medicine, Kohnodai Hospital, National Center for Global Health and Medicine, Ichikawa, Chiba, Japan, [2]Department of Gastroenterology, Kohnodai Hospital, National Center for Global Health and Medicine, Ichikawa, Chiba, Japan*

Objectives: Since two epidemiological studies showed that pioglitazone might be associated with an increased risk of bladder cancer in 2011, pioglitazone was withdrawn, and its daily dose was reduced in many type 2 diabetic patients. The aim of this study is to clarify the influences of withdrawal or dose reduction of pioglitazone on metabolic parameters in type 2 diabetic patients.

Method: We selected Japanese patients with type 2 diabetes, who had underwent withdrawal (Group A) or daily dose reduction (Group B) of pioglitazone after a continuous prescription for 3 months or longer between January 2010 and March 2014. We compared the data before the withdrawal or dose reduction of pioglitazone with the data at 3 or 6 month after the withdrawal or dose reduction by a chart-based analysis.

Results: Pioglitazone was withdrawn in 20 patients, and the daily dose of pioglitazone was reduced in 51 patients. The mean body mass index was 28.5 kg/$m^2$ in group A and 27.7 kg/$m^2$ in group B. The mean pioglitazone dose at baseline was 23 mg in subjects of group A, and 30 mg in subjects of

© 2016 The Authors. Journal of Diabetes Investigation published by Asian Association for the Study of Diabetes (AASD) and John Wiley & Sons Australia, Ltd group B. The mean dose of pioglitazone was 12 mg at 3 and 6 months after the dose reduction in group B. The number of patients who took high-dose metformin (≥1000 mg) or dipeptidyl peptidase-4 (DPP-4) inhibitors increased after the withdrawal or dose reduction in both groups. Although body weight tended to decrease in both groups, no significant change was observed in plasma glucose and HbA1c levels. The serum high-density lipoprotein-cholesterol levels significantly decreased at 3 and 6 months after the withdrawal and at 6 months after the dose reduction of pioglitazone. Moreover, the serum alanine aminotransferase levels significantly increased 6 months after the withdrawal of pioglitazone in group A.

CONCLUSION: Our study suggests that the withdrawal or dose reduction of pioglitazone might exacerbate the lipid profile and liver function in patients with type 2 diabetes, although the glycemic control could be maintained by using high-dose metformin or DPP-4 inhibitors.

### 0173

### To identify regulators of endometrial mesenchymal-like stem cells during menstruation

Reproductive Endocrinology

S. Xu, R. W. S. Chan, E. H. Y. Ng and W. S. B. Yeung
*The University of Hong Kong, Hong Kong*

Human endometrium is known for its cyclical regeneration during each menstrual cycle. Somatic stem cells are believed to play a role in the regeneration process. We hypothesized that soluble factors secreted during menstruation are involved in the regulation of endometrial mesenchymal-like stem cells (eMSCs).

EMSCs were isolated by sequential beading for the two perivascular markers CD140b and CD146. The CD140b+CD146+ cells were indirect co-cultured with freshly isolated endometrial niche (epithelial or stromal) cells in a culture insert or treated with spent medium from culture of endometrial samples at menstrual phase. Cloning efficiency and the phenotypic expression of the eMSC markers after 15 days of culture were evaluated.

There was a higher trend of eMSCs from menstruation and proliferative samples compared to secretory samples. In the clonogenic assay, co-culture of eMSCs with menstruation niche cells significantly increased the colony forming ability (control 1%, epithelial 14.75%, $P < 0.001$, stromal 5.97%, $P < 0.01$) and the phenotypic expression of the eMSC markers (control 1%, epithelial 1.58%, $P < 0.1$, stromal 1.63%, $P < 0.001$). EMSCs from menstrual phase underwent more rounds of self-renewal (5.25 ± 0.96 rounds) than those from proliferative and secretory phases (3.33 ± 0.82 rounds, $P < 0.05$). Interestingly, co-culture with niche cells from secretory phase did not increase the cloning efficiency of eMSC or maintain the eMSC phenotypic markers.

The clonogenic ability decreased (control 1.00%, epithelial CM 0.51%, $P < 0.0001$, stromal CM 0.46%, $P < 0.0001$), but the expression of eMSC markers (CD140b+CD146+) increased (control 1%, epithelial CM 1.58%, $P < 0.001$, stromal 1.52%) when eMSCs were cultured in medium supplemented with conditioned medium from endometrial epithelial or stromal cells at menstrual phase. Western blotting assay indicated an elevated level of both active β-catenin (fold change: epithelial 4.43, $P < 0.05$, stromal 4.03, $P < 0.05$) and total β-catenin (fold change: epithelial 1.51, $P < 0.05$, stromal 1.58, $P < 0.05$) from clonally derived cells after co-culture. Analysis with cytokine array showed that the level of five cytokines namely, GRO-α, GM-CSF, IL-6, CXCL-5 and MCP3, in the culture medium changed significantly after co-cultured with menstruation niche cells.

In conclusion, eMSC population is enriched during menstruation. Soluble factors secreted by menstruation niche cells such as the identified candidate cytokines may change the proliferative potential of the eMSCs and maintain their phenotype. Wnt-β-catenin signaling pathway may be involved in the activation of the eMSCs during menstruation.

### 0175

### DNA methylation: association with metabolic syndrome and the risk of colorectal cancer

Therapeutics of Diabetes

X. Wang, X. Pei, S. S. Ng, B. Y. Yung, L. W. Chan, C. S. Wong and P. M. Siu
*Department of Health Technology and Informatics, Faculty of Health and Social Sciences, The Hong Kong Polytechnic University, Hong Kong*

OBJECTIVE: Metabolic syndrome (MetS) has an increasing prevalence in the general population. A large body of epidemiologic studies indicates that the risk of colorectal cancer (CRC) is elevated in people with MetS. However, the association of MetS and the risk of colorectal cancer has not yet been well examined. In particular, it remains unclear what mechanism would account for such an association. The aim of this study was to investigate the influence of MetS on gene promoter DNA methylation and the involvement of the epigenetic modification in the risk of colorectal cancer.

METHODS: Genome-wide DNA methylation profiling was performed with colon tumor tissue and the corresponding tumor adjacent normal tissue from 10 CRC patients without MetS and 10 CRC patients with MetS. The Illumina Infinjum 450 k Human DNA Methylation Beadchip was used to obtain DNA methylation profiles across 485,577 cytosine loci in the human genome. The differential methylation loci (DML) within the promoter region of genes were compared using false discovery rate (FDR) threshold set at 0.05 and 0.1. The number of DML in the vicinity of gene transcription starting sites correlated with the level of expression of some genes which have been linked to carcinogenesis. We analyzed the relationship between RNA expression and DNA methylation at the promoter regions of genes by using real time PCR.

RESULTS: 8 DML were identified in CpGs using FDR threshold of 0.05, while 3,224 DML were revealed using FDR threshold of 0.1 when the methylation of individual CpGs were analyzed in tumor tissue and the adjacent normal tissue from CRC patients without MetS. Meanwhile, 929 DML were identified in CpGs using FDR threshold of 0.05, and 3,489 DML were revealed using FDR threshold of 0.1 when the methylation of individual CpGs were analyzed in tumor tissue and the adjacent normal tissue from CRC patients with MetS. An overlap of 821 CpGs DML were found between the CRC patients with and without MetS. Several candidate genes were identified to be dysregulated in tumor tissue of CRC patient with MetS.

CONCLUSIONS: Our data demonstrate an association between DNA methylation status of CpG sites located in genes with MetS and the risk of colon and rectal cancers. These results might shed light on the use of DNA methylation status of CpG sites in genes as predictor of colon and rectal cancer in patient with MetS.

### 0176

### Differential lipidomic signatures of human fat tissues according to their location and the subject's metabolic state

Integrated Physiology

T. J. Oh[1,2], M. Y. Lee[2], J. W. Lee[2], K. M. Kim[1,2], S. Lim[1,2], H. C. Jang[1,2] and S. H. Choi[1,2]
[1]*Seoul National University College of Medicine, Seoul, Republic of Korea,* [2]*Seoul National University Bundang Hospital, Seongnam, Republic of Korea*

OBJECTIVE: We investigated the differences of subcutaneous, visceral and pericardial lipidomes and compared their lipidomic signatures between subjects with normal glucose tolerance (NGT) and type 2 diabetes mellitus (T2DM).

METHODS: Liquid chromatography/mass spectrometry was used for lipid profiling in different fat tissues from patients with NGT ($n$ = 24) and T2DM ($n$ = 27) who underwent coronary artery bypass graft. Principal component analysis was performed to compare lipidomic signatures between tissues and between patient groups.

RESULTS: Total 26 lipid classes and more than 400 lipid species were identified and selective enrichment was observed in each fat tissue. Pericardial fat had distinct composition of ceramide, diacylglycerol, and triacylglycerol from subcutaneous and visceral fat. However, lipid species of phosphatidylcholine, sphingomyelin, and lysophosphatidic acid seemed to be comparable between pericardial and subcutaneous fat.

© 2016 The Authors. Journal of Diabetes Investigation published by
Asian Association for the Study of Diabetes (AASD) and John Wiley & Sons Australia, Ltd

There was no significant difference on lysophosphatidylcholine species among three fat tissues. In addition, NGT showed noticeable different diacylglycerol composition in subcutaneous, visceral and pericardial fat from subjects with T2DM. Triacylglycerol composition also showed differential lipidomic signatures in subcutaneous and visceral fat between two groups.

CONCLUSION: In this lipidomic study, we observed different lipidomic signatures in differently located fat tissues and differentially expressed lipids were defined according to subject's metabolic state. Further validation of the study will provide a novel insight for characteristics of ectopic fat in subjects with T2DM.

### 0177

### Effectiveness of alternate-day administration of SGLT2-inhibitors in Japanese patients with type 2 diabetes

Therapeutics of Diabetes

Y. Wada, Y. Hamamoto, S. Honjo, Y. Kawasaki, S. Tokumoto, E. Okamura, E. Yamaguchi, J. Fujikawa and A. Hamasaki

*The Tazuke Kofukai Foundation Medical Research Institute Kitano Hospital, Osaka, Japan*

BACKGROUND: Enhanced urinary glucose excretion by Sodium Glucose co-Transporter-2 (SGLT-2) inhibitors reportedly sustain more than 24 h. Although their effects, especially on body weight reduction, are highly expected, costs and adverse events such as dehydration or urinary tract/genital infections make it difficult to use in clinical practice. In this study, we investigated the efficacy of alternate-day administration of SGLT-2 inhibitor compared with daily administration.

SUBJECTS AND METHODS: This study included a total of 14 patients (male 9, female 5; mean age: 56.1 ± 2.4 S.E. years, mean BMI: 28.7 ± 1.0 kg/m$^2$, mean A1C: 8.3 ± 0.2%) with type 2 diabetes whose HbA1c was more than 7.0%. The patients were randomly assigned to take SGLT-2 inhibitor every other day ($n$ = 5: group A) or once daily ($n$ = 9: group B). Their A1C levels and body weight were measured before and 2 months after SGLT-2 inhibitor administration.

RESULTS: A1C levels were decreased in 60% of patients in group A (–0.6 ± 0.5%) and 66% of patients in group B (–0.7 ± 0.3%). Body weight reduction was observed in 80% of patients in group A (–1.3 ± 0.6 kg) and all in group B (2.3 ± 0.2 kg). There were no significant differences between two groups in A1C and body weight.

CONCLUSION: Alternate-day administration of SGLT-2 inhibitor may have similar effects to daily administration.

### 0179

### What is decreased in the early phase of sglt2 inhibitor treatment, body fat, skeletal muscle, or water? Analysis by the multi-frequency bioelectrical impedance method

Therapeutics of Diabetes

H. Nakayama, S. Yoshinobu, S. Kawano, M. Tsuruta, S. Iwata, M. Nohara, R. Soga, S. Akasu, Y. Goto, N. Wada, Y. Tajiri and K. Yamada

*Kurume University, Kurume, Japan*

OBJECTIVES: SGLT2 inhibitors increase urinary glucose excretion resulting in the improvement of glycemic control and the reduction of body weight. The body weight loss is most marked soon after the initiation of SGLT2 inhibitor therapy. However, it is not clear whether the early loss of weight indicates the reduction of body fat. In this study we assessed the changes in body composition together with those in metabolic parameters during the first week of SGLT2 inhibitor treatment.

METHODS: The subjects of this study were 30 inpatients with type 2 diabetes, aged 55 ± 11 years, with a body mass index of 29.3 ± 5.4 kg/m$^2$. After an approximately 7-day stabilizing period, 50 mg of ipragliflozin or 20 mg of tofogliflozin was administered to each patient. The iPro™ 2 CGM devices were used to monitor subcutaneous glucose levels. Body composition was measured by a multi-frequency bioelectrical impedance analyzer (InBody™720) in 14 of the subjects. The patients ate a standardized low-fat meal and drank at least 500 mL of extra water.

RESULTS: Average glucose levels determined by CGM were decreased from 188 ± 51 mg/dL to 144 ± 31 mg/dL accompanied by a decline in body weight of 1.6 kg during the SGLT2 inhibitor treatment. Furthermore, serum levels of AST, ALT, LDL-cholesterol, HDL-cholesterol, and triglyceride were significantly reduced. The body composition analysis showed that intracellular water, body protein, body mineral, body fat, and skeletal muscle were decreased by 0.36 kg, 0.15 kg, 0.06 kg, 1.14 kg, and 0.43 kg, respectively, while extracellular water did not change. The decrement in skeletal muscle was highly correlated with those in intracellular water ($r$ = 0.96) and body protein ($r$ = 0.93).

CONCLUSIONS: These observations showed that the decline in body weight during the first week of SGLT2 inhibitor treatment was mainly attributable to the reduction in body fat. In this study, dehydration was modest indicating that the subjects took enough water to compensate polyuria. Nevertheless, intracellular water was significantly decreased during the treatment. The strong correlation between the decrement in skeletal muscle and that in intracellular water suggests that the loss of skeletal muscle volume soon after the initiation of SGLT2 inhibitors was at least in part caused by the loss of intracellular water, although body protein slightly but significantly decreased during the period.

### 0181

### Wnt/β-catenin signaling is associated with cancer stem cell properties of cervical cancer CD133+ subpopulation

Endocrine Tumours

C. K. Yuen, Z. Niu, W. Zhu, R. T. Pang and W. S. Yeung

*Department of Obstetrics and Gyneacology, Faculty of Medicine, HKU, Pokfulam, Hong Kong*

BACKGROUND: The cancer stem cells (CSCs) theory hypothesizes that CSCs contribute only a small population in cancer tissue but are responsible for tumor initiation. The World Health Organization states that cervical cancer is the third most common cancer in the female population. We recently demonstrated that oncogenic microRNA-135a transforms human papillomavirus infected epithelial cells to CSC-like cells through activating Wnt/β-catenin signaling. This cell subpopulation express CSC surface maker CD133 and pluripotent gene Oct4. These CD133+ subpopulation possessed higher tumor formation capacity and self-renewal ability. The Wnt/β-catenin signaling pathway is important to cell cycle control and the expression of stem-cell related genes is associated with CSCs properties., however, whether Wnt/β-catenin signaling is essential for CSC subpopulation maintenance is unknown.

OBJECTIVE: To investigate the involvement of Wnt/β-catenin signaling in CSC properties of microRNA-135a induced CSC-like CD133+ subpopulation.

METHOD: Wnt/β-catenin stimulator, human recombinant Wnt3a protein (rhWnt3a), and inhibitor, XAV939, were used to modulate Wnt/β-catenin signaling. Oct4, CD133, multi-drug resistant genes expression and self-renewal ability were measured under modulation of Wnt/β-catenin activity. Self-renewal ability was estimated by tumorsphere formation assay and expression of CSC-associated genes was measured by RT-qPCR.

RESULTS: Wnt/β-catenin signal activation increased the expression of Oct4 and CD133 in cervical cancer cell lines including HeLa, C33A and NC104 E6/E7-135a. In microRNA-135a induced CD133+ subpopulation, the tumorsphere formation ability was affected by the level of β-catenin. rhWnt3a stimulation increased both the number (112%, $n$ = 4, $P < 0.05$) and size (116%, $n$ = 8, $P < 0.05$) of tumorspheres formed by CD133+ cells while inhibition by 5 μM XAV939 decreased the number (77%, $n$ = 8, $P < 0.001$) and size (78%, $n$ = 8, $P < 0.05$) of tumorspheres. In addition, expression levels of CD133, Oct4, ATP-Binding Cassette B1 (ABCB1)/G2 (ABCG2) and Aldehyde dehydrogenases (ALDH1A1 and ALDH1A3) in tumorspheres were positively related to β-catenin level.

CONCLUSION: Activation of Wnt/β-catenin signaling increased the transcription of Oct4 and CD133 in cervical cancer cells lines. Expression of CSC-associated genes were positively related to the level of β-catenin in CD133+ subpopulation. These results suggested that CSC properties in CD133+ cells were associated to Wnt/β-catenin signaling.

© 2016 The Authors. Journal of Diabetes Investigation published by Asian Association for the Study of Diabetes (AASD) and John Wiley & Sons Australia, Ltd

### 0183

### Serum high-sensitivity c-reactive protein levels predict decline in kidney function among chinese patients with type 2 diabetes mellitus

Microvascular Complications

C. Lee[1], Y. Woo[1], C. Yeung[1], W. Chow[1], M. Yuen[1], C. Fong[1], A. Xu[1,3] and K. Lam[1,2]

[1]*Department of Medicine, The University of Hong Kong, Hong Kong,* [2]*Research Centre of Heart, Brain, Hormone and Healthy Aging, The University of Hong Kong, Hong Kong,* [3]*State Key Laboratory of Pharmaceutical Biotechnology, The University of Hong Kong, Hong Kong*

BACKGROUND: Elevated serum high-sensitivity C-reactive protein (hsCRP) levels have been demonstrated, from cross-sectional studies, to be associated with diabetic nephropathy. A recent Asian study also suggested serum hsCRP levels predicted risk of development, but not progression, of diabetic nephropathy. We investigated whether serum hsCRP was predictive of the decline in renal function among Chinese type 2 diabetic subjects.

METHOD: Baseline serum hsCRP levels were measured in 1101 Chinese type 2 diabetic subjects recruited from the Hong Kong West Diabetes Registry. The role of serum hsCRP in predicting decline in estimated glomerular filtration rate (eGFR) over a median follow-up of 4 years was analyzed using Cox regression analysis. The eGFR of study subjects were calculated using the 2009 Chronic Kidney Disease Epidemiology Collaboration (CKD-EPI) formula and eGFR decline was defined according to the International Society of Nephrology recommendation statements.

RESULTS: Amongst 1101 subjects with baseline eGFR ≥30 mL/min/1.73 $m^2$, serum hsCRP levels were significantly higher in those with eGFR decline during follow-up ($n$ = 254) than those without decline ($n$ = 847) (1.8 mg/L [0.9–4.1] vs. 1.3 [0.5–3.2]; p <0.001). On multivariable Cox regression analysis, baseline serum hsCRP levels were independently associated with eGFR decline (hazard ratio [HR]: 1.14; 95% confidence interval [95% CI] 1.02–1.26; $P$ = 0.019) even after adjustment for age, duration of diabetes, waist circumference, hypertension, glycated hemoglobin A1c, baseline eGFR and albuminuria status of study subjects.

CONCLUSIONS: Serum hsCRP might also be a useful biomarker for predicting decline in kidney function among Chinese type 2 diabetic patients.

### 0184

### The role of galectin-3 in sperm-zona pellucida interaction

Reproductive Endocrinology

S. Mei[1], C. L. Lee[1,2], K. K. W. Lam[2], W. S. B. Yeung[1,2] and P. C. N. Chiu[1,2]

[1]*The University of Hong Kong, Hong Kong,* [2]*Queen Mary Hospital, Hong Kong*

In order to acquire fertilization ability, human spermatozoa have to undergo post-testicular maturation in the epididymis and during ejaculation. This sperm maturation process is followed by further biological changes during capacitation in the female genital tract. These maturation processes require modification and reorganization of molecules on the sperm plasma membrane. Human fertilization begins when a capacitated spermatozoon binds to the ZP (zona pellucida) of an egg. The binding is initiated by the interaction of ZP-binding protein(s) on the plasma membrane with glycans on the ZP glycoproteins. Defective spermatozoa-ZP binding causes infertility and is a major cause of low fertilization rates in clinical in-vitro fertilization

Galectin-3 is a secretory carbohydrate-binding protein that can cross-link glycoproteins together as a complex on the cell surface. It is well characterized for mediating cell adhesion, cell surface receptor trafficking and clustering. In the male reproductive tract, galectin-3 has been identified in epididimis, seminal plasma and ejaculated spermatozoa, but not in germ cells, suggesting that the ejaculated spermatozoa acquire galectin-3 post-testicularly. However, the role of galectin-3 in sperm functions is still unknown. In this study, we hypothesize that galectin-3 plays a modulatory role in spermatozoa-ZP binding.

Immunostaining results showed that galectin-3 immunoactivity was localized to the apical region of capacitated spermatozoa, a region known for ZP binding. Simultaneous staining with CD46 and wheat germ agglutinin confirmed its localization on sperm plasma membrane. Blocking the function of sperm galectin-3 by functional blocking antibody or galectin-3 competitive substrate (i.e. N-Acetyl-D-lactosamine) inhibited spermatozoa-ZP binding as demonstrated by hemizona assay. On the other hand, no effect can be observed for the antibody/N-Acetyl-D-lactosamine on sperm motility, viability and acrosome reaction as demonstrated by computer assisted sperm analysis、trypan blue staining and FITC-Pisum sativum agglutinin staining respectively. Furthermore, galectin-3 was demonstrated to be present in seminal plasma in form of extracellular membranous vesicles, suggesting that galectin-3 may be transferred to sperm surface by extracellular vesicles during post-testicular maturation. Our results provide evidences that protein from the male reproductive tract may be important for fertilization in human.

ACKNOWLEDGEMENTS: This work was supported in part by grants from the Committee on Research and Conference Grant, Hong Kong Research Grant Council (764512M and 17105214).

### 0185

### The role of Sialyl-Lewis(x)-binding protein and sialidase in spermatozoa-zona pellucida interaction

Reproductive Endocrinology

Y. Wang[1], C.-L. Lee[1,2], K. K. W. Lam[1], S. Mei[1], C.-W. Wong[1], W. S. B. Yeung[1,2], E. H. Y. Ng[1,2] and P. C. N. Chiu[1,2]

[1]*Department of Obstetrics & Gynaecology, The University of Hong Kong, Hong Kong,* [2]*Centre of Reproduction, Development and Growth, Queen Mary Hospital, Hong Kong*

A spermatozoon cannot fertilize an oocyte immediately after ejaculation, but acquire the fertilization capacity within the female reproductive tract by a process termed capacitation. This process involves relocalization of molecules in the plasma membrane of the sperm head resulting in acquisition of the binding capacity of the spermatozoa to the outer coat (zona pellucida, ZP) of the oocyte. Capacitated spermatozoa initiate fertilization by binding to the carbohydrate sequences on the ZP, which induces acrosome reaction of the ZP-bound spermatozoa. Defects in this binding are the major causes of reduced fertilization rates in assisted reproduction.

Recently, by using ultrasensitive mass spectrometry, we identify sialyl-Lewis(x) (SLeX) sequence as the most abundant terminal sequence on the glycans of human ZP. It is also the major carbohydrate ligand for human sperm-ZP binding. By using affinity chromatography followed by *mass spectrometric analysis,* chromosome 1 open reading frame 56 (C1orf56) *was identified to be one of the* SLeX-binding proteins in the membrane protein extracts of capacitated spermatozoa. Treatment with antibody against C1orf56 inhibited spermatozoa-ZP binding and ZP-induced acrosome reaction dose-dependently. Anti-C1orf56 antibody did not affect sperm viability, motility and spontaneous acrosome reaction. The acrosomal region of spermatozoa possessed C1orf56 immunoreactivity and its intensity increased after capacitation in vitro, suggesting translocation of C1orf56 to the cell surface during capacitation.

Our data further demonstrated that capacitated human spermatozoa possess cell surface sialidase activity. Sialidase-1 and -3 immunoreactivities can be detected on the acrosomal region of spermatozoa. The surface expression of sialidase increased significantly after capacitation. The involvement of sialidase in spermatozoa-ZP interaction was demonstrated by the reduced capacities of the spermatozoa in binding to ZP and undergoing ZP-induced acrosome reaction after sialidase treatment. To conclude, our studies provide evidence that human sperm-ZP interaction involves coordinated actions of different proteins.

ACKNOWLEDGEMENTS: This work was supported in part by grants from the Committee on Research and Conference Grant, The University of Hong Kong, Hong Kong Research Grant Council (764512M, 764611M and 201109176154).

### 0186

### Basigin-integrin β1 complex promotes the trophoblast invasion during early placental development

Reproductive Endocrinology

Y. Guo, C.-L. Lee, W. S. B. Yeung and P. C. N. Chiu

*Department of Obstetrics and Gynaecology, The University of Hong Kong, Hong Kong*

The placenta is the interface between the mother and the fetus. Placentation involves trophoblast migration and invasion into decidua. Extravillous cytotrophoblast (EVCT) is a differentiated trophoblast subtype. Its invasion from basal lamina of the anchoring villous to extracellular matrix-rich decidualized stroma

© 2016 The Authors. Journal of Diabetes Investigation published by
Asian Association for the Study of Diabetes (AASD) and John Wiley & Sons Australia, Ltd is critical to human placentation. Dysregulation in EVCT invasion is associated with pregnancy-associated disease such as spontaneous abortion, fetal growth restriction and preeclampsia.

We have previously demonstrated a typeItransmembrane glycoprotein on human trophoblast named basigin is involved in trophoblast invasion and differentiation [1]. Reduced basigin expression in human placenta has been correlated with preeclampsia [2]. The transmembrane domain of basigin contains charged glutamic acid and typical leucine zipper motif, suggesting that basigin has high affinity to other transmembrane proteins in form of complexes. It is supported by our observation that basigin exists as protein complexes on the plasma membrane of human trophoblast [1]. We have also identified integrin β1 as an interacting partner of basigin on the human trophoblast. The objective of the present study is to evaluate the interaction between basigin and integrin β1 and the biological relevance of this interaction.

By using co-immunoprecipitation, basigin was demonstrated to complex with integrin β1. The interaction between basigin and integrin β1 in trophoblast was further confirmed by blue native-gel electrophoresis technique. Suppression of basigin by siRNA down-regulated the integrin β1 surface expression in human trophoblast. In contrast, integrin β1 suppression had no effect on basigin expression. Reduced basigin or integrin β1 expression was associated with decreased Wnt/β-catenin signaling pathway activation and trophoblast invasion. Interestingly, the inhibitory effect of integrin β1 suppression on trophoblast invasion was not able to be opposed by basigin activation, indicating that integrin β1 might be the downstream regulator of basigin function. To summarize, our findings suggest that basigin may regulate trophoblast invasion via interaction with integrin β1 in form of protein complex.

### 0187

### Efficacy and safety of trelagliptin in patients with type 2 diabetes: a study of switching from daily administration of DPP4-inhibitor

Therapeutics of Diabetes

M. Murakami[1,2] and H. Morita[2]

[1]*Shizuoka Red Cross Hospital, Department of Diabetes and Endocrinology, Shizuoka City, Shizuoka, Japan,* [2]*2nd Department of Medicine, Hamamatsu University School of Medicine, Hamamatsu City, Shizuoka, Japan*

Objective: Trelagliptin Succinate was recently approved as the first long-acting dipeptidyl peptidase 4 inhibitor (DPP4-i) of once-weekly administration for treatment of type 2 diabetes in Japan. To assess the efficacy and safety of trelagliptin, a study of switching to trelagliptin from daily administration of DPP4-i was conducted in Japanese patients with type 2 diabetes.

Study Design and Method: We started the treatment of switching to 50 mg of trelagliptin once weekly in 54 patients who were already on daily administration of DPP4-I at least longer than 3 months combined with other oral antidiabetic agents (OADs) therapy including insulin. Daily doses of insulin and all other existing treatment were not titrated before and after switching to trelagliptin. After 8 weeks, the change in HbA1c level, daily dose of insulin, body weight and the incidence of symptomatic hypoglycemia were accessed in retrospective way.

Results: The patients were 67.8 years of mean age, and 22.3 of BMI with average duration of 12.7 years in diabetes. The mean level of HbA1c in baseline was 7.27% and slightly decreased to 7.15% at 8 weeks of trelagliptin administration. The mean daily dose of required insulin in baseline were 19.9 Units, and 19.5 Units also after 8 weeks, in 11 patients out of 54. The level of BMI were even slightly decreased, 23.3 in baseline and 22.2 after 8 weeks. Hypoglycaemic events were less common.

Conclusions: Switching to trelagliptin administration of once-weekly from daily DPP4-I treatment were effective to maintain the good glycaemic control and BMI properly and safe in association with less hypoglycaemic and weight gain over 8 weeks.

### 0188

### Glycodelin-A and IL-10 regulate human decidual macrophage differentiation from monocyte

Reproductive Endocrinology

M. Vijayan, C.-L. Lee, K.-F. Lee and P. C. Chiu

*Department of Obstetrics and Gynaecology, The University of Hong Kong, Hong Kong*

Introduction: To facilitate a successful pregnancy, it is important to confer immune tolerance to the semi-allogenic fetus. Macrophages are the second most abundant leukocytes (20~30%) in the decidua. Based on stimuli and function, macrophages are broadly classified as M1/inflammatory, M2/wound healing, and M3/regulatory macrophages. Gene expression profiling using microarray suggests that the decidual macrophages possess the phenotype of M2/M3 macrophages which secretes anti-inflammatory cytokines (e.g. IL-10) and immunomodulatory factors such as indoleamine 2,3-dioxygenase 1 (IDO-1) that contributes to immune tolerance. Aberrant levels of decidual macrophages are associated with fetal loss and pregnancy complications including intrauterine growth restriction and preeclampsia.

Glyodelin-A (GdA) is an immunomodulatory glycoprotein abundantly found in the decidua during early pregnancy. The most well studied role of GdA is on feto-maternal defense. It promotes Th2 cytokine shift and regulates the differentiation/functions of immune cells, trophoblasts and endometrial cells.

Hypothesis: We hypothesize that GdA is involved in the differentiation and biological functions of decidual macrophages.

Method: Macrophage colony stimulating factors (MCSF) with/without IL-10 or GdA were used to differentiate human peripheral blood derived monocytes into macrophages. The phenotype of the macrophages was analyzed by qPCR and flow cytometry. The conditioned medium of the differentiated macrophages was also collected for cytokine/immunomodulatory factor determination using membrane-based cytokine array, Western blotting and kynurenine assay. Flow cytometry-based latex beads assay was used to study the phagocytoic activity of differentiated macrophage.

Result: The results showed that GdA induced the expression of decidual macrophage markers indoleamine-2,3 dioxygenase (IDO-1) and CD209 during MCSF-induced monocyte differentiation. IL-10, another molecule known to induce the differentiation of monocytes into decidual macrophages, induced the expression of CD209 and another decidual macrophage marker, neuropilin-1 (NRP-1). Interestingly, co-treatment of GdA and IL-10 significantly increased the expression of all three decidual macrophage markers in the MCSF-differentiated macrophages. In addition, the conditioned medium of GdA-polarized macrophage exhibited a cytokine profile similar to that of decidual macrophages as demonstrated by cytokine array. GdA-polarized macrophages have a lower phagocytoic activity when compared to the control.

Conclusion: To conclude, decidual GdA polarizes the differentiation of human monocyte towards decidual macrophage. Our data also indicates that GdA and IL-10 had additive effect on decidual macrophage differentiation. In addition, GdA regulates the biological functions of macrophages, including IDO-1 activity/production, phagocytosis and cytokine expression.

Acknowledgements: This work was supported in part by grants from the Committee on Research and Conference Grant, Hong Kong Research Grant Council (HKU 774212M).

### 0189

### Inflammatory condition inhibits adrenomedullin and its receptor expression in human oviductal epithelial Cells

Reproductive Endocrinology

X. Wang, C.-L. Lee, R. H. Li and P. C. Chiu

*Department of Obstetric and Gynecology, Li Ka Ching Faculty of Medicine, The University of Hong Kong, Hong Kong*

Introduction: Ectopic pregnancy occurs when an embryo implants outside of the uterus, the majority (>97%) of which occur in the oviducts. The exact mechanism leading to tubal implantation remains unknown. However, as embryo

© 2016 The Authors. Journal of Diabetes Investigation published by Asian Association for the Study of Diabetes (AASD) and John Wiley & Sons Australia, Ltd implantation in the uterus involves inflammatory events, the pro-inflammatory phenotype in the oviduct caused by infection and/or smoking is suggested to be the major risk factors for tubal ectopic pregnancy.

Our previous work demonstrated oviductal tissue from women with tubal ectopic pregnancy had reduced adrenomedullin (ADM) production. ADM is a peptide hormone abundantly expressed in the human oviduct. Its expression peaks at the early luteal phase when the developing embryo is being transport through the oviduct. ADM receptors compose of 2 transmembrane components, namely the calcitonin-receptor-like receptor (CRLR) and the receptor-activity-modifying protein (RAMP). ADM has well-known anti-inflammatory action. Thus, this study aims to determine the role of ADM on the inflammatory condition of oviduct.

METHOD: Our established immortalized oviductal epithelial cells, OE-E6/E7, was used as the oviduct model. Bacterial endotoxin lipopolysaccharide (LPS) and pro-inflammatory cytokine interleukin (IL)-1β treatments were employed to mimic the acute and chronic inflammatory conditions within the oviduct respectively. The mRNA expression and protein expression of ADM/ADM receptor components in OE-E6/E7 were studied by qPCR and Western-blotting/immunofluorescence staining respectively.

RESULT: The expression of ADM and ADM receptor components, including CRLR and RAMP2, were demonstrated in OE-E6/E7 cells. On the other hand, no RAMP3 mRNA and protein expression can be detected. We further showed that LPS or IL-1β treatment down-regulated the ADM/ADM receptor component expression, indicating that ADM may play a role in modulating the inflammatory condition within the oviduct.

CONCLUSION: To conclude, our data provide evidence that inflammatory condition may regulate the expression and biological activity of ADM within the oviduct. Continued investigation of the area may provide considerable understanding of the pathogenesis of tubal ectopic pregnancy.

ACKNOWLEDGEMENTS: This work was supported in part by grants from the Committee on Research and Conference Grant, Hong Kong Research Grant Council (17113314).

### 0190

### Effect of diabetes on proliferation and differentiation abilities of mesenchymal stem cells isolated from subcutaneous and visceral adipose tissue

Other Endocrine Topics

A. Ghorbani, A. Hosseini, M. Mojarad, A. Hatami and S. J. Hoseyni
*Mashhad University of Medical Sciences, Mashhad, Iran*

OBJECTIVE: Recent data suggest that there are intrinsic differences between different depots of adipose tissues. In this study, we examined whether proliferation and differentiation abilities of mesenchymal stem cells (MSCs) are different between subcutaneous and visceral adipose depots and if the abilities are affected by diabetes.

MATERIALS AND METHODS: Subcutaneous and visceral (epididymal) fat samples were harvested from normal rats. Then, the animals were given streptozotocin to induce diabetes and after 10 days subjected to fat removal again. The MSCs isolated from both fat depots were cultured in adipocyte and osteocyte differentiation medium. Then, degree of differentiation into adipocyte and osteoblast was determined using Oil Red O and Alizarin Red staining, respectively. Also the expression of adipocyte-specific mRNAs (lipoprotein lipas and peroxisome proliferator-activated receptor γ2, PPARγ2) and osteoblast-specific mRNAs (alkaline phosphatase and osteopontin) were evaluated using quantitative real-time PCR. The rate of cell proliferation was determined using MTT assay.

RESULTS: Before induction of diabetes, differentiation ability of subcutaneous MSCs was higher than that of visceral MSCs. After diabetes induction, both subcutaneous and visceral MSCs were able to differentiate into adipocytes and osteoblasts, but the level of differentiation was more significant in subcutaneous MSCs. The expression of PPARγ2 and lipoprotein lipas mRNA in adipocytes differentiated from subcutaneous MSCs was significantly higher than visceral MSCs, both before and after diabetes. Similarly, the expression of alkaline phosphatase and osteopontin in osteocytes differentiated from subcutaneous MSCs was higher than visceral MSCs. The rate of MSCs proliferation was higher in subcutaneous than in visceral fat, both before and after diabetes.

CONCLUSION: Our data showed that subcutaneous MSCs have higher proliferation and differentiation abilities than visceral MSCs in normal and diabetes condition. The depot-specific differences should be considered when designing a cell therapy approach using adipose tissue stem cells for non-diabetic or diabetic patients.

© 2016 The Authors. Journal of Diabetes Investigation published by
Asian Association for the Study of Diabetes (AASD) and John Wiley & Sons Australia, Ltd
